# What is *Atraphaxis* L. (Polygonaceae, Polygoneae): cryptic taxa and resolved taxonomic complexity instead of the formal lumping and the lack of morphological synapomorphies

**DOI:** 10.7717/peerj.1977

**Published:** 2016-05-03

**Authors:** Olga V. Yurtseva, Oxana I. Kuznetsova, Maria E. Mavrodieva, Evgeny V. Mavrodiev

**Affiliations:** 1 Faculty of Biology, Department of Higher Plants, M.V. Lomonosov Moscow State University, Moscow, Russia; 2 Herbarium, Tsitsin Main Botanical Garden, Russian Academy of Sciences, Moscow, Russia; 3 Eastside High School, Gainesville, Florida, USA; 4 Florida Museum of Natural History, University of Florida, Gainesville, Florida, USA

**Keywords:** Atraphaxis, Polygonum, Polygonaceae, Taxonomy, Molecular phylogeny, Perianth morphology, Sporoderm ornamentation

## Abstract

**Background:** The recently proposed recircumscription of the genus *Atraphaxis* (incl. *Atraphaxis* section *Ovczinnikovia* O.V. Yurtseva ex. S. Tavakkoli and *Polygonum* sect. *Spinescentia* Boissier (=*A.* sect. *Polygonoides* S. Tavakkoli, Kaz. Osaloo & Mozaff.) makes this genus fairly heterogeneous and therefore almost undefinable based on morphology. A critical comprehensive reappraisal of the group is necessary.

**Methods:** Using the DNA sequence data (ITS1&2 regions of nrDNA and combined *trn*L intron + *trn*L–F IGS and rpl32–*trn*L^(UAG)^ IGS regions of plastid genome), Maximum Likelihood (ML) and Bayesian analyses (BI) were applied for phylogenetic reconstructions of the tribe Polygoneae with special attention to *Atraphaxis*, and related taxa. Maximum parsimony reconstructions of the evolution of perianth morphology and sporoderm ornamentation in the tribe Polygoneae were also performed. Life history, morphology of shoots, leaf blades, ocreas, perianth and achene morphology, ultrasculpture of achene surface, and pollen morphology were compared, and SEM and LM images were provided.

**Principal findings:** The genera *Atraphaxis* and *Polygonum* were found to be widely polyphyletic. The rarest and morphologically remarkable endemic of Tian-Shan and Pamir *Atraphaxis ovczinnikovii* (*Atraphaxis* sect. *Ovczinnikovia* O.V. Yurtseva ex. S. Tavakkoli) was confirmed to be a sister of the clade (*Atraphaxis* + *Polygonum* sect. *Spinescentia*) in plastid topology. The genus *Bactria (=Atraphaxis* sect. *Ovczinnikovia*), which circumscribes two species, is newly established as a result of this analyses. Morphological data confirm the originality of the taxon.

**Discussion:** We are arguing for a narrow delimitation of *Atraphaxis* with petalloid segments and striato-perforate sporoderm ornamentation as morphological synapomorphies. The recently proposed inclusion of *Polygonum* sect. *Spinescentia* in *Atraphaxis* is fairly questionable from a morphological standpoint. The rank of *Polygonum* sect. *Spinescentia* requires further clarification. The generic composition of the tribe Polygoneae also requires future reappraisals.

## Introduction

The genus *Atraphaxis* L. (Linnaeus, 1753) (Polygonaceae) includes ca. 30 species distributed from South-East Europe and North-East Africa to East Siberia, China, and Mongolia with its centers of taxonomic diversity in South-West and Central Asia (Bentham & Hooker, 1880; Pavlov, 1936; Webb, 1964; Cullen, 1967; Rechinger & Schiman-Czeika, 1968; Lovelius, 1978; Lovelius, 1979; Borodina, 1989; Brandbyge, 1993; Cherepanov, 1995; Gubanov, 1996; Bao & Grabovskaya-Borodina, 2003; Baikov, 2012). Based on morphology, *Atraphaxis* was assigned to the tribes Polygoneae, Rumiceae, Atraphaxideae, or Calligoneae of the family Polygonaceae (taxonomic history is summarized in Table S1). However, strong similarity in petiole and stem anatomy supports the placement of *Atraphaxis* into the tribe Polygoneae Meisn. emend Haraldson along with *Polygonum* L., *Polygonella* Michx., *Oxygonum* Burch., *Pteropyrum* Jaub. et Spach, *Calligonum* L., and *Fagopyrum* Mill. (Haraldson, 1978), that greatly corresponds to the results of recent molecular studies, the latest of which revealed the composition of the tribe Polygoneae, subfamily Polygonoideae (Galasso et al., 2009; Schuster, Wilson & Kron, 2011a; Schuster, Reveal & Kron, 2011b). According to the phylogenetic analyses of Galasso et al. (2009), based on cpDNA sequence data (*rbc*L cpDNA region), the tribe Polygoneae comprises *Polygonum* (incl. *Polygonella*), *Atraphaxis*, *Calligonum*, *Oxygonum*, *Parapteropyrum* A. J. Li, *Pteropyrum*, *Muehlenbeckia* Meisn., *Homalocladium* (F. Muell.) L. H. Bailey, *Knorringia* (Czuk.) Tzvelev, *Fallopia* Adans., *Reynoutria* Houtt., and × *Reyllopia* Holub.

Another composition of *Polygoneae* was proposed by K.A. Kron and co-authors (Sanchez et al., 2011; Schuster, Wilson & Kron, 2011a; Schuster, Reveal & Kron, 2011b), who made a great contribution to the molecular phylogenetics of *Polygonaceae*. Their approaches were based on the sequence data of several chloroplast (*mat*K, *ndh*F, 3′*rps*16–5′*trn*K, *trn*L–*trn*F, 3′*trn*V–*ndh*C) and nrDNA (2nd intron of *LEAFY* (lfyi2), nr ITS) regions, and showed that the tribe Polygoneae is composed of the genera *Knorringia*, *Fallopia*, *Muehlenbeckia* (incl. *Homalocladium*), *Reynoutria*, *Duma* T.M. Schust., *Polygonum* (incl. *Polygonella*), and *Atraphaxis*.

The previous morphological analyses of the open thyrses with axillary cymes of flowers (Gross, 1913), and flower and fruit structure (Ronse De Craene & Akeroyd, 1988; Ronse De Craene, Hong & Smets, 2000), placed *Atraphaxis* close to the genera *Polygonum* and *Polygonella*, and were in general agreement with these molecular-based results. Similarly to *Polygonum*, *Atraphaxis* has 6–8 stamens with flattened filaments, which are dilatated at the base and form a glandular ring (Linnaeus, 1753; Boissier, 1879; Ronse De Craene & Akeroyd, 1988; Brandbyge, 1993). The genera *Atraphaxis* and *Polygonum* also share segments with a dendricularly branched midvein, but the basal lateral veins are anastomosing in neighbour segments in *Atraphaxis* and not anastomosing in *Polygonum* (Vautier, 1949). The embryo is bent along one of the achene ribs in *Atraphaxis* and *Polygonum*, but is straight in a central position in *Polygonella* (Krasnov, 1888).

Linnaeus (1753: page 333) described the genus *Atraphaxis* based on *A. spinosa* L. with a tetrameric perianth, 6 stamens and a dimeric gynoceum. Later, Marschall Bieberstein (1819: 284) established the genus *Tragopyrum* M. Bieb. with a pentamerous perianth consisting of two sepals and three petals persistent, tightly surrounding the fruit, achene glabrous, trigonous (“calyx 2-phyllus, petala 3, persistentia, circa fructum conniuentia, semen nudum, triquetrum”).

The recent taxonomic composition of *Atraphaxis* was suggested by Jaubert & Spach (1844–1846), who merged the *Atraphaxis* sensu Linnaeus (1753) with *Tragopyrum* (classification history is summarized in Table S2). In addition to the sections *Atraphaxis* and *Tragopyrum* (M. Bieb.) Meisn., Lovelius (1979) described the monotypic section *Physopyrum* (Popov) Lovelius, including *A. teretifolia* (Popov) Kom. with terete leaves and concave inner segments of the perianth.

The majority of authors supported the division of *Atraphaxis* into two groups based on flower merosity, the first of which was comprised of species with dimeric perianths and lenticular achenes, and the second—of species with pentamerous perianths and trigonous achenes (Table S2).

The molecular phylogenetic reconstructions of *Atraphaxis*, based on cpDNA sequence data (*atp*B–*rbc*L, *psb*A–*trn*H, *trn*L–*trn*F, *psb*K–*psb*I, and *rbc*L) (Sun & Zhang, 2012; Zhang et al., 2014), have also divided *Atraphaxis* into two clades, the first one combines the species with tetramerous and pentamerous perianth, and the second one—the species with pentamerous perianth. However, the latest phylogenetic treatment by Tavakkoli et al. (2015) did not confirm this division.

Our original phylogenetic reconstructions of the tribe *Polygoneae*, based on ITS nrDNA sequence data, recovered polyphyly of *Polygonum* L. s.l. (Yurtseva et al., 2010), with *P. arianum* Grigorj., *P. atraphaxiforme* Botsch., and *P. toktogulicum* Lazkov nested in the clade *Atraphaxis*. These species were later transferred by Schuster, Reveal & Kron (2011b) into the genus *Atraphaxis* L. as *A. ariana* (Grigorj.) T.M. Schust. & Reveal, *A. atraphaxiformis* (Botsch.) T.M. Schust. & Reveal, and *A. toktogulica* (Lazkov) T.M. Schust. & Reveal. They differ from other species of *Atraphaxis* by a campanulate perianth with five ovate to oblong-elliptical equal-sized segments and a short funnel-form tube (Grigorjev, 1933; Botschantzev, 1965; Lazkov & Sultanova, 2002), whereas typical *Atraphaxis* are characterized by a perianth with a long filiform tube and 4–5 broadly-ovate, orbiculate, or reniform segments, of which the inner ones are significantly accrescent in fruiting and enclose the achene, while the outer segments are small and reflected to a pedicel (Jaubert & Spach, 1844–1846). Like all other species of the genus *Atraphaxis* (Hong, 1995), *A. atraphaxiformis* and *A. ariana* are characterized by striate-perforate sporoderm ornamentation, only *A. toktogulica* has a unique reticulato-perforate sporpoderm ornamentation with hardly visible smoothened striae (Yurtseva, Severova & Bovina, 2014).

The rarest *Polygonum ovczinnikovii* Czukav., initially described as an endemic of the Pamir mountains, is a small shrub with a campanulate perianth of 5(6) equal-sized segments (Chukavina, 1962; Chukavina, 1968; Chukavina, 1971) and sporoderm ornamentation described as reticulato-foveolate (Yurtseva, Severova & Bovina, 2014). This taxon has been circumscribed with *Atraphaxis* under the name *A. ovczinnikovii* (Chukav.) O.V. Yurtseva (Yurtseva, Severova & Bovina, 2014) and was suggested to add a new section (Yurtseva et al., 2012a).

Another remarkable dwarf shrub has been collected in Tien Shan (Kyrgyzstan, Naryn reg., Dzhumgal distr., 7 July 2006, *Lazkov 24*) and also initially assigned as *Polygonum ovczinnikovii*. It was shown to be a sister to *Atraphaxis* in phylogenetic reconstructions based on ITS data (Yurtseva et al., 2012a; Yurtseva et al., 2012b).

Because of the most recent results of Maximum Likelihood (ML) and Bayesian (BI) analyses of the ITS and plastid sequence data (*rpl*32 and *mat*K), Tavakkoli et al. (2015) proposed the recircumscription of the genus *Atraphaxis*, including the members of traditionally accepted sections *Atraphaxis* and *Tragopyrum*, and two newly described sections: *A*. sect. *Ovczinnikovia* O.V. Yurtseva ex S. Tavakkoli and *A*. sect. *Polygonoides* S. Tavakkoli, Kaz. Osaloo & Mozaff (Tavakkoli et al., 2015). In the molecular topologies by Tavakkoli et al. (2015), the latter section corresponds to the clade that includes several endemics of Iran, which are traditionally treated as part of the genus *Polygonum* s.l., not *Atraphaxis*: *P. aridum* Boiss. & Hausskn., *P. botuliforme* Mozaffarian, *P. dumosum* Boiss., *P. spinosum* H. Gross., *P. khajeh-jamali* Khosravi & Poormahdi, and *P. salicornioides* Jaub. & Spach (Tavakkoli et al., 2015). All of these plants are fairly notable caespitose-subshrubs with linear-lanceolate, rarely terete leaves, a campanulate perianth that does not fully enclose the achene, and reticulate-perforate sporoderm ornamentation (Tavakkoli et al., 2015). *Polygonum dumosum* and *P. salicornioides* were previously attributed to polyphyletic *Polygonum* sect. *Avicularia* Meisn. subsect. *Suffruticulosa* Meisn. (Meisner, 1857: 89); these two species and *P. aridum* formed *Polygonum* sect. *Avicularia* Meisn. subsect. *Spinescentia* Boiss. (Boissier, 1879: 1042). The latter group with a narrow understanding of the genus *Polygonum* L. s.str. (= *P.* sect. *Avicularia* Meisn.) hereinafter is named as *Polygonum* sect. *Spinescentia* Boiss.

In the ITS-based analysis by Tavakkoli et al. (2015), the *Atraphaxis ovczinnikovii*, collected from the Tian Shan area, appeared as a sister group of the clade (*Atraphaxis* s.str. + *A.* sect. *Polygonoides*). As a result, *Atraphaxis* sensu Tavakkoli et al. (2015) combines the following taxonomic entities: (1) *Atraphaxis*, as treated traditionally; (2) *Polygonum* sect. *Spinescentia* (=*A*. sect. *Polygonoides*), and (3) *Atraphaxis* sect. *Ovczinnikovia.*

In other words, the genus *Atraphaxis* emend S. Tavakkoli combines the taxa with different ornamentation of sporoderm and also with fairly different perianth morphology, varying from the perianth common for *Atraphaxis* (Jaubert & Spach, 1844–1846), to the campanulate or urceolate perianth with five equal-sized segments (typical for *Polygonum* sect. *Avicularia* sensu Meisner, 1857).

The first comprehensive diagnosis of *Atraphaxis*, given by Jaubert & Spach (1844–1846: Tab. 110), describes the perianth of *Atraphaxis* as “subpetaloid, persistent, enlarged (later membranous, with a net of veins), or divided in four or five segments imbricate in the flower bud, spreading in the flowering stage; segments obtuse, in two circles, unequal, connate at base: two outer ones small, after the flowering stage reflexed or recurved, concave, mostly different from the inner ones; two or three inner segments major, erect and tightly adpressed to the fruit after flowering”; “pedicels filiform, with articulation around the middle, either glabrous, or surrounded by ochreolae, with expanded top extended into the flower” (“subpetaloideum, persistent, accrescent (demùm scariosum, reticulatum), aut 5-aut 4-partitim, aestivatione imbricatum, sub anthesi patens; sepala obtusissima, biserialia, inaequalia, basi concreta: 2 externa, minora, post anthesin reflexa v. recurva, concava, plerumque internis dissimilia; 2 v. 3 interna, majora, post anthesin erecta, conniventia, fructû adpressa”; “pedicelli capillares, juxtâ v. infrè v, suprè medium noduloso-articulati, aùt nudi, aùt ocreolâ propriâ stipati, apice subclavato cum flore continui”).

In contrast, *Polygonum* L. s.str. (=*P.* section *Avicularia* sensu Meisn.) was characterized by Meisner (1857: 85) as follows: “calyx subpetaloid, outer segments (often keeled) in the middle herbaceous,” “achene included into enlarged dry calyx (rarely tip is protruded)” (“calix semicorollis, lobis exterioribus (saepe carinatus) vel omnibus medio herbaceis,” “achaenium calyce sicco parum aucto aptero inclusum (raro apice exsertum)”).

To summarize, from traditional standpoint, the most significant difference between the genus *Atraphaxis* and *Polygonum* is the morphology of the perianth with equal-sized segments not enlarged in fruiting stage in *Polygonum*, and with the inner two or three segments accrescent and surrounding the achene in *Atraphaxis* (used, for example, by Brandbyge (1993) and Li et al. (2003)).

Therefore, it seems clear that the recent formal proposal by Tavakkoli et al. (2015) makes the technically monophyletic genus *Atraphaxis* extremely heterogeneous and almost undefinable based on morphological grounds.

*Atraphaxis* sect. *Ovczinnikovia* O.V. Yurtseva ex. S. Tavakkoli was based solely on position of the single accession *A. ovczinnikovii* from Tien Shan in the ITS-based phylogenetic reconstruction by Tavakkoli et al. (2015). However, a more careful comparison of the accession *A. ovczinnikovii* from Tien Shan (Kyrgyzstan) with the specimens of *A. ovczinnikovii* from Pamir-Alay (Tajikistan), corresponding to *P. ovczinnikovii*, showed prominent differences in their morphology, clearly demonstrating, that they represent two different taxa.

Also, if the recently described *Atraphaxis* sect. *Polygonoides* will be treated as part of *Polygonum* (as it is supposed to be based on the morphology of the perianth), then the genus *Atraphaxis*, as circumscribed by Tavakkoli et al. (2015), will appear to be polyphyletic, with the sistership of *A.* sect. *Ovczinnikovia* to the clade (*Atraphaxis* s.str. + *Polygonum* sect. *Spinescentia* (=*A*. sect. *Polygonoides*)).

Therefore, the details of the phylogenetic placement of *A.* sect. *Ovczinnikovia* must be clarified, and, as a result, a more accurate taxonomical approach to the genus *Atraphaxis* must be established as a frame for future studies of the whole complex.

Our initial aim was to recover the phylogenetic placement of different accessions of *Atraphaxis ovczinnikovii* from Pamir-Alay and Tien-Shan in the tribe Polygoneae based on the analyses of combined cp DNA (*trn*L intron + *trn*L–F IGS and *rpl*32–*trn*L^(UAG)^ IGS) and nuclear ITS sequences. Later, our aim was to compare the morphological characteristics of the species, which a) had been traditionally placed in *Atraphaxis* and b) had been included recently.

## Material and Methods

### Plant Material

The morphological study involved 20 specimens of *Atraphaxis* and *Polygonum* from field collections, and ca. 1,000 specimens stored in the herbaria of V.L. Komarov Botanical Institute RAS, St. Petersburg, Russia (LE); Lomonosov Moscow State University, Moscow, Russia (MW); Tsitsin Main Botanical Garden RAS, Moscow, Russia (MHA); and Main Botanical Garden, National Academy of Science, Bishkek, Kyrgyzstan (FRU). For scanning electron microscopy (SEM), 26 specimens of 19 species of *Atraphaxis* and *Polygonum* were used (Data S1). For light microscopy (LM) 18 specimens of 12 species of *Atraphaxis* and *Polygonum* were used (Data S2).

Identification of the samples used in the study was conducted after the examination of the type specimens of *Atraphaxis* and *Polygonum* (LE, MW), or the images (P—https://science.mnhn.fr/taxon/genus/atraphaxis, LINN—http://linnean-online.org/linnaean_herbarium.html, B—http://ww2.bgbm.org/herbarium/).

**Figure 1:**
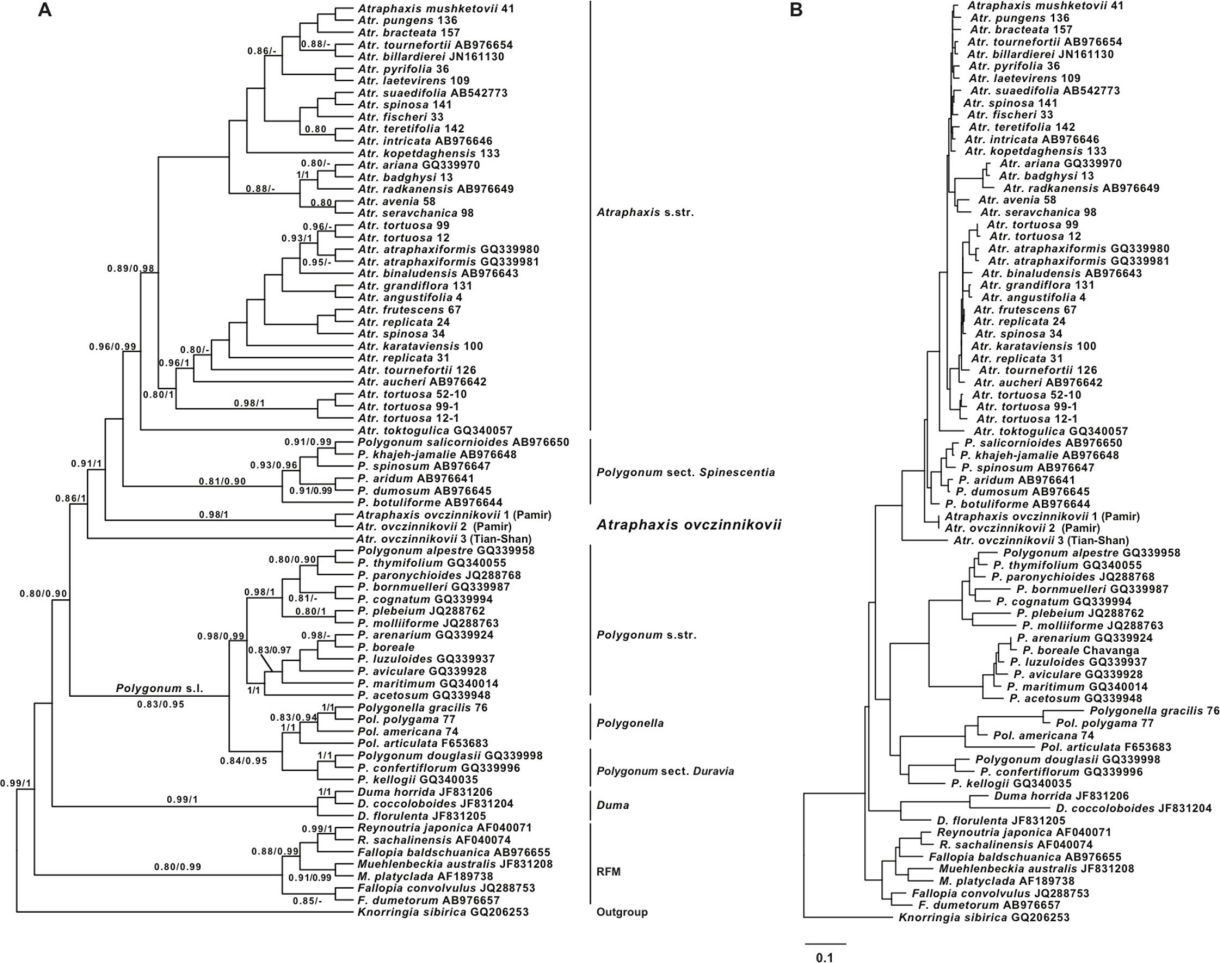
ITS phylogeny of the tribe Polygoneae. (A) the best tree from ML analysis of the ITS dataset (−log likelihood: 7894.75825). (B) the same tree with the branch lengths. Numbers above the branches indicate the aLRT support values equals or more than 0.8 from ML analysis/posterior probabilities equals or more than 0.9 from the BI of the same Matrix. Images: E. Mavrodiev.

**Figure 2:**
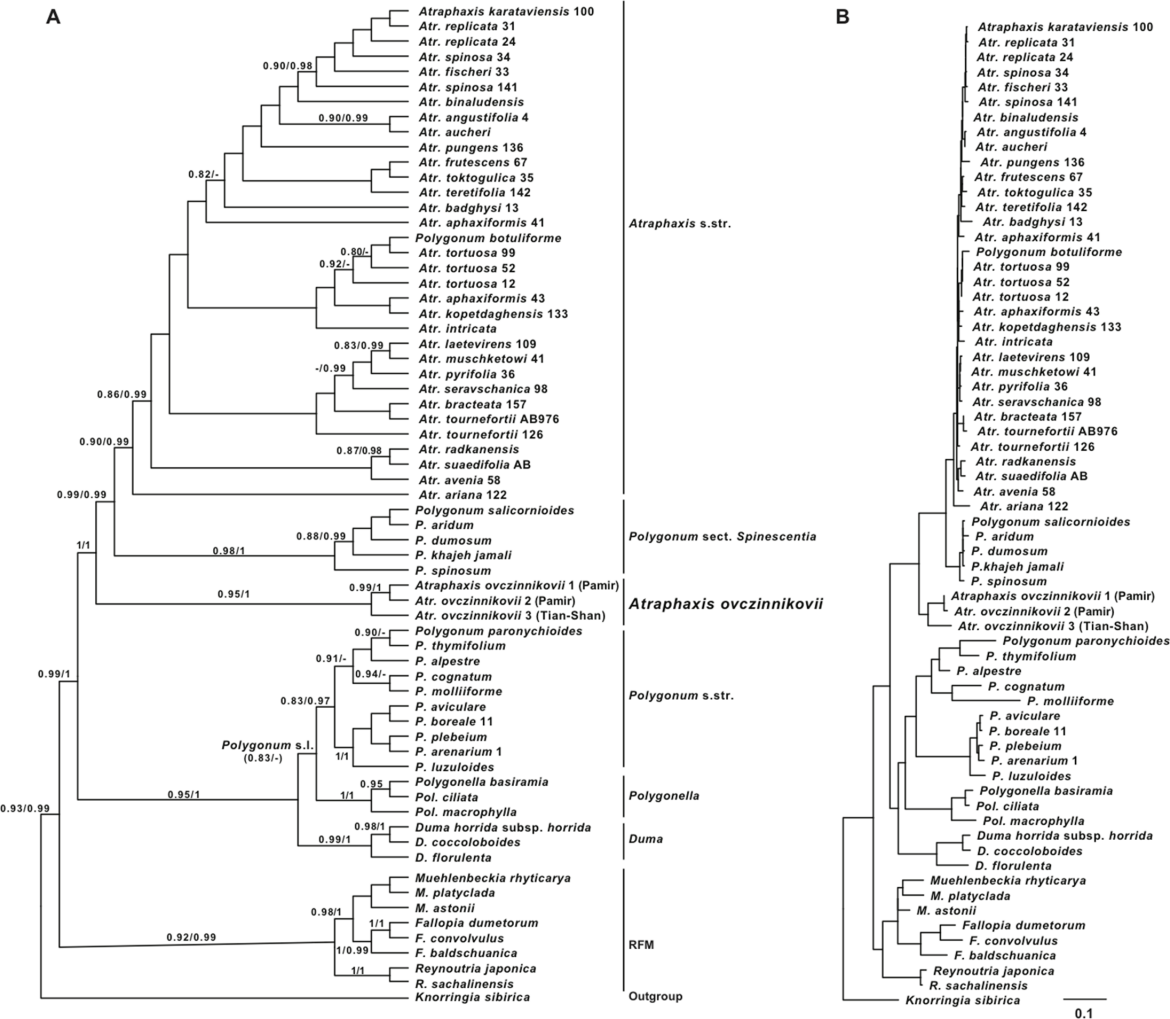
Plastid phylogeny of the tribe Polygoneae. (A) the best tree from ML analysis of combined plastid dataset: *trn*L intron + *trn*L–F IGS and *rpl*32–*trn*L^(UAG)^ IGS regions of cpDNA (−log likelihood: 12644.7663). (B) the same tree with the branch lengths. Numbers above the branches indicate the aLRT support values equals or more than 0.8 from ML analysis/posterior probabilities equals or more than 0.9 from the BI of the same Matrix. Images: E. Mavrodiev.

The molecular study involved all of the genera of the tribe Polygoneae, as circumbscribed by Schuster, Reveal & Kron (2011b): genera *Atraphaxis* s.l. (two accessions *A. ovczinnikovii* collected in *locus classicus* (Pamir-Alay), as well as the single accession from Tien Shan (which had also been analyzed), *Polygonum* s.l. (incl. *Polygonum* sect. *Spinescentia*), *Polygonella, Duma, Muehlenbeckia, Fallopia, Reynoutria*, and *Knorringia*. Based on the results of Schuster, Reveal & Kron (2011b), *Knorringia* has been chosen as an outgroup for the *a posteriori* rooting of the molecular topologies (Figs. 1 and 2). Table S3 contains information on the taxa and GenBank accession numbers used in the study.

### DNA isolation and amplification

DNA was extracted from the herbarium specimens using a NucleoSpin Plant Extraction Kit (Macherey-Nagel, Germany) with the yield of DNA ranged from 0.005–0.1 mg per 0.1 g of plant material.

The nrDNA ITS, and cpDNA *trn*L intron^(UAA)^ + *trn*L–F IGS regions and *rpl*32-*trn*L^(UAG)^ IGS region were used because of their utility in Polygonaceae and high variability (Schuster, Wilson & Kron, 2011a; Schuster, Reveal & Kron, 2011b; Tavakkoli, Kazempour Osaloo & Maassoumi, 2010; Tavakkoli et al., 2015). The primers of Taberlet et al. (1991) and Shaw et al. (2007) were used for amplification of these regions. The nrDNA ITS1&2 region was amplified using external primers ITS1 and ITSB, and, in some cases, internal primers ITS2 and ITS3 (White et al., 1990; Milyutina et al., 2010).

The PCR was performed in 0.02 ml of a mixture contained 10–20 ng DNA, 5 pmol of each primer and MaGMix (Dialat LTD, Russia), contained 0.2 mM of each dNTP, 2.0 mM MgCl_2_, 2.5 units of Smart Taq polymerase. 1.0 mM of DMSO was included for amplification of nrDNA regions with high CG content.

Amplification of nrDNA ITS and cpDNA *trn*L intron^(UAA)^ + *trn*L–F IGS regions was performed under the following program: hold 95 °C, 3 min; 94 °C, 30 s; 58 °C, 30 s; 72 °C, 30 s; repeat 30–33 cycles; extend 72 °C, 3 min. Amplification of *rpl*32-*trn*L^(UAG)^ IGS region was performed using the following program: hold 95 °C, 3 min; 94 °C, 30 s; 52 °C, 30 s; 72 °C, 60 s; repeat 35 cycles; extend 72 °C, 5 min.

### Purification of PCR products and DNA sequencing

Amplification products were purified by electrophoresis (Sambrook, Fritsch & Maniatis, 1989), one-band DNA fragments were extracted from the gel and purified using the GFX™ PCR DNA, Gel Band Purification Kit (GE HealthCare, Little Chalfont, UK), or Evrogene Cleanup Mini (Russia), and then used as a template in sequencing reactions with the ABI Prism BigDye Terminator Cycle Sequencing Ready Reaction Kit (Applied Biosystems, Foster City, CA, USA) following the standard protocol provided for 3100 Avant Genetic Analyzer (Applied Biosystems, Foster City, CA, USA). Fragment sequences were determined at the Genom Center (Engelhardt Institute of Molecular Biology RAS, Moscow, Russia).

### Cloning

Purified ITS amplificates from three plants of *A. tortuosa* were ligated into the pBluescript KS+ vector and were cloned in *Escherichia coli* NM 522 cells. The lysed colonies containing recombinant plasmids were used in the amplification reaction with M13/pUC sequencing primers and the resulting amplificates were sequenced with the original ITS1&2 PCR-primers.

### Sequences used in phylogenetic analyses and the alignment strategy

Voucher information, current and GenBank numbers are presented in Table S3.

All sequences were aligned using MAFFT (Katoh et al., 2002; Katoh & Standley, 2013) following MAFFT’s L-INS-i alignment strategy (Katoh et al., 2002; Katoh & Standley, 2013), with the default settings set for gap opening penalty and the offset value.

A total of 76 sequences of the nrDNA ITS1&2 region, representing the genera: *Atraphaxis* s.l. (38, including three clones from *A. tortuosa*), *Polygonum* (16), *Polygonum sect. Spinescentia* (7); *Polygonella* (4), *Duma* (3), *Fallopia* (3), *Muechlenbeckia* (2), *Reynoutria* (2), and *Knorringia* (1) were analyzed. Among them, 30 sequences were elaborated for this study, 22 sequences were obtained previously (Yurtseva et al., 2010; Yurtseva et al., 2012b), and 24 sequences were downloaded from GenBank (http://www.ncbi.nlm.nih.gov). The aligned matrix for nrDNA ITS1&2 region includes 701 characters.

Aligned plastid matrices were manually concatenated and analyzed as a single contiguous dataset. The combined aligned chloroplast matrix for cpDNA *trn*L intron + *trn*L-F IGS and *rpl*32-*trn*L^(UAG)^ IGS regions includes 2,769 characters and 66 accessions from the genera *Atraphaxis* (34), *Polygonum* (10), *Polygonum* sect. *Spinescentia* (7), *Polygonella* (3), *Duma* (3), *Fallopia* (3), *Muehlenbeckia* (3), *Reynoutria* (2), and *Knorringia* (1). 33 accessions of the *trn*L intron^(UAA)^ + *trn*L–F IGS region were elaborated for this study and 17 accessions were downloaded from GenBank. For cpDNA *rpl*32-*trn*L region 28 accessions were obtained for this study and 22 ones were downloaded from GenBank.

### Phylogenetic analyses

The Maximum Likelihood analysis was performed with the PhyML v. 3.0 (Guindon & Gascuel, 2003; Guindon et al., 2010) following the automatic Smart Model Selection (SMS) option, with an estimated proportion of invariable sites and empirical nucleotide equilibrium frequencies. We took a BioNJ tree as a starting tree, and defined the strategy of the tree topology search as “best of NNIs and SPRs” following with ten random starts. Branch supports were calculated with the approximate likelihood-ratio test (aLRT) (reviewed in Guindon et al. (2010)). The GTR + G model was selected by PhyML-SMS as the best choice based on both Akaike and Bayesian information criteria for both plastid and ITS data matrices.

Following the general assumptions of the same model, the Bayesian analyses of the ITS and plastid matrices were conducted with the MrBayes (v. 3.1.2) (Ronquist & Huelsenbeck, 2003) as implemented in CIPRES (Miller, Pfeiffer & Schwartz, 2010). Two runs with four chains each (three heated and one cold) were run for 10 million generations; the chains were sampled every 1,000 generations with a default parameter.

### Morphological analysis

In total, 8 morphological characters are discussed below. Specific attention has been paid to the most important diagnostic traits of *Polygoneae* (the perianth morphology and the ornamentation of the sporoderm).

For the analysis of perianth morphology of *Polygoneae*, personal observations (summarized in Tables 1 and S4), and reference data (summarized in Table S5) on the genera *Polygonum* and *Atraphaxis* (Yurtseva et al., 2010; Yurtseva et al., 2012a; Yurtseva et al., 2012b; Yurtseva, Severova & Bovina, 2014), *Fallopia, Reynoutria* (Brandbyge, 1993; Ronse De Craene & Akeroyd, 1988), *Knorringia* (Hong, 1989); *Muehlenbeckia* (Brandbyge, 1992), *Duma* (Brandbyge, 1992; Schuster, Wilson & Kron, 2011a), *Polygonum* and *Polygonella* (Horton, 1963; Ronse De Craene & Akeroyd, 1988; Ronse Decraene, Hong & Smets, 2004), *Polygonum* sect. *Spinescentia* (Jaubert & Spach, 1844–1846; Boissier, 1846; Boissier, 1879; Meisner, 1857; Gross, 1913; Mozaffarian, 1988; Mozaffarian, 2012; Khosravi & Poormahdi, 2008; Tavakkoli et al., 2015) were used.

**Table 1:** Morphological characteristics of *Bactria lazkovii, B. ovczinnikovii, Polygonum* sect. *Spinescentia,* and *Atraphaxis*.

Characters	*Bactria lazkovii* (=*A. ovczinnikovii* from Tien-Shan)	*Bactria ovczinnikovii* (=*A. ovczinnikovii* from Pamir)	*Polygonum* section *Spinescentia*	*Atraphaxis*
Life history	Dwarf shrub 10–15 cm tall	Small shrub 20–30 cm tall	Cushion-shaped or caespitose dwarf shrubs or undershrubs 10–15 cm tall	Dwarf or tall shrubs, rearely undershrubs from 20–30 to 100–300 cm tall
Annual shoots	Elongated, puberulent	Elongated, puberulent	Elongated, puberulent	Elongated and constricted
Shape of leaf blade	Oblong-elliptical or lanceolate	Broadly-ovate or rhomboid-elliptical	Linear-elliptical, linear-lanceolate, ovate, oblong-lanceolate, sausage-shaped	Rotundate, broadly ovate, broadly-elliptical, oblong-elliptical, linear-lanceolate, terete
Leaf margin	Revolute	Revolute	Revolute	Finely crenulate, undulate, flat or slightly revolute
Ochrea in thyrses	Cup-shaped inflated under the petiole, 4–5 mm long	Cup-shaped inflated under the petiole, 2–4 mm long	?	Oblique funnel-form, with reduced leaf blade or a keel, 2–7 mm long
Ochrea length and shape at vegetative shoots	2–4 mm, lanceolate, later bilacerate, with two veins	2–4 mm, lanceolate-tubulate, later bilacerate, without veins	3–5 to 6–10 mm, tubulate, truncate, later bidentate, with 0–6 veins	3–5 to 7–10 mm, tubulate, later lacerate in short middle part and two linear lacinulae, with two veins
Position of thyrses	Terminal	Terminal	Terminal	Terminal or lateral
Thyrses	Frondulose, 2–3 axillary cymes of 1–2 flowers	Frondulose, 5–7 axillary cymes of 1–2 flowers	Frondose, frondulose or bracteose, 3–5 cymes of 1–2 flowes	Bracteose, rarely frondulose, from 6–10 to 10–20 cymes of 1–2 flowers
Perianth shape in fruiting	Campanulate, with equal-sized segments not enclosing the achene	Campanulate, with equal-sized segments not enclosing the achene	Campanulate, with funnel-form tube, enclosing the achene or not	Campanulate, with equal-sized segments or inner segments strongly enlarged in fruits and enclosing the achene
Perianth length, mm	2.0–2.5, in fruit 2.5–3.0	3.0–4.0, in fruit 4.0–5.0	3.0–6.5	6.5–14.5
Perianth partition	4/5–5/6	5/6–8/10	2/3–3/4	1/2–3/4 (9/10 in *A. teretifolia*)
Segment size	Equal	Equal	Equal	Equal, subequal or unequal
Segment shape	Elliptical or broadly-ovate, outer slightly acuminate and cucullate, without keel, inner flat, obtuse	Lanceolate, acuminate, outer concave, keeled, cucullate, inner almost flat	Oblong-elliptical, oblong-ovate, lanceolate, gradually acuminate or obtuse, flat	Rotundate, reniform, broadly-elliptical, broadly-ovate, obtuse, flat, undulate, rarely concave
Segment consistence	Petalloid	Coriaceous	Coriaceous, rigid	Petalloid
Perianth surface	Glabrous	Glabrous	Shortly puberulent, glabrous only in × *P. botuliforme*	Glabrous or rarely shortly papillate at tube and segment bases
Segment edge	Papillate	Papillate	Papillate or not	Papillate or not
Stomata at segments	Present	Present	Present	Absent or rarely present only at base
Perianth tube shape	Cup-form, sacciform	Funnel-form	Funnel-form	Filiform, with wedge-shaped or cup-shaped extension at the top
Tube length, mm	0.5–0.8	0.5–0.6	1.6–2.2	0.5–7.5
Length of filiform part of tube, mm	0	0.10–0.15	0.3	0.5–7.0
Achene size	2.5–3.0 × 1.8–2.0	4.0–5.0 × 2.2–2.8	3.5–4.0 × 2.8–3.4	2.5–5.5 × 1.5–5.0
Achene shape	Ovoid, triquetrous, with obtuse ribs	Ovoid, triquetrous with distinct ribs	Ovoid, triquetrous with distinct ribs	Ovoid, triquetrous or lenticular
Styles and stigma	Free, capitate	Connate at base, capitate	Connate at base, linear with inflated stigmae	Connate at base or free, capitate, fimbriate
Achene surface	Smooth or smooth-pitted	Smooth or smooth-pitted	Minutely tuberculate or smooth	Smooth, smooth-pitted, minutely rugulate or tuberculate
Sporoderm surface	Foveolate- perforate, with rounded pits smoothened at edges, some with 1–2 large perforations 0.5–1.5 μm in diam.	Microreticulate-foveolate, with 4–6-angular pits sharply defined at edges, rarely perforated (0.1–0.2 μm in diam.)	Reticulate-perforate, tectate-perforate, striate-perforate (*P. botuliforme*)	Striate-perforate, rarely reticulato-perforate (*A. toktogulica*)

Eventually the observed perianth morphology of Polygoneae was formalized using the following five character states: 0—campanulate divided to 1/2 in 5 equal-sized segments, with a short tube; 1—campanulate divided to 2/3–3/4 in 5 equal-sized segments, with a short tube; 2—campanulate divided to 4/5–5/6 in 5 equal-sized segments, with a short tube; 3—campanulate divided to 8/10–9/11 in 5 equal-sized segments, with a short tube; 4—divided in 4–5 segments, the outer ones smaller than the inner ones, with a long filiform tube; 5—divided in 4–5 segments, the outer segments larger than the inner ones, with a long filiform tube.

The ornamentation of the sporoderm in the tribe Polygoneae was formalized using the following eight character states, some corresponding to previously described palynotypes (summarized in Table S6): 0—palynotype *Avicularia*, psilate, micropunctate, microspinulose (Hedberg, 1946; Hong, Oh & Ronse De Craene, 2005; Brandbyge, 1992); 1—microreticulato-foveolate with 4–6 angular pits and rare small perforations 0.1–0.2 μm in diameter; 2—foveolate-perforate with rare perforations 0.5–1.5 μm in diameter; 3—reticulato-perforate, *A. toktogulica* (Yurtseva, Severova & Bovina, 2014), *P. salicornioides, P. aridum* (Tavakkoli et al., 2015); 4—striato-perforate, *Atraphaxis* (Hong, 1995; Yurtseva, Severova & Bovina, 2014); 5—palynotype *Duravia*, dimorphic: rugulate or foveolate with microspinules around the colpi, semitectate-reticulate at mesocolpia (Hedberg, 1946; Hong, Oh & Ronse De Craene, 2005); 6—palynotype *Pseudomollia*, dimorphic: psilate around the colpi, verrucate at 1/3 mesocolpia and poles (Hong, Oh & Ronse De Craene, 2005); 7—palynotype *Fallopia*, dimorphic: microspinulose around the colpi and psilate or punctate at mesocolpia and poles (Nowicke & Skvarla, 1977; Nowicke & Skvarla, 1979; Van Leeuwen, Punt & Hoen, 1988); 8—rugulate, *Knorringia* (Hong, 1989), *Reynoutria* (unpublished), part of *Muehlenbeckia* (Nowicke & Skvarla, 1977; Nowicke & Skvarla, 1979).

### The optimization of morphological traits

Using topologies that resulted from the ML analysis of the a. combined plastid and b. the ITS matrices, the selected morphological traits (the perianth morphology and the ornamentation of sporoderm) were optimized in the most parsimonious way, as implemented in Mesquite v. 3.01 (Maddison & Maddison, 2011) treating all character states as “unordered” (reviewed and summarized in Kitching et al. (1998) and Maddison & Maddison (2011)).

### Scanning electron microscopy (SEM) and light microscopy (LM)

Dry material and pollen samples were placed onto aluminum stubs, coated with gold or an alloy of platinum and palladium using a JFC-1100E sputter coater and studied under scanning electronic microscopes, Camscan-S2 and JEOL JSM-6380LA at 15–20 kV. SEM investigation was performed in the Laboratory of Electron Microscopy of M.V. Lomonosov Moscow State University, Faculty of Biology. Describing the achene surface, we followed the terminology of Ronse De Craene & Akeroyd (1988), Barthlott (1981) and Barthlott (1984). Palynological traits were described as suggested by Punt et al. (2007) and Hesse et al. (2009).

LM-images were made with the stereoscopic microscope Stemi 2000–C Carl Zeiss (Zeiss, Oberkochen, Germany) using the camera Axiocam-MR and programm AxioVision V. 4.8 free edition.

### Journal nomenclatural statement

The electronic version of this article in Portable Document Format (PDF) will represent a published work according to the International Code of Nomenclature for algae, fungi, and plants (ICN), and hence the new names contained in the electronic version are effectively published under that Code from the electronic edition alone. In addition, new names proposed in this work have been issued with identifiers by IPNI, and will eventually be made available to the Global Names Index. The IPNI-LSIDs can be resolved and the associated information viewed through any standard web browser by appending the LSID contained in this publication to the prefix “http://ipni.org/.” The online version of this work is archived and available from the following digital repositories: PeerJ, PubMed Central, and CLOCKSS.

## Results

### ITS phylogeny

The results of ML and BI of the ITS matrix of Polygoneae (Fig. 1), with *Knorringia* taken as an outgroup, show that the members of the tribe form a highly supported clade (0.99/1.0). The RFM clade (0.80/0.99) includes the genera *Reynoutria, Muehlenbeckia* and *Fallopia*. As sampled, *Reynoutria* and *Muehlenbeckia* appeared as monophyletic (0.99/1.00 and 0.91/0.99), and *Fallopia* as polyphyletic, with *F. baldshuanica* grouped with *Reynoutria*, and *F. convolvulus* and *F. dumetorum* grouped together (0.85/−). Clade *Duma* emerged as monophyletic (0.99/1.0).

The clade *Polygonum* s.l. includes: 1) a moderately supported (0.84/0.95) subclade (*Polygonella* (1.0/1.0) + *Polygonum* section *Duravia*), that contains North American taxa; 2) a highly supported (0.98/0.99) subclade including part of the *Polygonum* section *Polygonum* (North Eurasia and North America) (1.0/1.0), and part of the *P.* section *Polygonum* from Central Asia (0.98/1.0), with *P. molliiforme* and *P. bornmuelleri* (*Polygonum* section *Pseudomollia)* from Central Asia included in the latter.

Well-supported clade *Polygonum* sect. *Spinescentia* (*=Atraphaxis* sect. *Polygonoides,* Tavakkoli et al., 2015) (0.81/0.90) is recognized as a sister of *Atraphaxis* s.str. So, as sampled, *Polygonum* appeared to be polyphyletic.

*Atraphaxis* also appeared as polyphyletic. The clade corresponding to *Atraphaxis* s.str. is well supported (0.96/0.99). However, *A. ovczinnikovii* from Pamir (two accessions) was recognized as an immediate sister to the clade (*Atraphaxis* s.str. + *Polygonum* sect. *Spinescentia*), and *A. ovczinnikovii* from Tien-Shan appeared as a non-supported sister to the clade (*A. ovczinnikovii* (Pamir, two accessions) *+ Polygonum* sect. *Spinescentia + Atraphaxis* s.str.). Therefore, *A.* sect. *Ovczinnikovia* had been found as paraphyletic (Fig. 1).

The members of the sections *Atraphaxis, Physopyrum* and *Tragopyrum*, as well as the former members of *Polygonum* (*A. toktogulica, A. tortuosa, A. atraphaxiformis*, and *A. ariana*) are intermixed within the clade *Atraphaxis* s.str.

### Combined chloroplast phylogeny

In general, the results of the ML and BI analyses of the combined chloroplast data matrix are similar to the results of phylogenetic analyses of the ITS matrix (Fig. 2).

Tribe Polygoneae forms a highly supported clade (0.93/0.99). The RFM clade (*Reynoutria + Fallopia* + *Muehlenbeckia*) is also highly supported (0.92/0.99). *Duma, Polygonum, Polygonella,* and *Atraphaxis* are grouped in a strongly supported clade (0.99/1.0) sister to the RFM clade. *Duma* is monophyletic (0.99/1.0) and combined with *Polygonum* s.l. (0.95/1.0).

*Polygonum* s.l. is widely polyphyletic. Monophyletic *Polygonella* appeared as a presumbable sister (0.83 ML aLRT) of the well-supported *Polygonum* s.str. (0.83/0.97), but the members of the *Polygonum* section *Duravia* are not in the analyses.

Within *Polygonum* s.str. clade, the Eurasian species of the *Polygonum* sect. *Polygonum* (*P. aviculare, P. boreale, P. arenarium, P. luzuloides*), plus *P. plebeium* compose one subclade (1.0/1.0), and the species of *P.* sect. *Polygonum* from SW and Central Asia (*P. paronychioides, P. thymifolium, P. alpestre, P. cognatum*), and *P. molliiforme* from *P.* sect. *Pseudomollia,* enter another subclade (0.91/–). The members of *Polygonum* sect. *Spinescentia* again formed a group sister to *Atraphaxis* s.str.

The majority of the species from *Polygonum* sect. *Spinescentia* (*P. aridum, P. dumosum, P. salicornioides, P. khajeh-jamali, P. spinosum*) formed a highly supported clade (0.98/1.0) that appeared as a sister of well-supported *Atraphaxis* s.str. clade (Fig. 2).

Tavakkoli et al. (2015) reported the conflicting phylogenetic placement of the rarest and poorly known *Polygonum botuliforme*, confirmed in own analyses (Figs. 1 and 2). This issue requires future reappraisals and at the moment is excluded from future discussion.

Within *Atraphaxis* s.str., the taxa with equal-sized segments do not form a separate group. The members of *A.* sect. *Atraphaxis* (*A. spinosa, A. replicata, A. fischeri, A. karataviensis*) form a subclade (0.90/0.98), *A. teretifolia* from *A.* sect. *Physopyrum* is presumably grouped with *A. frutescens* (*A*. sect. *Tragopyrum*), and *A. toktogulica*.

*Atraphaxis* s.l. appeared as polyphyletic: two accessions *A. ovczinnikovii* from Pamir (both grouped together with a high level of support) and the single accession from Tien Shan (appeared as a strongly supported sister to the Pamirian subclade) form a highly supported clade (0.95/1.00), sister to the well supported (*Polygonum* sect. *Spinescentia +Atraphaxis* s.str.) clade (0.99/0.99).

Stressing the remarkable phylogenetic placement of *A.* sect. *Ovczinnikovia*, that emerged far away from the rest of the *Atraphaxis* s.str., both ML and BI analyses of the combined plastid matrix argue for the strong monophyly of the sect. *Ovczinnikovia*, that appeared to be paraphyletic as a result of the phylogenetic analyses of the ITS data set (see above.)

### Morphology of *Atraphaxis ovczinnikovii, Polygonum* sect. *Spinescentia*, and *Atraphaxis* s.str.

We compared main morphological characteristics of the species that had been traditionally placed in *Atraphaxis* s.str. and had been included recently. The latters include the Pamirian and Tien-Shanian accessions *Atraphaxis ovczinnikovii,* remarkable by their distant positions from *Atraphaxis* s.str. in phylogenetic reconstructions, *Polygonum* sect. *Spinescentia*, which is sister to *Atraphaxis* s.str., and *A. toktogulica, A. tortuosa, A. atraphaxiformis*, and *A. ariana,* which are nested within *Atraphaxis* s.str. clade. Morphological characteristics of the taxa are summarized in Tables 1 and S4.

### Life history and general architecture of shoot system

The taxa under study demonstrate some distinctions in the structure of vegetative organs and inflorescences. Both *Atraphaxis* *ovczinnikovii* from Pamir (Tajikistan) and *A. ovczinnikovii* from Tien Shan (Kyrgyzstan) can be described as divaricately branched dwarf shrubs 10–30 cm tall, with elongated leafy annual shoots, and generative shoots terminated by frondulose thyrses with 3–7 axillary cymes of 1–2 flowers (Figs. S1 and S2). The second-year shoots are covered with a gray fibrously disintegrated bark. The plants from Tian Shan have foxy-colored ribbed annual shoots, the plants from Pamir have greenish-gray rounded annual shoots, and in both taxa they are shortly puberulent.

All accessions of *A. ovczinnikovii* are fairly distinct in habit from the members of *Polygonum* section *Spinescentia*, which are dwarf, cushion-shaped caespitose undershrubs, shrubs, or perennials, some with prickly shoots and branchlets (Jaubert & Spach, 1844–1846; Boissier, 1846; Boissier, 1879; Gross, 1913; Mozaffarian, 1988; Mozaffarian, 2012; Khosravi & Poormahdi, 2008; Tavakkoli et al., 2015). *Polygonum salicornioides* is a cushion-shaped dwarf undershrub with the shoots not lignified and less prickly (Fig. S3). These taxa have frondulose or bracteose thyrses.

The members of the genus *Atraphaxis* are dwarf or tall shrubs, 30–150 cm tall, rarely undershrubs with leafy elongated annual shoots and short axillary branchlets (Figs. S4–S13). *Atraphaxis ariana* and *A. toktogulica* are undershrubs, like *A. frutescens*, with a woody manyheaded taproot (caudex) and numerous elongated generative shoots dying off almost to the base (Figs. S4 and S5). *Atraphaxis atraphaxiformis* and *A. tortuosa* are shrubs, like many other *Atraphaxis* species, with intensively branched elongated shoots terminated by bracteose thyrses (Figs. S6–S13 and 13A).

The majority of species have light-green or creamy annual shoots. The second-year shoots are usually covered with a light-gray bark, rarely they are yellowish-gray (*A. laetevirens* and *A. caucasica*), or foxy-brown (*A. pungens* and *A. muschketowi*). The annual shoots are densely covered with 0.2 mm long papillae, or glabrous.

Some species have terminal elongated thyrses, which are simple (*A. avenia*), or with paracladia forming a raceme of thyrses (*A. ariana, A. toktogulica*, *A. virgata, A. frutescens, A. bracteata*). These thyrses include 10–20 cymes of 2–6 flowers.

In many species the thyrses, terminating both leading axes and axillary branchlets, are compact, with congested cymes of flowers (*A. seravschanica*, *A. kopetdaghensis, A. laetevirens, A. muschketowi, A. caucasica, A. billardierei*, *A. tournefortii*, *A. pyrifolia*, *A. spinosa*) (Figs. S8–S13). *Atraphaxis pyrifolia*, *A. pungens* have lignified prickly elongated shoots and branchlets, so their compact thyrses are in lateral position at the second-year branchlets (Fig. S11). *Atraphaxis spinosa* has racemes of short thyrses terminating annual shoots, and short axillary thyrses at the first- and second-year shoots (Figs. S12 and S13). The axillary thyrses of *A. spinosa* include 5–6 cymes of 1–(2) flowers per a cyme, so their thyrses resemble the axillary fascicles of 5–6 flowers (Fig. 14A).

### Leaf blades

**Figure 3:**
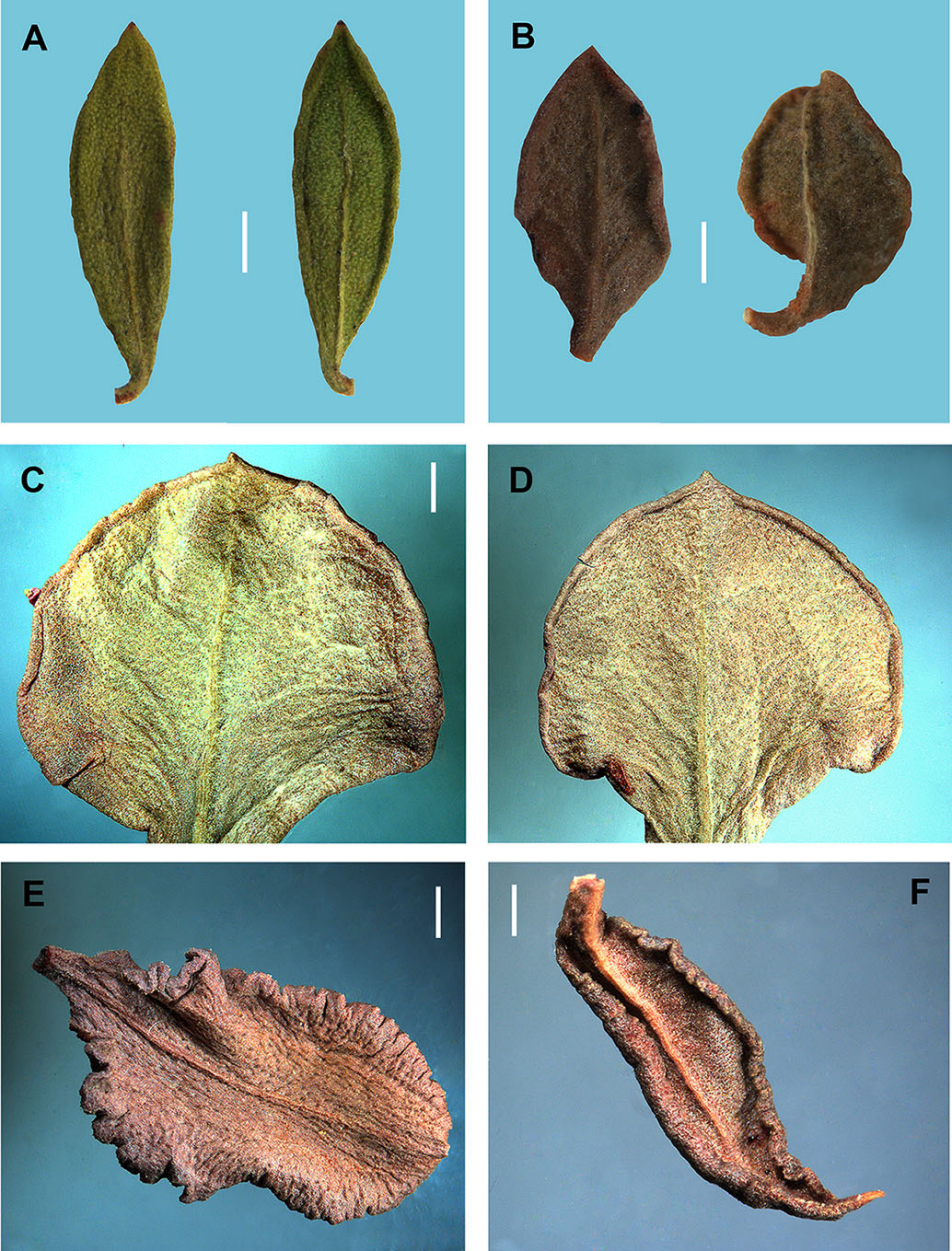
Leaf blades of *Atraphaxis* adaxially and abaxially. (A) *A. ovczinnikovii* (=*Bactria lazkovii*) from Tien Shan. (B) *A. ovczinnikovii* (=*Bactria ovczinnikovii*) from Pamir. (C–D) *A. atraphaxiformis.* (E) *A. tortuosa.* (F) *A. toktogulica*. Scale bar for (A–F) = 1 mm. Images: O. Yurtseva.

*Atraphaxis* *ovczinnikovii* from Tien Shan has oblong-elliptical or lanceolate leaf blades, which are acuminate, gradually narrowed to a petiole 1–2 mm long or almost sessile, joined with articulation, bright green, coriaceous, revolute at margin, shortly puberulent abaxially only along midvien, without laterals (Fig. 3A). *Atraphaxis* *ovczinnikovii* from Pamir has broadly ovate or rhomboid-elliptical leaf blades, which are shortly acuminate, suddenly narrowed to a petiole 1–2 mm long, or sessile, grayish-green, thick, slightly undulate and revolute at margin, shortly densely puberulent adaxially and abaxially, with laterals below (Fig. 3B).

Leaf blades of *Polygonum* sect. *Spinescentia* were described as linear-elliptical, or linear-lanceolate, slightly revolute at the margin (*P. aridum, P. dumosum, P. spinosum*); ovate to oblong-lanceolate and fleshy (*P. salicornioides, P. khajeh-jamali*), in all the species shortly puberulent (Jaubert & Spach, 1844–1846; Boissier, 1846; Boissier, 1879; Gross, 1913). *Polygonum botuliforme* differs by sausage-shaped, glabrous leaf blades (Mozaffarian, 1988; Mozaffarian, 2012; Tavakkoli et al., 2015).

*Atraphaxis* s.str. has diverse leaf blades, varying in shape from rotundate, broadly-ovate, or broadly-elliptical to spatulate, oblong-elliptical and linear-lanceolate (Figs. 3C–3F, 13A, 14B and S4–S13). Leaf blades are usually thick, leathery, flat or slightly revolute, often finely crenulate or undulate at margin, glabrous or shortly puberulent. *Atraphaxis teretifolia* from the section *Physopyrum* has linear terete shortly puberulent leaf blades (Figs. 15B and 15E).

### The ochreae in thyrses and vegetative shoots

**Figure 4:**
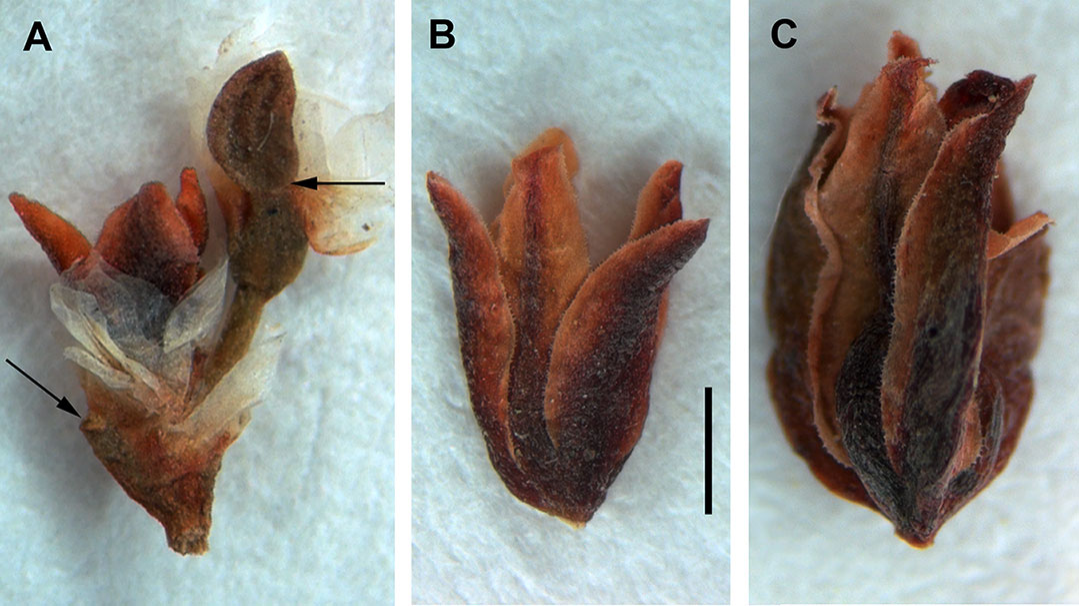
Fragment of frondulose thyrse and perianth of *Atraphaxis ovczinnikovii* (=*Bactria ovczinnikovii*) from Pamir. (A) Fragment of frondulose thyrse with two axillary cymes of 1(2) flowers, ochreae and ocreolae. Arrows point to the places of leaf blade articulation. (B) Perianth of young flower divided to 7/10. (C) Perianth of premature flower divided to 9/10, with an achene inside. Scale bar for (A–C) = 1 mm. Images: O. Yurtseva.

*Atraphaxis ovczinnikovii* from Pamir in thyrses has the ochreae 2–4 mm long, broadly ovate, cup-form inflated under the petiole, at base greenish-brown, herbaceous, densely shortly puberulent, above membranous, semitransparent, without visible veins, entire, bidentate (if a leaf blade is reduced fully), or cleft in two shortly acuminate lacinulae without veins at both sides of reduced leaf blade (Fig. 4A). In vegetative shoots the ochreae are similar in size and consistence, but lanceolate-tubulate, later cleft in two lanceolate lacinulae.

*Atraphaxis ovczinnikovii* from Tien Shan has similar ochreae, but brownish at base, transparent above, puberulent along the keel and later split in two lanceolate lacinulae, each with a single reddish vein.

The ochreae of *Polygonum* sect. *Spinescentia* were described as tubulate, 3–5 to 7–10 mm long, membranous, transparent, without veins (*P. spinosum, P. dumosum*), witn two veins (*P. salicornioides*), or 5–6 veins (*P. aridum*) (Jaubert & Spach, 1844–1846; Boissier, 1846; Boissier, 1879; Gross, 1913). According to Tavakkoli et al. (2015), the ochreae are 2–4 mm long, tubulate, truncate, shortly bidentate, and similar to those of *Atraphaxis*.

However, in vegetative shoots of *A. ariana, A. toktogulica, A. atraphaxiformis, A. tortuosa* the ochreae are 7–10 mm long, tubulate, herbaceous and puberulent at the base, membranous and transparent above, with two long linear-lanceolate aristate lacinulae at both sides of the leaf blade, each with a single vein, and a short finely serrate-incised middle part at the side opposite to the leaf blade. Other species (*A. kopetdaghensis, A. avenia*, *A. seravschanica, A. frutescens, A. pungens, A. bracteata, A. virgata*) have the ochreae similar in morphology and consistence, but 3–7 mm long, oblique-tubulate. Only *A. spinosa* has the ochreae 2–3 mm long, lacerate in two linear-lanceolate lacinulae with hardly visible veins, and a short middle part.

In thyrses, the majority *Atraphaxis* species have the ochreae 2–7 mm long, oblique-funnel form, truncate, almost fully membranous and transparent, with a leaf blade reduced to a small herbaceous outgrowth, or a narrow keel (*A. atraphaxiformis, A. tortuosa, A. toktogulica, A. frutescens, A. virgata, A. avenia, A. seravschanica, A. ariana, A. badhysi,* Figs. 8A–8C, 10A, 10F, 11A, 12A, 12B, 15A and 15B), the latter not visible in some species (*A. spinosa, A. pungens, A. laetevirens, A. muschketowi, A. pyrifolia*). *Atraphaxis teretifolia* has the ochreae 5–7 mm long, tubulate, funnel-form, with two long aristate linear-lanceolate lacinulae at both sides of the leaf blade, each with a single vein (Figs. 15A and 15B). *Atraphaxis atraphaxiformis* and some other species have puberulent ochreae and the axes of thyrses (Figs. 8B and 8D). Each flower in a monochasium is surrounded from the back by a membranous bikeeled prophyll formed by two connate stipulae.

### Perianth morphology

A total of 25 characteristics are listed in Table 1, however, the perianth shape, the perianth partition, and the segment consistence are the most prominent for the discrimination of Tien-Shanian and Pamirian accessions *Atraphaxis ovczinnikovii* from *Polygonum* sect. *Spinescentia* and the rest *Atraphaxis* s.str. within *Polygoneae* (Table S5). Characteristics of the perianth, the perianth and achene size of the taxa under study are given in Table S4. Some brief notes on the perianth shape are still necessary.

**Figure 5:**
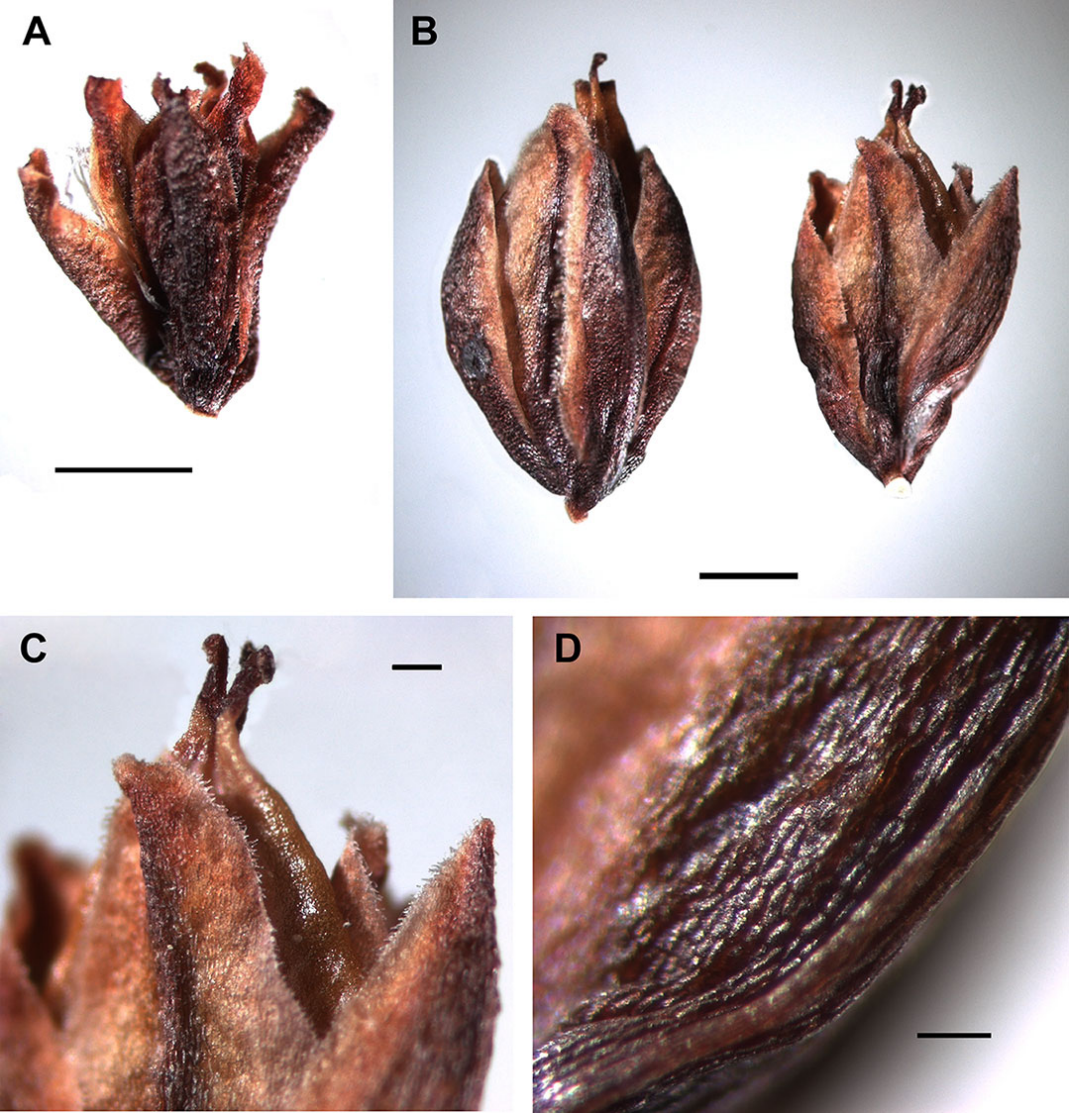
Flowers of *Atraphaxis ovczinnikovii* (=*Bactria ovczinnikovii*) from Pamir. (A) Perianth of young flower divided to 8/10. (B) Two flowers with premature achenes. (C) Fragment of the perianth with papillae at the segment edge and premature achene with three styles connate at base. (D) Base of the outer segment with a narrow keel. Scale bars for (A, B) = 1 mm, for (C, D) = 0.2 mm. Images: O. Yurtseva.

**Figure 6:**
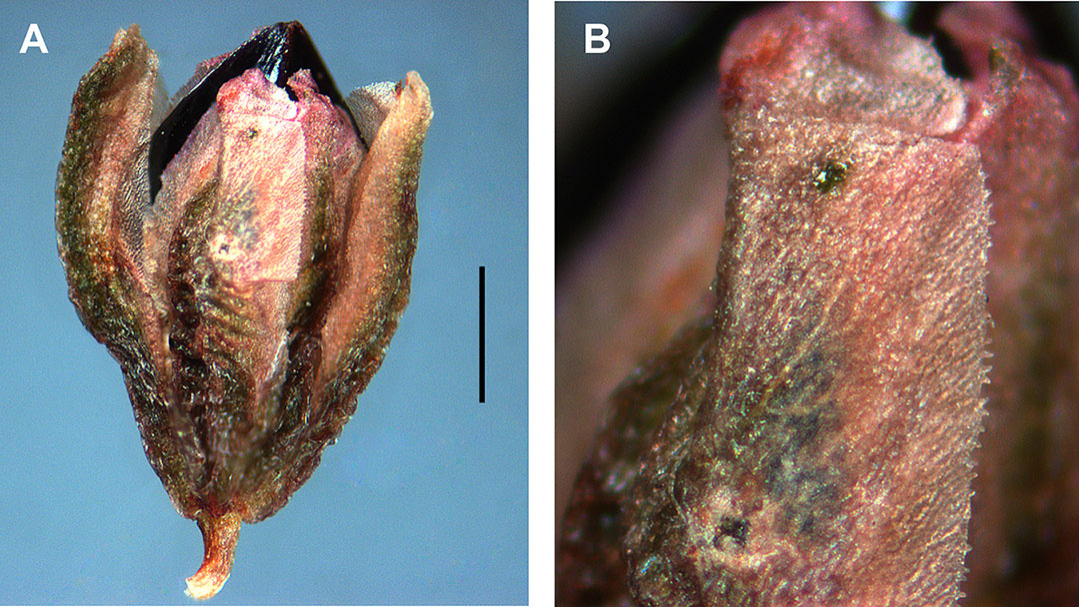
Perianth of *Atraphaxis ovczinnikovii* (=*Bactria lazkovii*) from Tien Shan. (A) Perianth surrounding the mature achene. (B) Papillate edge of the outer segment. Scale bar for (A) = 1 mm. Images: O. Yurtseva.

All accessions of *Atraphaxis ovczinnikovii* from Tien Shan and Pamir have the campanulate perianth and five equal-sized segments with stomata along the midveins and papillae at the edge of the segments (Figs. 4–6).

Pamirian accessions have the perianth 3.0–4.0 mm long (4.0–5.0 mm by fruiting), divided almost to the base (8/10) in segments lanceolate, gradually acuminate, green-purple, coriaceous, with a narrow pinkish margin. The outer segments are concave, narrowly keeled and slightly cucullate, the inner segments are slightly keeled and almost flat. The perinath tube of Pamirian plants is funnel-form, short, suddenly narrowed to a filiform base at the place of articulation with a pedicel (Figs. 4, 5 and 16A; Tables 1 and S4).

Tien Shanian accession has the perianth 2.0–2.5 mm long (2.5–3.0 mm by fruiting), divided to 4/5–5/6 in segments elliptical or broadly ovate, petalloid, herbaceous-green along the midveins, with wide semitransparent pink margin (Fig. 6; Tables 1 and S4). The outer segments are slightly acuminate, cucullate, without keel, the inner segments are obtuse and flat. The tube is cup-shaped or sacciform at base and joined to a pedicel with articulation, not narrowed to the place of articulation.

**Figure 7:**
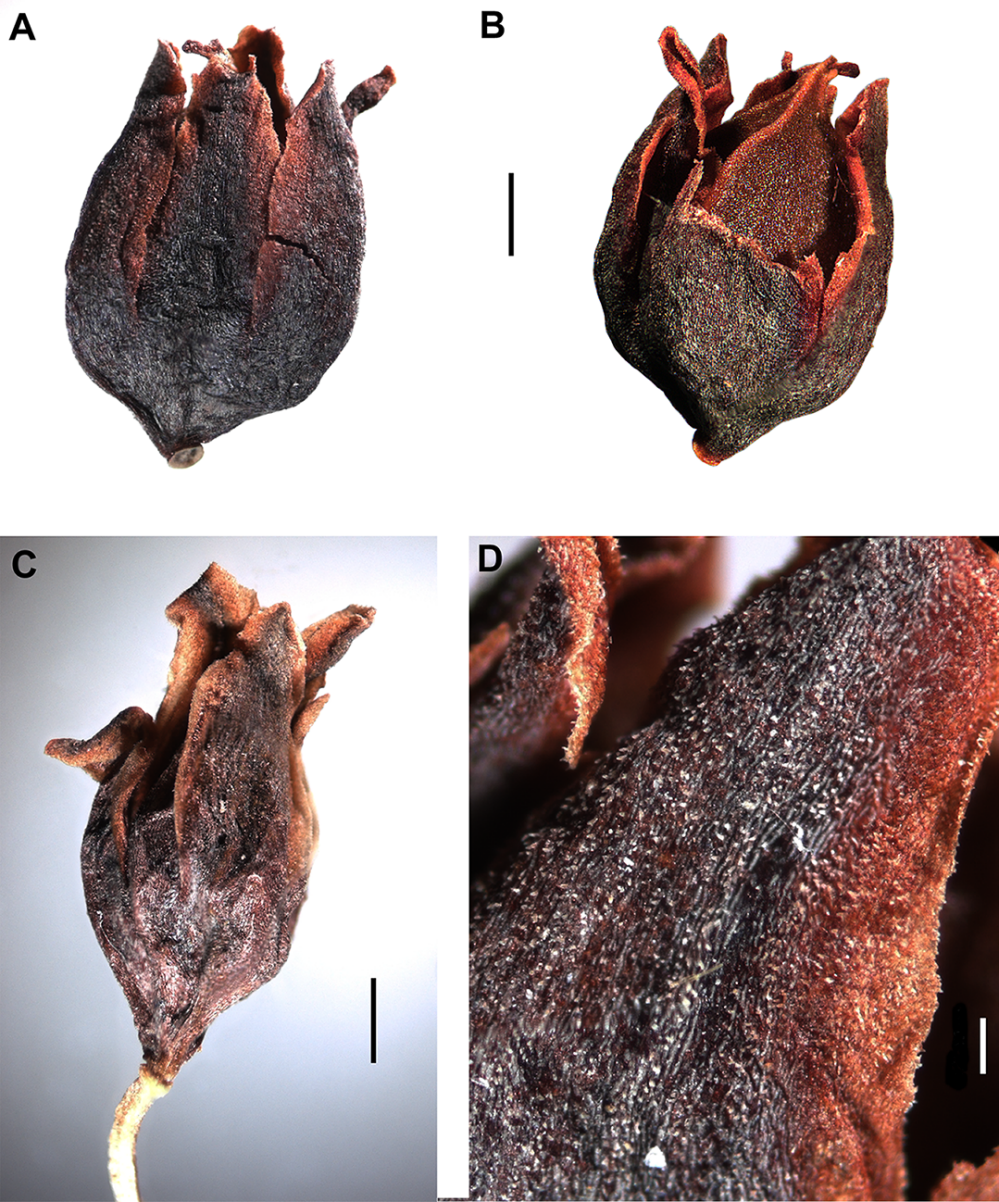
Flowers of *Polygonum salicornioides*. (A–B) Perianth in fruiting stage divided to 2/3 into five equal-sized segments surrounding the mature achene with minutely tuberculate surface. (C) Perianth of premature flower divided to 2/3. (D) Puberulent surface of perianth segments. Scale bar for (A–C) = 1 mm, for (D) = 0.2 mm. Images: O. Yurtseva.

The perianth morphology of *Polygonum* sect. *Spinescentia* is different. Remarkable *Polygonum salicornioides* has densely shortly puberulent urceolate perianth (6.0–6.5 mm long) divided to 2/3 in five equal-sized oblong-elliptical or oblong-ovate segments and funnel-form tube gradually narrowed to a place of articulation with a pedicel (Tables 1 and S4; Fig. 7). The segments are gradually acuminate or obtuse, rigid, coriaceous, purple-green, with extremely narrow membranous margin, the outer segments are slightly keeled and cucullate. By fruiting the perianth tightly surrounds the achene enclosed by the segments (Figs. 7A and 7B). The other members of this group have urceolate or campanulate perianth divided to 2/3–3/4, densely puberulent outside in all the species, except for *P. botuliforme* (Jaubert & Spach, 1844–1846; Boissier, 1846; Boissier, 1879; Gross, 1913; Mozaffarian, 1988; Mozaffarian, 2012; Tavakkoli et al., 2015).

**Figure 8:**
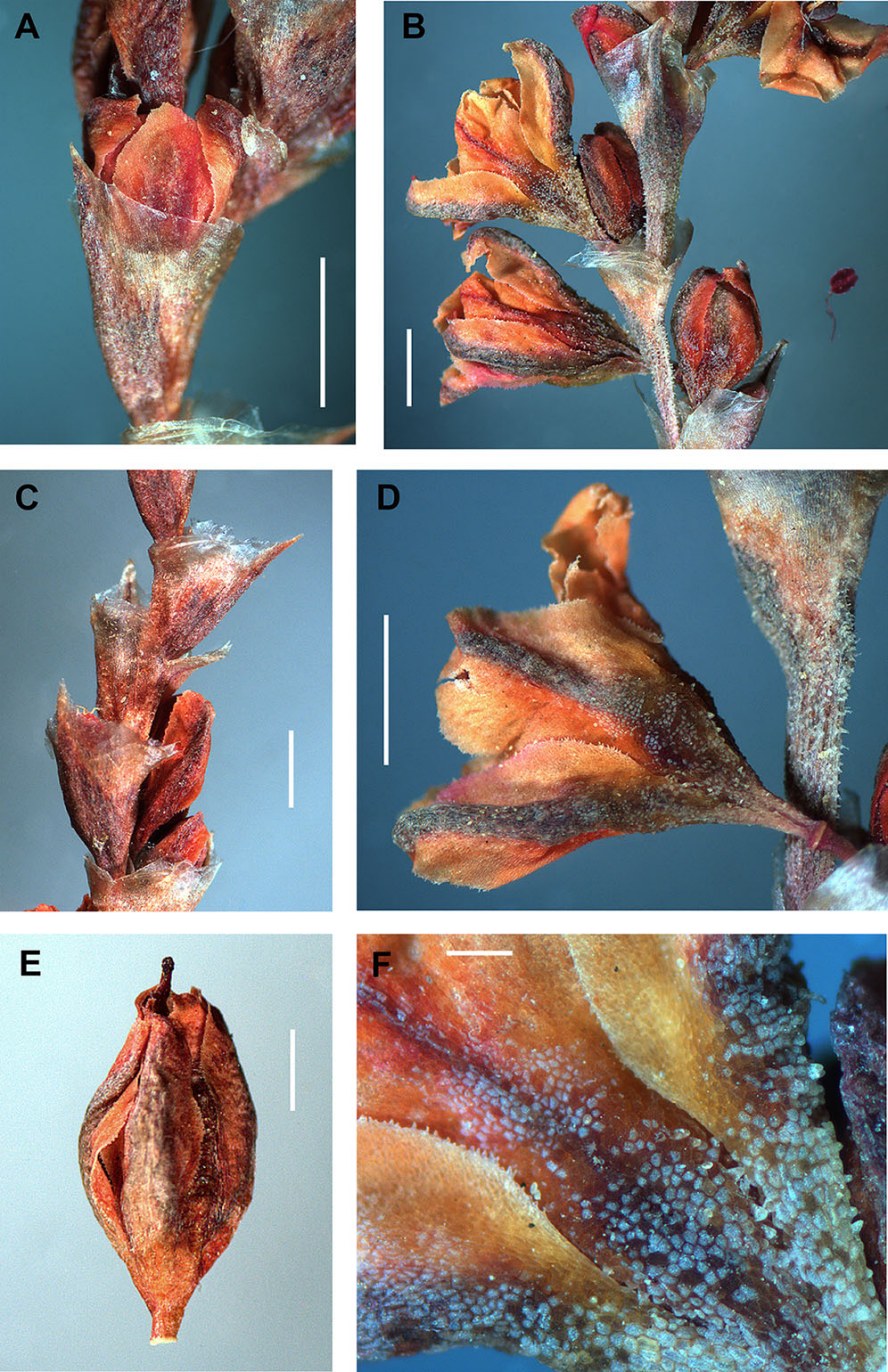
Details of the thyrse and flowers of *Atraphaxis atraphaxiformis*. (A–C) Fragments of thyrse with the cymes of 1–2(3) flowers in ochreae. (D) Perianth of blooming flower with the filiform base of tube. (E) Perianth of mature flower divided to 3/4 and including a mature achene. (F) Papillae at the perianth tube, segment edge and base. Scale bar for (A–E) = 1 mm, for (F) = 0.2 mm. Images: O. Yurtseva.

Perianth morphology in *Atraphaxis* s.str. is rather diverse. Nevertheless, all the members of the clade *Atraphaxis* s.str. have spheroidal flower buds and premature flowers with a perianth divided to 1/2–3/4 in 4–5 equal-sized petalloid white or brightly colored segments, with a funnel-form tube narrowed to a filiform basal part, joined to a pedicel with articulation (Figs. 8A, 8B, 8D, 10A, 10B, 17A–17E and 17J).

**Figure 9:**
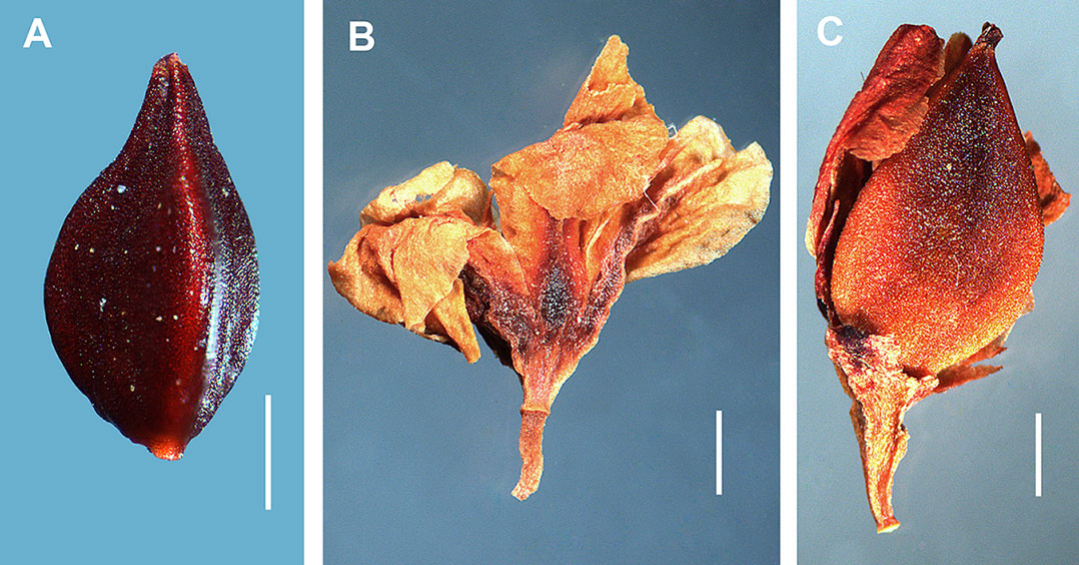
Achenes and perianths of *Atraphaxis atraphaxiformis* and *A. tortuosa*. (A) Achene of *A. atraphaxiformis* with minutely tuberculate surface. (B) Perianth of *A. tortuosa* divided to 2/3. (C) Achene of *A. tortuosa* with minutely tuberculate surface. Scale bar = 1 mm. Images: O. Yurtseva.

**Figure 10:**
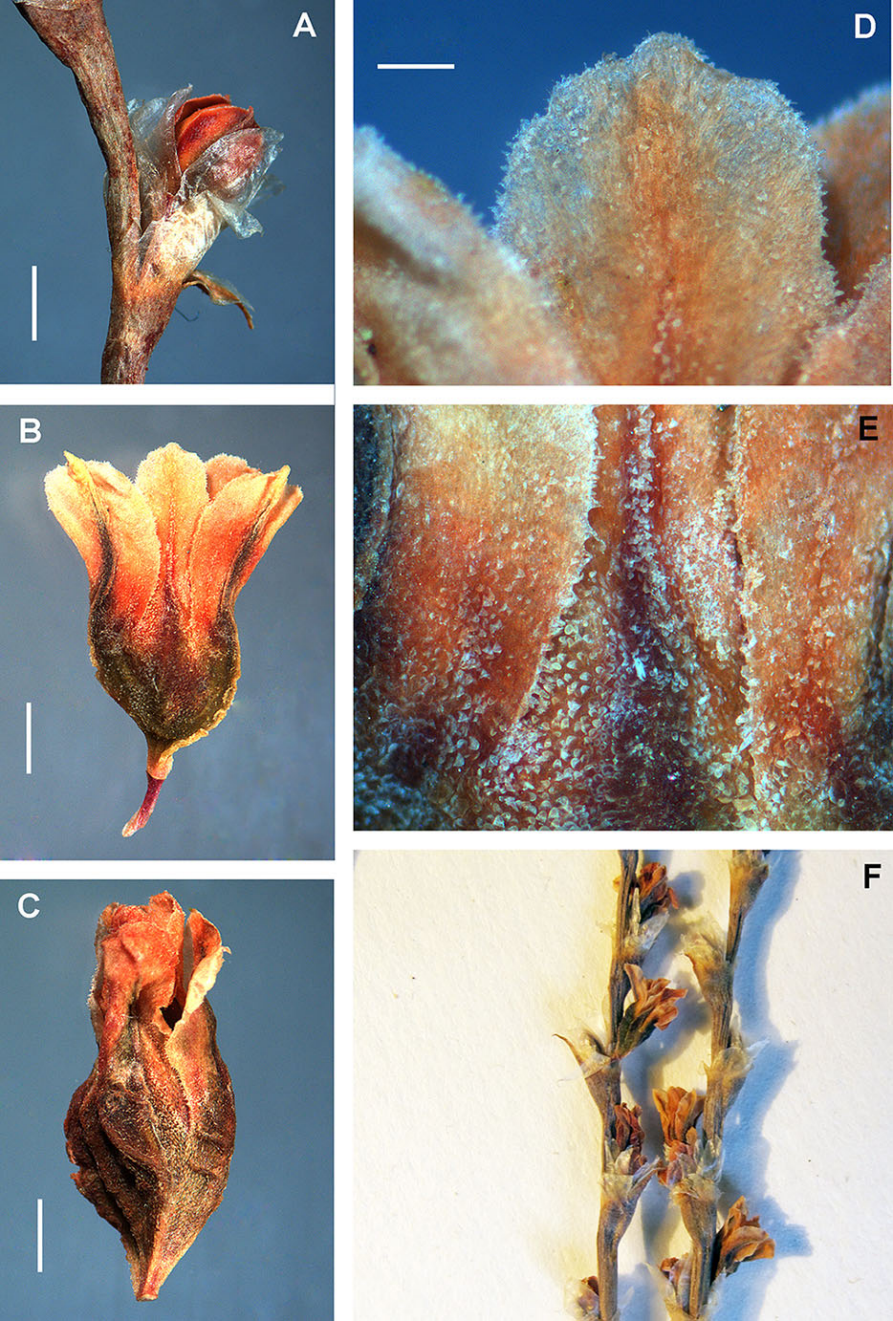
Details of the thyrse and flowers of *Atraphaxis toktogulica*. (A) Cyme with a spheroidal flower bud. (B) Perianth of flower in blossom. (C) Perianth including a mature achene. (D, E) Details of the perianth with papillae at the segment edge, segment base and tube. (F) Fragments of bracteose thyrse. Scale bar for (A–C) = 1 mm, for (D, E) = 0.2 mm. Images: O. Yurtseva.

**Figure 11:**
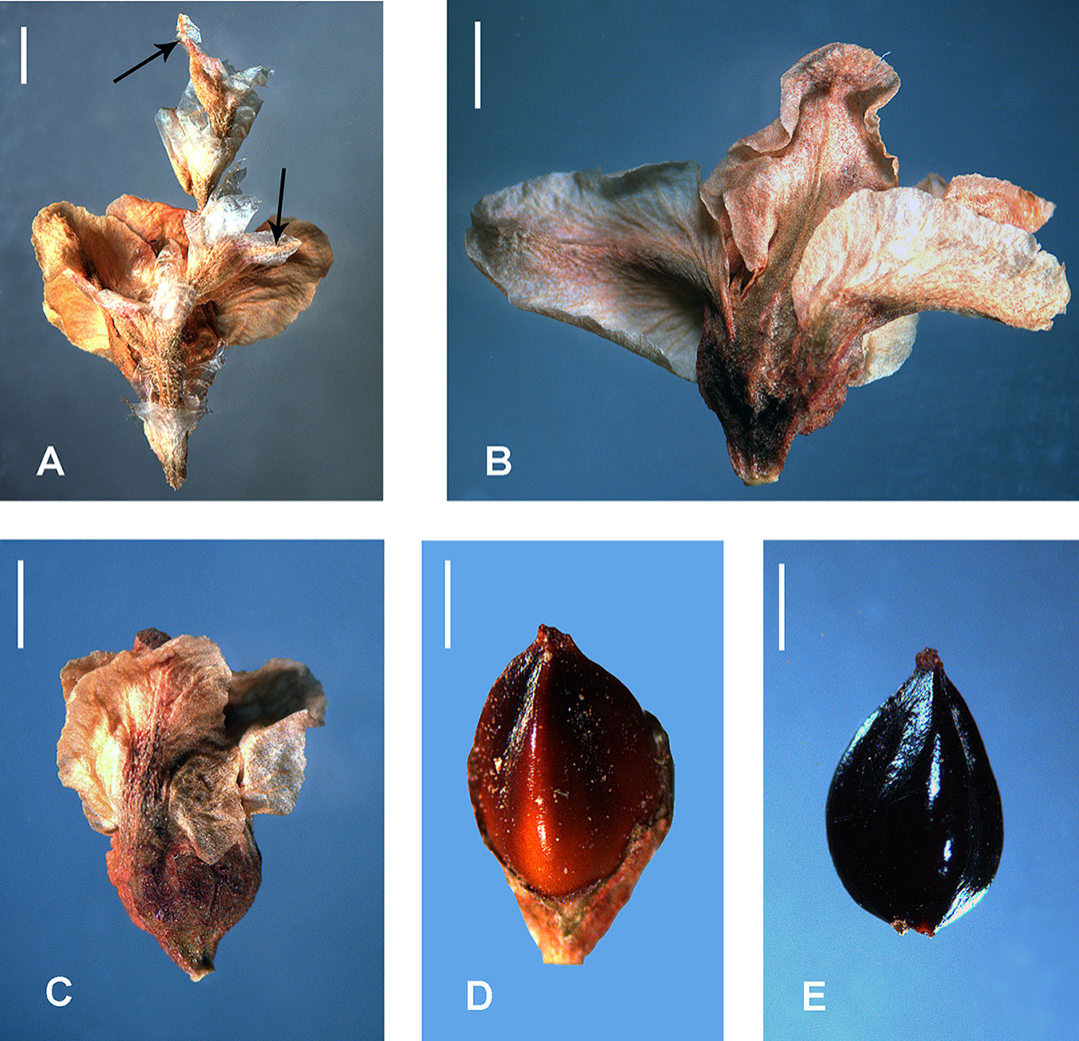
Flowers and achenes of *Atraphaxis ariana*. (A) Fragment of the thyrse with transparent ochreae provided with keels (arrows). (B, C) Perianth of blooming flower. (D–E) Premature and mature achenes. Scale bar = 1 mm. Images: O. Yurtseva.

**Figure 12:**
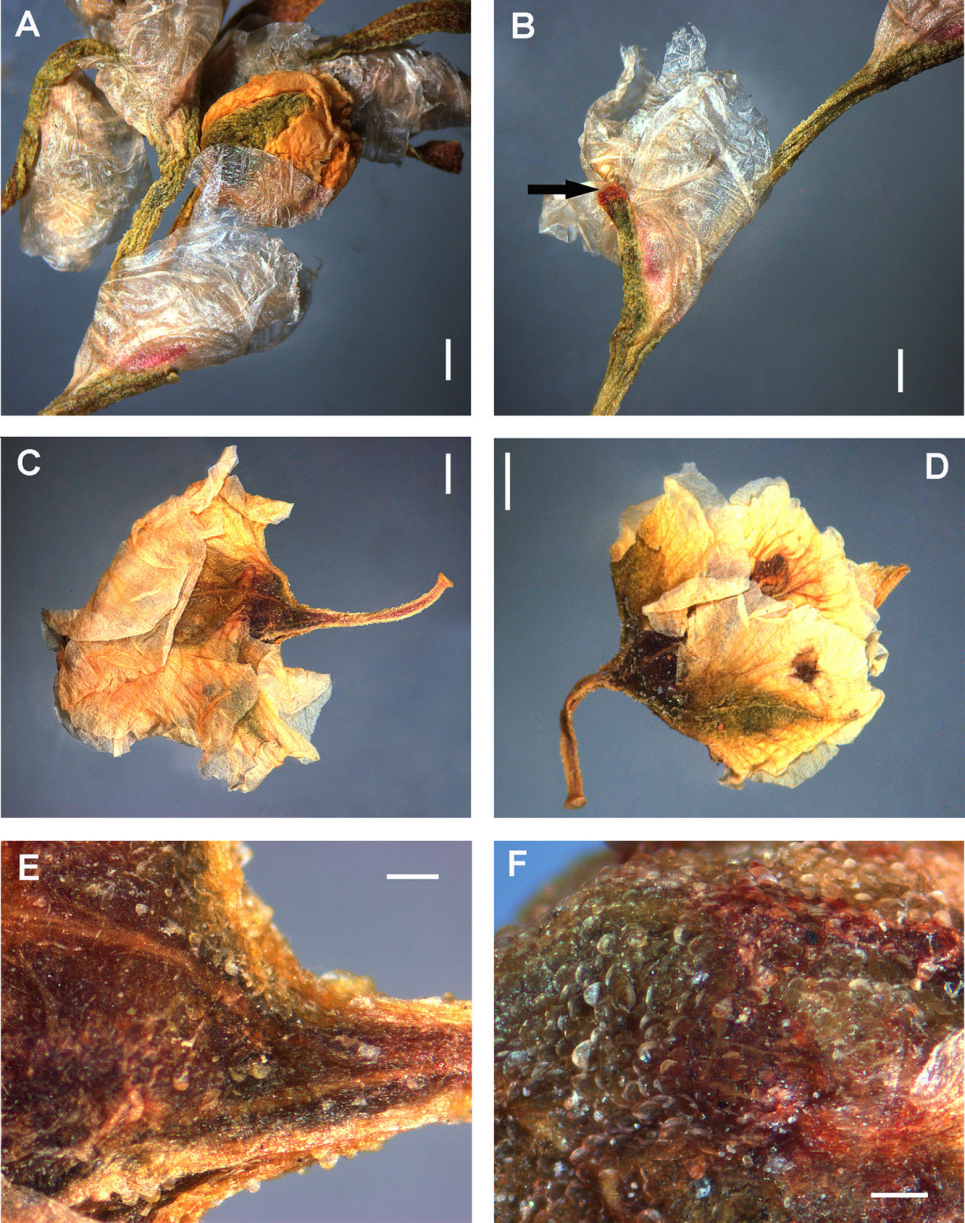
Details of the thyrse and flowers of *Atraphaxis badghysi* (A–E) and *A. ariana* (F). (A–B) Fragment of frondulose thyrse, arrow points to the place of leaf blade articulation. (C, D). Perianth with equal-sized segments and filiform tube. (E, F) Papillae at perianth tube. Scale bar for (A–D) = 1 mm, for (E–F) = 0.2 mm. Images: O. Yurtseva.

By the fruiting stage *Atraphaxis atraphaxiformis, A. tortuosa, A. toktogulica*, and *A. ariana* preserve the campanulate perianth divided to 2/3–3/4 in five equal-sized segments and rather short funnel-form tube with a filiform base not exceeding 0.5–0.7 (1.0) mm (Figs. 8C, 8E, 9B, 10C, 11B, 11C, 16D and 16F; Table S4). However, the segments are always petalloid, brightly colored or white, broadly-ovate, obtuse, flat, the outer ones slightly keeled (like in *A. ariana*); all oblong-ovate, the outer segments slightly mucronate, cucullate, and keeled in *A. atraphaxiformis* and *A. toktogulica*; oblong-ovate, obtuse and flat in *A. tortuosa* (Figs. 8–11). The upper part of the perianth tube and the segments along midveins are green and bear stomata. Papillae densely cover the perianth tube and the segment bases of *A. atraphaxiformis, A. tortuosa,* and *A. toktogulica* (Figs. 8D, 8F, 9B, 10D and 10E), and the tube of *A. ariana* (Fig. 12F), but are usually absend from the perianths of other species.

**Figure 13:**
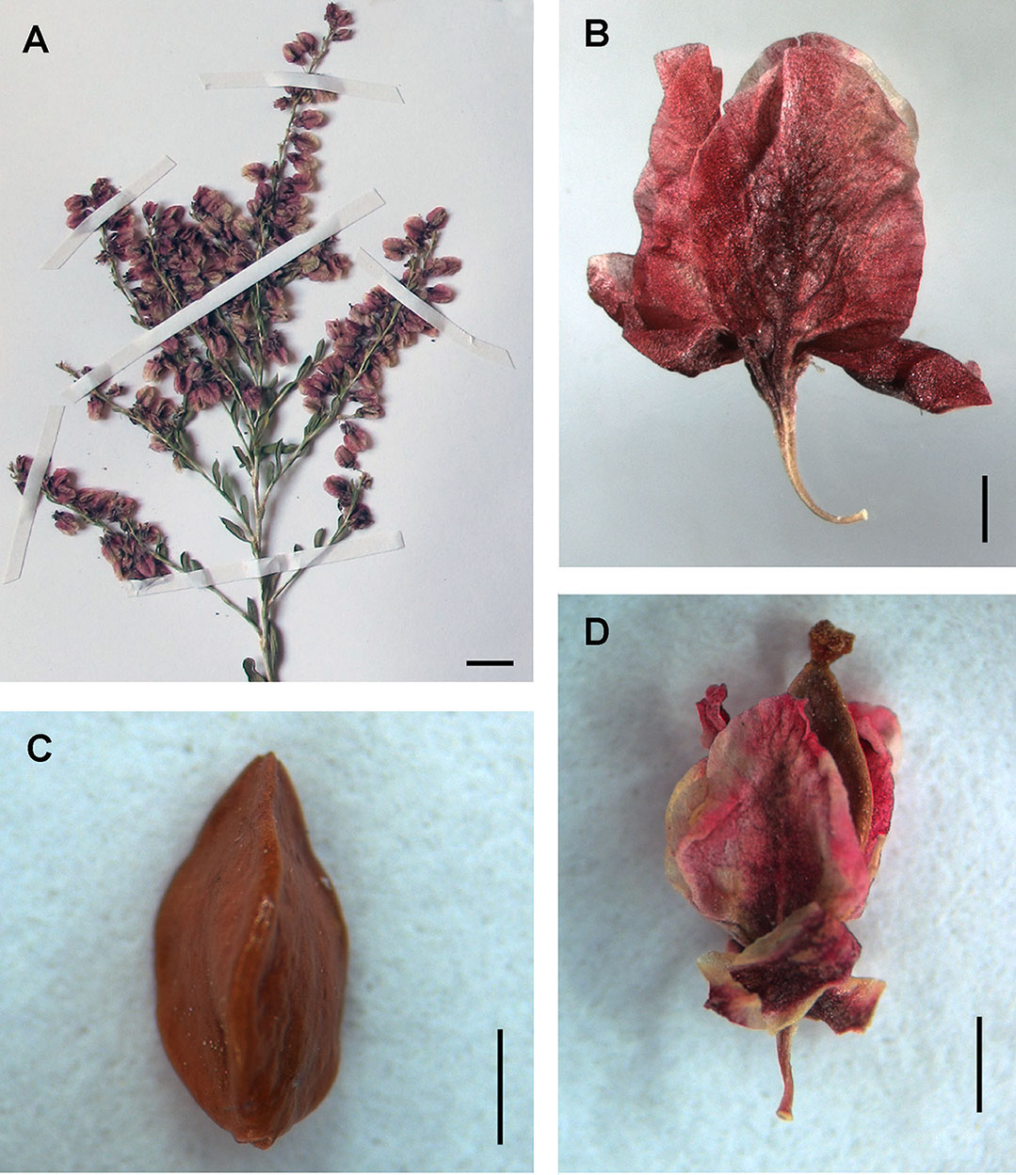
Inflorescense, flowers and achene of *Atraphaxis virgata* (*A.* section *Tragopyrum*). (A) Raceme of thyrses. (B) Perianth with filiform tube, three accrescent inner segments and two outer segments. (C) Mature achene. (D) Perianth including the mature achene with three styles. Scale bar for (A) = 1 cm, for (B–D) = 1 mm. Images: O. Yurtseva.

**Figure 14:**
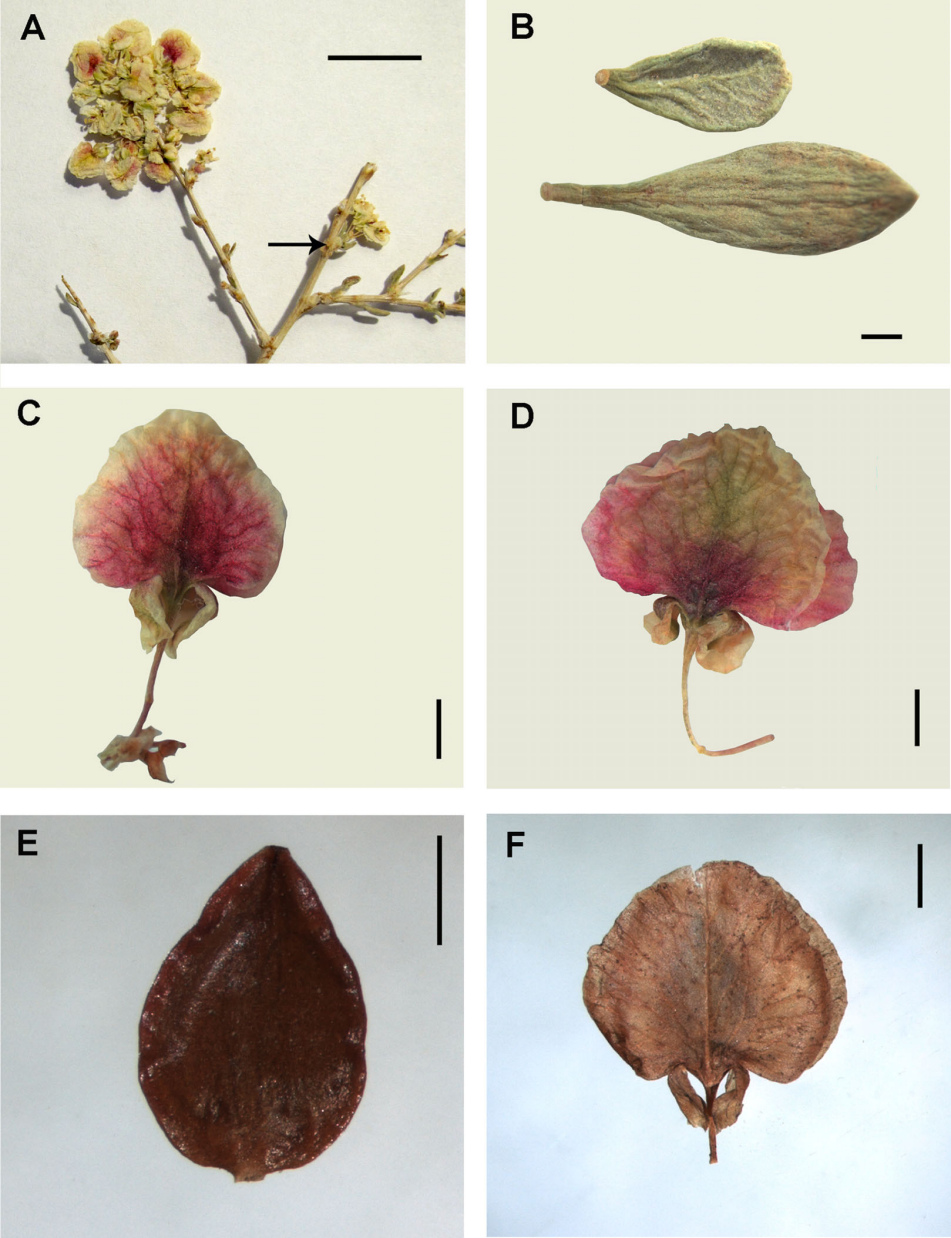
Inflorescense of *Atraphaxis spinosa* from Armenia (A–C) and *A. fischeri* from the Low Volga (D–F) (*A.* section *Atraphaxis*). (A) Terminal raceme of thyrses and two axillary thyrses (arrow) with 4–5 cymes of 1 flower. (B) Leaf blades. (C–D) Perianth with the filiform tube joined to the pedicel, two outer and two inner segments. (E) Lenticular achene. (F) Perianth enclosing a mature achene. Scale bar for (A) = 1 cm, for (B–F) = 1 mm. Images: O. Yurtseva.

The perianths of the majority of *Atraphaxis* species undergo transformation by the fruiting stage, the inner segments becoming much larger, then the outer segments, and enclosing the achene. The inner segments are broadly-elliptical, broadly ovate, orbiculate or reniform. The filiform basal part of the tube is up to 2.0–7.0 mm, the expanded upper part of tube is either wedge- or cup-shaped (Figs. 13B, 13D, 14C, 14D and 14F; Table S4).

*Atraphaxis badghysi, A. bracteata, A. aucherii, A. angustifolia, A. grandiflora* demonstrate a transitional perianth type divided to 1/2–2/3 into five subequal rotundate, rhomboid-elliptical or broadly-elliptical segments, and the tube with a filiform base 1.5–3.0 mm long (Figs. 12C, 12D and 16G; Table S4). The tube of *A. badghysi* bears papillae (Fig. 12E).

**Figure 15:**
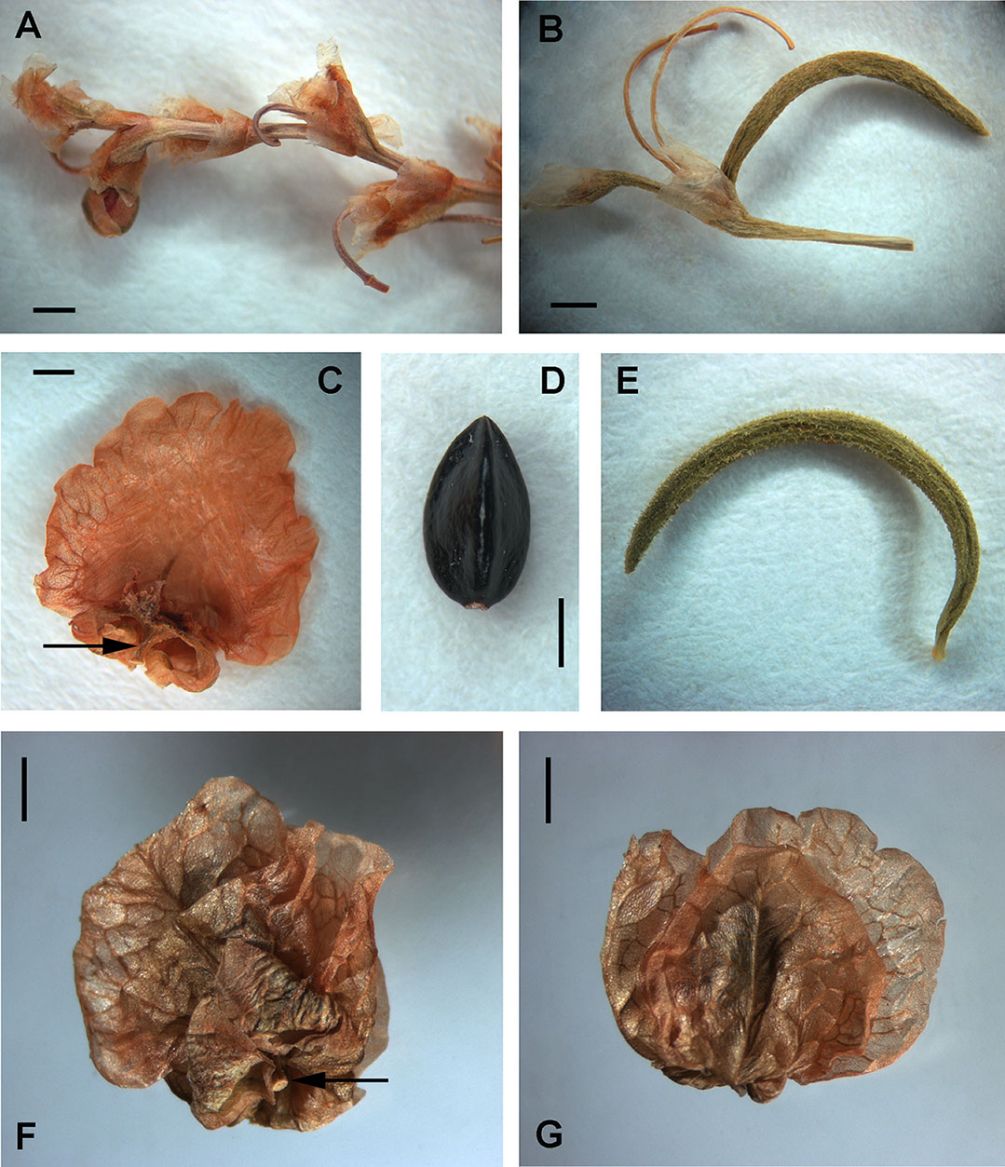
Fragment of the thyrse, flowers and achene of *Atraphaxis teretifolia* (*A.* section *Physopyrum*). (A) Fragment of the thyrse (leaf blades are fallen). (B) Axillary cyme with a leaf blade and two long pedicels. (C) Fragment of the perianth with two outer and one accrescent inner segment, the arrow points at short filiform tube. (D) Achene. (E) Linear terete leaf blade covered with papillae. (F–G) Perianth with three large concave inner segments, two small outer segments and short filiform tube (arrow). Scale bar = 1 mm. Images: O. Yurtseva.

The majority of the species in *Atraphaxis* sect. *Tragopyrum* have three inner segments greatly accrescent by fruiting, and two small segments reflected to a pedicel (Figs. 13B and 13D). The members of the section *Atraphaxis* have the perianth with two reniform inner segments accrescent by fruiting and two small outer segments (Figs. 14C, 14D and 14F). *Atraphaxis teretifolia* (sect. *Physopyrum*) has the perianth with three inner segments orbiculate, concave, spherically surrounding the achene, and two outer segments small and reflected to a pedicel (Figs. 15C, 15F, 15G and 17G; Table S4). The filiform basal part of the tube, 0.5–0.7 mm long, joins to a pedicel 5–6 mm long.

Depending on relative size of outer and inner segments, the shape of the segments (oblong, ovate, rounded, or reniform, flat, or concave), the edge of the segments (entire, undulate, crenulate, smooth or papillate), flower merosity (the number of outer and inner segments), the length of the filiform basal part of the tube, the shape of the upper part of the perianth tube (wedge-shaped, funnel-form, or cup-shaped), several perianth types are distinguishable within *Atraphaxis* s.str. (Table S4). However, all of the species of *Atraphaxis* are sharing the thin petalloid segments that are expanded at the top, white or brightly colored.

The analysis of the perianth morphology of Polygoneae (Tables 1 and S5; Fig. 20) shows that the ancestral character state of the clade (*Atraphaxis ovczinnikovii* + *Polygonum* sect. *Spinescentia* + *Atraphaxis* s.str.) is a campanulate perianth with equal-sized segments and a short tube. More specialized perianth with accrescent inner segments and a long filiform tube is a synapomorphy of *Atraphaxis* s.str.

*Atraphaxis toktogulica, A. ariana, A. atraphaxiformis*, and *A. tortuosa* (all with equal-sized perianth segments) do not form a separate subclade within *Atraphaxis* s.str., however, *A. angustifolia* and *A. grandiflora, A. angustifolia* and *A. aucheri,* all with subequal segments, are grouped together (Figs. 1 and 2). Within *Atraphaxis* s.str., the members of the sections *Atraphaxis*, *Physopyrum* and *Tragopyrum* with accescent inner segments are intermixed with the species with equal-sized segments (Fig. 20). In other words, the specialized perianth with accrescent inner segments evolved homoplastically.

### Achene morphology

Achene sizes are given in Table S4. *Atraphaxis ovczinnikovii* from Pamir has ovoid, gradually acuminate larger achenes (4.0–5.0 × 2.5–2.8 mm) with three styles connate at the base and forming a triquetrous stub at the top of the achene, each inflated at the base and filiform under the capitate stigma. The achenes with distinct ribs and equal concave faces are light-brown, smooth and glossy, either slightly exserted, or enclosed by the perianth (Fig. 5C).

**Figure 16:**
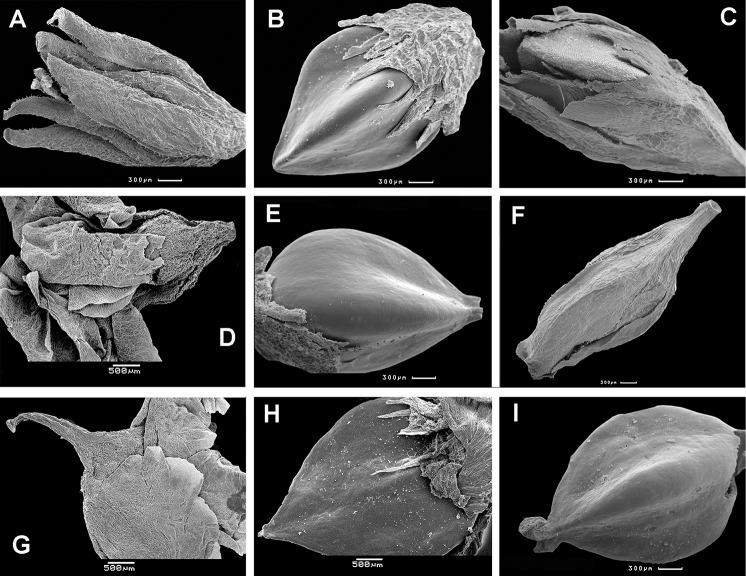
Flowers and achenes of *Atraphaxis* (SEM). (A) Perianth of *A. ovczinnikovii* (=*Bactria ovczinnikovii*) from Pamir. (B) Achene of *A. ovczinnikovii* (=*Bactria lazkovii*) from Tien Shan. (C, F) Achene and perianth of *A. atraphaxiformis.* (D, E) Perianth and achene of *A. ariana.* (G), Perianth of *A. badghysi* with subequal segments. (H) Dimeric achene **of** *A. badghysi.* (I) Achene of *A. avenia.* Scale bar for (A–C, E–F, I) = 300 μm, for (D, G–H) = 500 μm. Images: O. Yurtseva.

*Atraphaxis ovczinnikovii* from Tien Shan has the achenes ca. 2.5–3.0 × 1.8–2.0 mm, broadly ovoid, trigonous, shortly acuminate, with three free styles, obtuse ribs and flat or slightly concave equal faces, black, smooth and shiny, slightly excerted from the perianth (Figs. 6A and 16B).

*Polygonum salicornioides* (*P.* sect. *Spinescentia*) has rather large (3.5–4.0 × 2.8–3.4 mm), broadly ovoid trigonous achenes with three linear styles connate at the base into a stub and slightly inflated to the top, distinct narrow ribs, slightly concave faces, dark-brown, minutely tuberculate surface, and are enclosed by the perianth (Fig. 7B).

Within *Atraphaxis* s.str., the species with the campanulate perianth (*A. atraphaxiformis, A. toktogulica, A. tortuosa,* and *A. ariana*) have ovoid or pyriform trigonous achenes, ca. 2.5–4.5 × 1.5–3.0 mm, with obtuse or distinct ribs and equal, slightly concave faces (Figs. 9A, 9C, 11D, 11E, 16C and 16E). Similarly to *A. ovczinnikovii* from Pamir and *P. salicornioides*, they have three styles connate at base, which form a triquetrous stub at the top of the achene, and small capitate stigmae.

**Figure 17:**
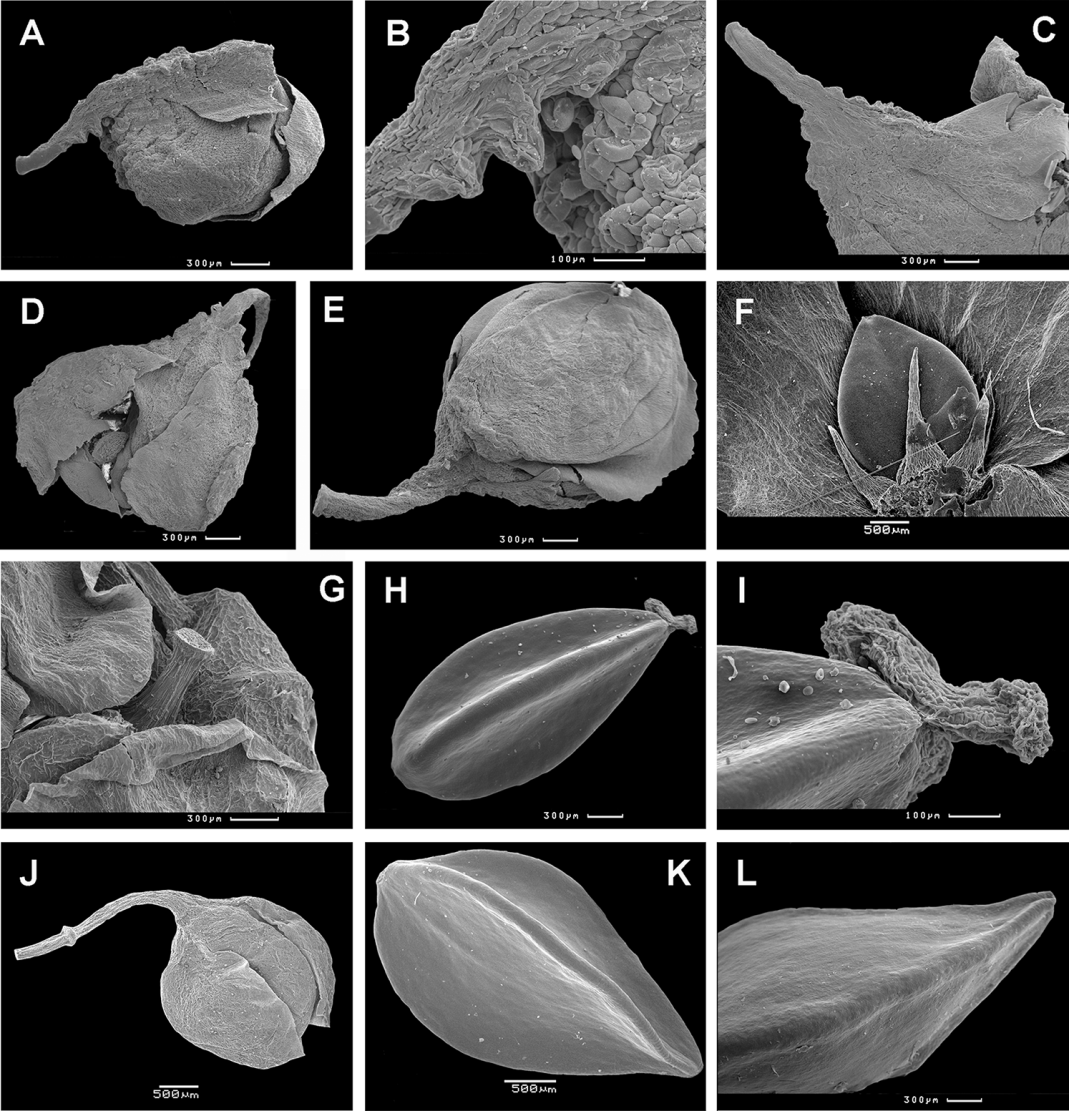
Flowers and achenes of *Atraphaxis* (SEM). (A–C) Flower bud and perianth of young flower of *A. avenia.* (D) Perianth of young flower of *A. virgata.* (E) Flower bud of *A. tournefortii*. (F) Achene of *A. laetevirens* in the perianth. (G) Perianth of *A. teretifolia* with a short filiform tube. (H and I) Achene of *A. teretifolia* with two free styles. (J) Flower bud of *A. pungens* with a long tube. (K) Achene of *A. pungens* with distinct ribs. (L) Achene top of *A. manshurica* with narrow ribs. Scale bar for (A, C–E, G and H, O) = 300 μm, for (B, I) = 100 μm, for (F, J, K) = 500 μm. Images: O. Yurtseva.

The members of *A.* sect. *Tragopyrum* have trigonous ovoid achenes 3.0–5.0 × 1.5–2.5 mm with slightly concave faces, obtuse or, rarely, distinct sharp ribs (*Atraphaxis badghysi, A. frutescens, A. manshurica, A. pungens*—Figs. 13C, 16H, 17K and 17L), and three free styles with large stigmata. *Atraphaxis avenia* (Fig. 16I) and *A. seravschanica* from the section *Tragopyrum* have the achenes with obtuse ribs, similar to those of *A. ariana* (Figs. 11D, 11E and 16E).

The members of *A.* sect. *Atraphaxis* (*A. spinosa, A. replicata, A. karataviensis, A. compacta, A. canescens*) differ by lenticular (rarely trigonous) achenes 3.0–3.5 × 2.0–3.5 mm (Fig. 14E) with two short free styles and large flattened stigmata. Lenticular achenes are also present in some species of the section *Tragopyrum* (*A. badghysi, A. laetevirens, A. billardierei*) along with trimeric ones (Figs. 16H and 17F).

*Atraphaxis teretifolia* from *A.* sect. *Physopyrum* has lanceolate trigonous achenes 2.5 × 1.5 mm, black and glossy, with concave faces, the ribs distinct near the top and obtuse below, and three short free styles with small capitate stigmae (Figs. 15D, 17H and 17I).

### Exocarp sculpture

**Figure 18:**
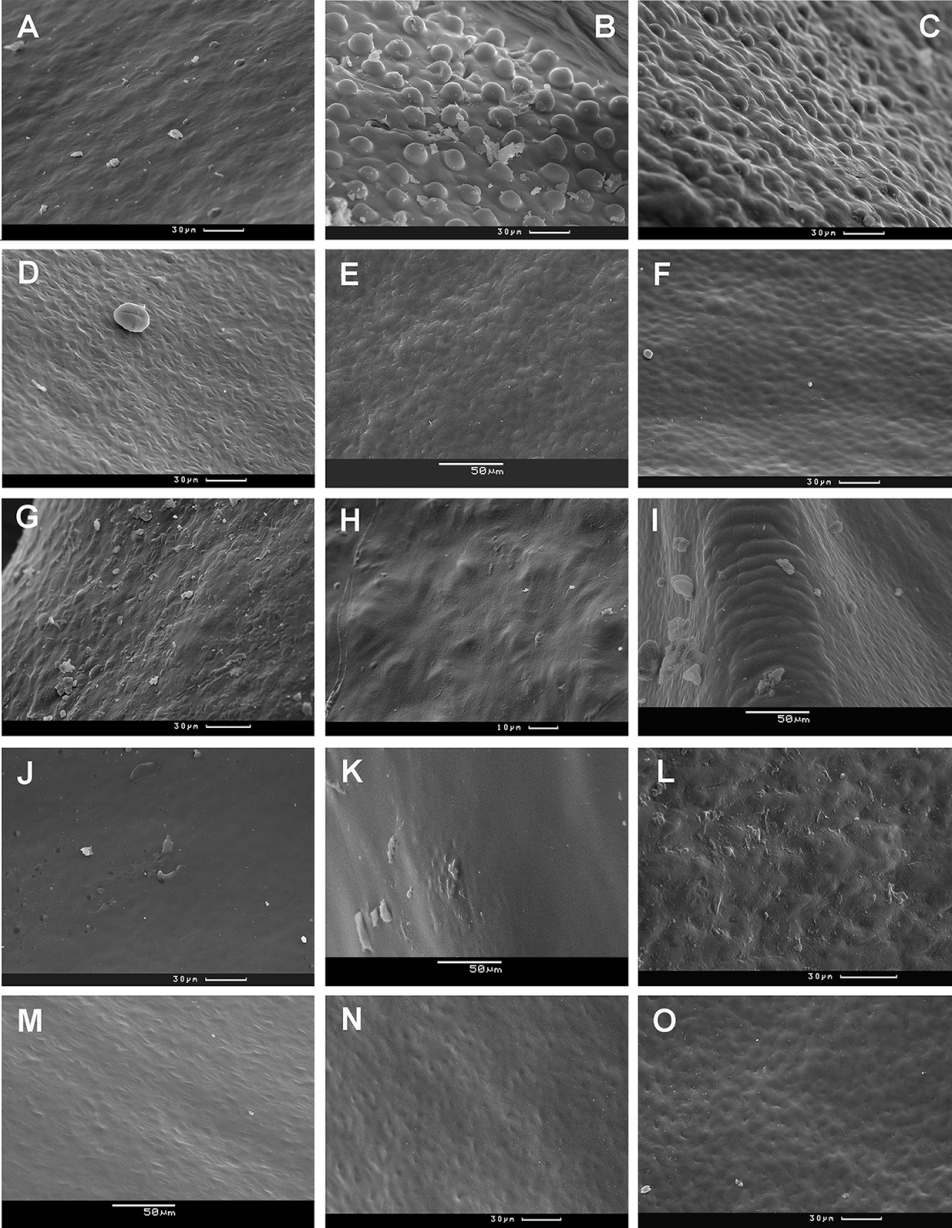
Achene surface of *Atraphaxis* (SEM). (A) *A. ovczinnikovii* (=*Bactria lazkovii*) from Tien Shan. (B–C) *A. atraphaxiformis.* (D) *A. ariana*. (E) *A. badghysi.* (F) *A. teretifolia.* (G) *A. avenia.* (H) *A. seravschanica.* (I) *A. caucasica*, view at the rib. (J) *A. laetevirens*. (K) *A. muschketowi.* (L) *A. manshurica*. (M) *A. pungens.* (N) *A. frutescens.* (O) *A. replicata.*. Smooth pitted (A, D–I, L–O), smooth (J, K), and tuberculate (B and C) achene surface. Scale bar for (A–D, F, G, J, L, N, O) = 30 μm, for (E, I, K, M) = 50 μm, and for (H) = 10 μm. Images: O. Yurtseva.

Achene surface is smooth or smooth-pitted in *A. ovczinnikovii* from Pamir (Fig. 5C) and Tien Shan (Figs. 6A and 18A).

The majority members of *Atraphaxis* have smooth, smooth-pitted, or minutely rugulate achene surface (Figs. 18D–18O). The premature achenes have minutely-rugulate surface (Figs. 18E–18H, 18I and 18L), which often becomes smooth-pitted or smooth in the mature achenes due to the development of cutin and wax covering (Figs. 18J and 18K). A smooth-pitted surface is formed by sunked loops of tessellate exocarp cells and raised periclinal walls (Figs. 18A, 18D, 18F, 18I and 18M–18O). Later, the pits are masked by a thick covering of cutin and wax, making the surface smoother. The surface is smooth or smooth-pitted, glossy in *A. ariana* (Figs. 11D, 11E, 16E and 18D). Additional ornaments in form of small protuberances or semirspheroid tubercules 15–20 μm in diameter are randomly scattered at the achene surface of *A. atraphaxiformis, A. toktogulica,* and some accessions *A. tortuosa* (Figs. 9A, 9C, 16C, 18B and 18C).

This minutely tuberculate surface is peculiar to the *Polygonum* sect. *Spinescentia* (Boissier, 1846; Boissier, 1879; Gross, 1913; Mozaffarian, 2012; Khosravi & Poormahdi, 2008; Tavakkoli et al., 2015). The exception is *Polygonum botuliforme* with smooth shiny achenes (Mozaffarian, 1988). However, this taxon, nested in the clade *Atraphaxis* in plastid phylogeny, seems to be a hybrid of *Atraphaxis*. Similarly to some *Polygonum* species (Yurtseva, 2001), the tubercles at the achene surface are thin-walled invaginations of the outer periclinal walls of exocarp cells, that possibly ease absorption of water.

### Pollen morphology

The majority of taxa in the tribe Polygoneae have prolate to subprolate, or spheroidal pollen grains, ellipsoidal in equatorial view, trilobed-circular in polar view, tri-colporate (rarely loxocolporate or syncolporate); the colpi are long, narrow, sometimes anastomosing at the poles, with well-developed elliptical ora (Table S6). The taxa of the tribe Polygoneae differ mainly in sporoderm ornamentation.

**Figure 19:**
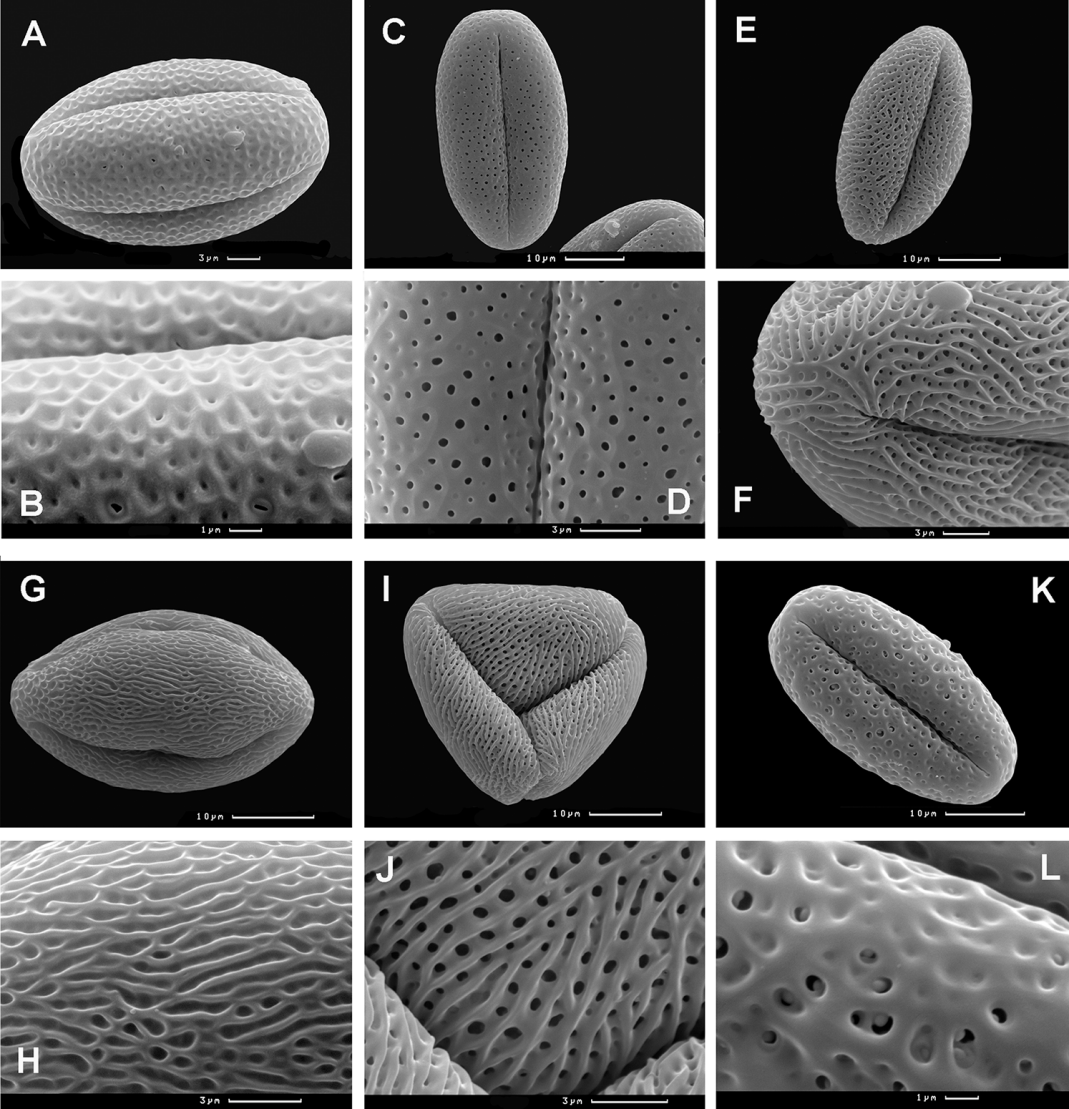
Tricolporate (A–H, K and L) and loxocolporate (I and J) pollen grains of *Atraphaxis,* equatorial view (SEM). (A and B) *A. ovczinnikovii* (=*Bactria ovczinnikovii*) from Pamir, microreticulato-foveolate sporoderm ornamentation with 4–6-angular pits and small perforations. (C and D) *A. toktogulica*, reticulato-perforate ornamentation (modified from Yurtseva, Severova & Bovina (2014)). (E and F) *A. kopetdaghensis*, striato-perforate ornamentation. (G and H) *A. ariana*, striato-foveolate ornamentation. (I and J) *A. virgata*, striato-perforate ornamentation. (K and L) *A. ovczinnikovii* (=*Bactria lazkovii*) from Tien Shan, foveolate-perforate ornamentation with rounded pits, some perforated. Scale bar for (A, D, F, H, J) = 3 μm, for (C, E, G, I, K) = 10 μm, for (B, L) = 1 μm. Images: O. Yurtseva & E. Severova.

Both accessions *Atraphaxis ovczinnikovii* and *Atraphaxis* s.str. have spheroidal to oblong-spheroidal pollen grains, tricolporate (rarely loxocolporate in *Atraphaxis* s.str.), elliptical or almost circular in equatorial view, rounded-trilobed in polar view; colpi distinct, long and deep; ora distinct, lalongate or circular (Fig. 19, see more in Yurtseva, Severova & Bovina, 2014).

*Atraphaxis ovczinnikovii* from Pamir differs by microreticulate-foveolate sporoderm surface (Figs. 19A and 19B): lumina with 4–6-angular pits are sharply defined at the edges; pits are rarely perforated at the bottom; perforations are few and small (0.1–0.2 μm in diameter), singular at the lumina (see also Yurtseva, Severova & Bovina, 2014). The plants from Tien Shan have sporoderm ornamentation that varies from microreticulate-foveolate, peculiar to the plants from Pamir, to foveolate-perforate (Figs. 19K and 19L), with rounded pits smoothened at the edges and rare perforations 0.5–1.5 μm in diameter, single at the lumina, or two, divided by thin bridges.

*Polygonum salicornioides* from *Polygonum* section *Spinescentia* has tectate-perforate sporoderm surface similar to that of *A. ovczinnikovii* from Pamir (unpublished), although it was described for *P. salicornioides* and *P. aridum* as reticulate-perforate (Tavakkoli et al., 2015). *Polygonum botuliforme* has striate-perforate sporoderm surface, fairly peculiar to *Atraphaxis* (Tavakkoli et al., 2015).

**Figure 20:**
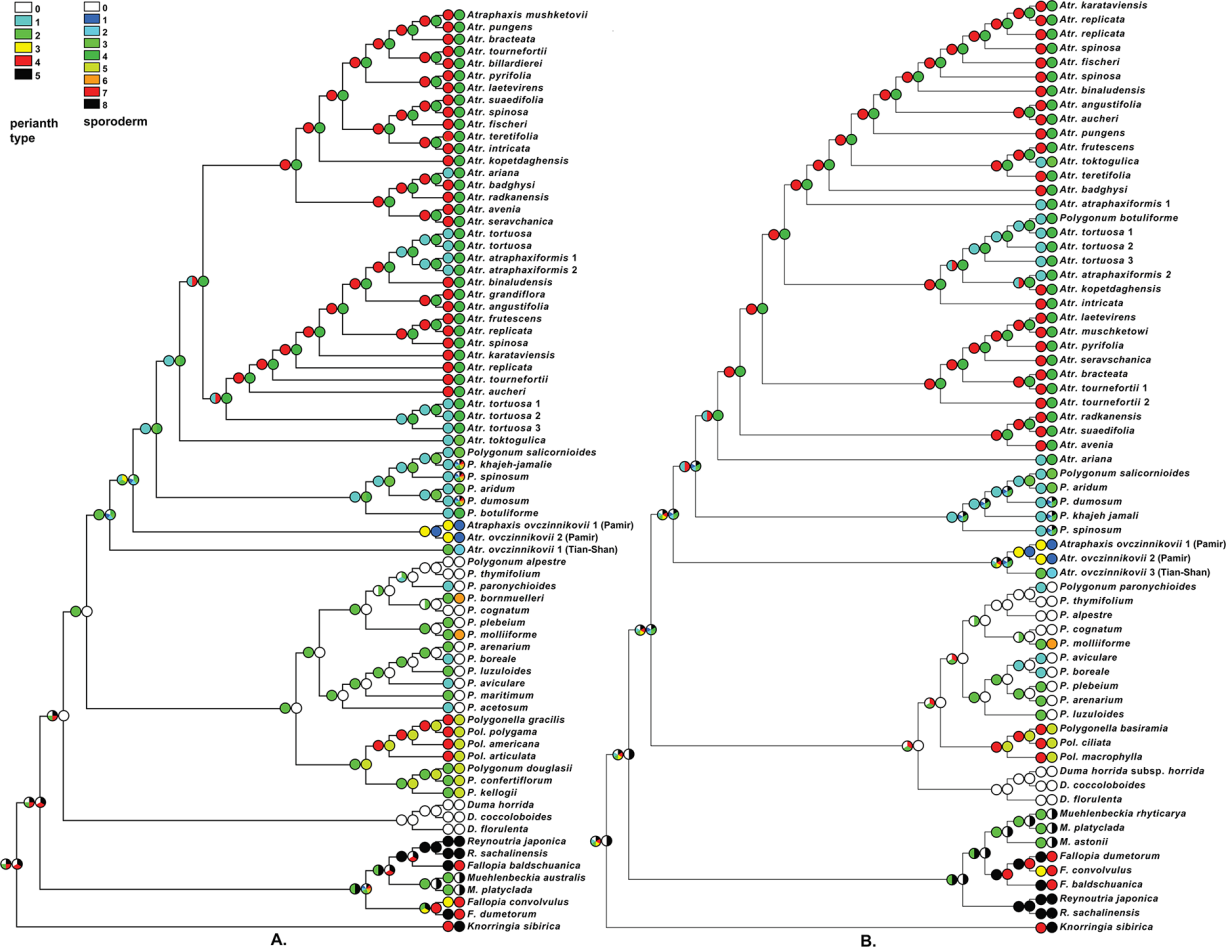
Most Parsimonious optimization (Maddison & Maddison, 2011) of the perianth morphology (A) and the ornamentation of sporoderm (B) using topology resulted the ML analysis of the ITS data set (A) and plastid data set (B). The description of the character states is given in “Materials and Methods” (see above). All character states were treated as “unordered.” Images: E. Mavrodiev.

The clade *Atraphaxis* s.str. includes the taxa with striate-perforate sporoderm ornamentation (Figs. 19E–19J): smoothened or distinct striae are divided by deep or shallow grooves with rows of perforations in the bottom (more in Hong, 1995; Yurtseva, Severova & Bovina, 2014). That type of sporoderm ornamentation is obviously a synapomorphy (Figs. 20A and 20B). However, *Atraphaxis toktogulica*, the most basal in the ITS topology (Figs. 1 and 20A), has the pollen surface with a transitional type of sporoderm ornamentation from reticulate-perforate to striate-perforate one: polygonal shallow perforated lumina are arranged in rows of 2–3 (4), which are oriented meridionally and divided laterally by hardly visible smoothened striae (Figs. 19C and 19D).

Therefore, the accessions *A. ovczinnikovii* from Pamir and Tien Shan share palynotype with microreticulate-foveolate sporoderm ornamentation. It is noteworthy, that *A. ovczinnikovii* from Tien Shan demonstrates variability of the pollen surface, which in some pollen grains is foveolate-perforate with large single or double perforations. Both variants are fairly different from that of *Polygonum* sect. *Spinescentia,* which is reticulate-perforate, or tectate-perforate, and in all the taxa distinct from striato-perforate sporoderm surface of *Atraphaxis* s.str. (Figs. 20A and 20B).

## Discussion

The results of ML and Bayesian analyses based on nrDNA ITS1&2 sequences and combined cpDNA *trn*L intron^(UAA)^ + *trn*L–F IGS and *rpl*32–*trn*L^(UAG)^ IGS sequences (Figs. 1 and 2) confirmed the division of the tribe Polygoneae in two major sister clades (RFM and ADP) previously recognized by Schuster, Reveal & Kron (2011b).

Compared to the results of Schuster, Reveal & Kron (2011b), the genus *Fallopia* (as sampled) appeared as polyphyletic (ITS topology, Fig. 1): the accession *Fallopia baldshuanica* is grouped with *Reynoutria*, and *F. convolvulus* and *F. dumetorum* form a separate clade. In plastid topology, *Fallopia* still remains monophyletic (Fig. 2). Contrary to the results of Schuster, Reveal & Kron (2011b), the genus *Duma* appeared to be the next clade of *Polygoneae* after the RFM clade in the ITS topology (Fig. 1), but is grouped with *Polygonum* s.l. in plastid topology (Fig. 2), which agrees with the findings of Schuster, Reveal & Kron (2011b).

### 

#### *Polygonum* s.l.

Genus *Polygonum* appears as highly polyphyletic (Figs. 1 and 2). *Polygonella* and the majority of current sections of the genus *Polygonum* (*Polygonum, Pseudomollia, Duravia*), excepting *P.* sect. *Spinescentia*, form a moderately supported *Polygonum* s.l. clade in both chloroplast and nuclear trees. Within *Polygonum* s.l. clade, in ITS topology (Fig. 1), *Polygonella* and *P.* sect. *Duravia* Watson (1873) from North America are monophyletic and form a clade sister to the rest *Polygonum* s.str. In the combined plastid topology (Fig. 2), in the absence of *P.* sect. *Duravia*, *Polygonella* falls sister to *Polygonum* s.str. That partly agrees with the results of Schuster, Reveal & Kron (2011b), and better agrees with the results of Schuster et al. (2015).

Palynological, anatomical, and morphological evidences show that *Polygonella* is closely related to *P.* sect. *Duravia* (Haraldson, 1978; Hong, Oh & Ronse De Craene, 2005; Hong, Ronse De Craene & Smets, 1998; Ronse Decraene, Hong & Smets, 2004). *Polygonella* and *P.* section *Duravia* share dimorphic sporoderm ornamentation defined by Hedberg (1946) as palynotype Duravia (see also Hong, Oh & Ronse De Craene, 2005). Both taxa are treated as subsections of *Polygonum* sect. *Duravia* (Ronse Decraene, Hong & Smets, 2004), and the members of *Polygonum* (Schuster, Reveal & Kron, 2011b; Schuster et al., 2015). Better sampling and more investigation is necessary, however present results show that the genus *Polygonella* (Michaux, 1803) and *Polygonum* section *Duravia* can be excluded from the widely circumscribed *Polygonum* (Schuster, Reveal & Kron, 2011b) treated separately. Greene (1903–1906) considered generic rank of *Duravia* Greene based on single flowers in cymes, persistent styles and the absence of articulation at the base of leaf blade. *Polygonella* is distinct from the other genera of Polygonaceae in having branches adnate to the stem and thus appearing to arise internodally (Horton, 1963). Perianth morphology also differentiates it from *P.* sect. *Duravia* and the rest of *Polygonum* s.str. (Fig. 20).

The division of the *Polygonum* s.str. in two subclades (part of *P.* sect. *Polygonum* from Eurasia and North America, and part of *P*. sect. *Polygonum* from SW and Central Asia) confirmes the ITS-based topologies obtained previously (Yurtseva et al., 2010; Schuster, Reveal & Kron, 2011b). The members of both subclades share palynotype Avicularia (Hedberg, 1946; Hong, Oh & Ronse De Craene, 2005). The latter of two subclades, however, includes *P. molliiforme* and *P. bornmuelleri* from *P.* section *Pseudomollia* (Boissier, 1879; Komarov, 1936), sharing palynotype Pseudomollia (defined by Hong, Oh & Ronse De Craene, 2005), therefore, the part of *Polygonum* sect. *Polygonum* from SW and Central Asia was recircumscribed by Schuster, Reveal & Kron (2011b) as *P.* sect. *Pseudomollia* Boissier (1879). Our analysis showed high variability in habit and perianth morphology in the members of both subclades of *Polygonum* s.str., but no special features were found to distinguish the members of two subclades (Yurtseva et al., 2010).

#### *Polygonum* sect. *Spinescentia and Atraphaxis*

*Polygonum* sect. *Spinescentia* is very distant from the rest of the *Polygonum*. The same-named clade appeared as the immediate sister of *Atraphaxis* s.str. clade (Figs. 1 and 2). This group resembles *Polygonum* in perianth shape and partition (Meisner, 1857; Boissier, 1879), but is fairly distinct from the other knotgrasses in habit, ochreae (Tavakkoli et al., 2015), and also possesses another type of sporoderm ornamentation (Fig. 20), which is never reticulate-perforate or tectate-perforate in *Polygonum* (see more in Table 1). The rank of *Polygonum* sect. *Spinescentia* clearly requires future clarification.

Indeed, the members of *Polygonum* section *Spinescentia* with the perianth divided to 1/2–3/4 in five equal-sized segments (Boissier, 1846; Boissier, 1879; Gross, 1913; Khosravi & Poormahdi, 2008; Mozaffarian, 1988; Mozaffarian, 2012) formally resemble some *Atraphaxis* species that were originally described in the genus *Polygonum* and later circumscribed with *Atraphaxis* as members of *Atraphaxis* s.str. clade (Yurtseva et al., 2010; Schuster, Reveal & Kron, 2011b). However, in contrast to urceolate perianth and leathery, rigid, oblong-lanceolate segments of *Polygonum* sect. *Spinescentia*, these members of *Atraphaxis* (*A. toktogulica, A. ariana, A. atraphaxiformis*, and *A. tortuosa*) have broadly-ovate or oblong-ovate, thin, petalloid segments, which are either white or brightly colored. They also resemble the rest of *Atraphaxis* in life history, habit, morphology of shoots and thyrses, leaf blades and ochreae. Unlike *Polygonum* sect. *Spinescentia*, they have tubulate ochreae 7–10 mm long, lacerate in two lateral aristate lanceolate lacinulae and inciso-dentate, short middle part at the side opposite to leaf blade.

Unlike *Polygonum* section *Spinescentia,* with tectate-perforate (own observations) or reticulato-perforate (Tavakkoli et al., 2015) sporoderm ornamentation, *A. toktogulica, A. ariana, A. atraphaxiformis*, and *A. tortuosa* share striato-perforate sporoderm ornamentation with the rest members of the clade *Atraphaxis* s.str. (Yurtseva, Severova & Bovina, 2014). The recently proposed inclusion of *Polygonum* sect. *Spinescentia* in the genus *Atraphaxis* (Tavakkoli et al., 2015) is fairly questionable from a morphological standpoint. This taxonomical decision simply resulted in the loss of the morphological identity of the genus *Atraphaxis* sensu Linnaeus (1753), Marschall Bieberstein (1819) and Jaubert & Spach (1844–1846).

#### *Atraphaxis* s.str.

Despite variability of perianth morphology within the clade *Atraphaxis* (see **Results**) deep similarity in life history, habit, inflorescence structure, pollen shape and size, and striato-perforate sporoderm ornamentation suggests a narrow delimitation of *Atraphaxis* corresponding to the clade *Atraphaxis* s.str. in our own plastid and ITS-based reconstructions. Petalloid segments and striato-perforate sporoderm ornamentation of pollen are unique morphological synapomorphies appeared in the genus *Atraphaxis*.

The campanulate perianth with equal-sized segments present in some *Atraphaxis* species looks as ancestral state for the more advanced perianth with accrescent inner segments and a filiform tube as long as a pedicel. The latter perianth type appears and predominates only in the clade *Atraphaxis* s.str., being correlated with compact abracteose or bracteose thyrses of congested cymes of flowers, making the floral units compact and showy.

Due to the flower buds and premature flowers of all *Atraphaxis* species have the campanulte perianth with equal-sized segments and rather short filiform base of tube, the perianth with enlarged inner segments and long filiform tube seems to have appeared through ontogenetic transformation of the perianth by the elongation of the basal part of the tube and by the accrescence of the inner segments. Within the clade *Atraphaxis* s.str., mainly basal positions of the species with presumably ancestral character state (*A. toktogulica, A. ariana, A. tortuosa*) and distal positions of the species with transitional perianth type (*A. anfustifolia, A. grandiflora, A. bracteata, A. badghysi*), or advanced type allow to suggest homoplastic origin of the specialized perianth widespread in *Atraphaxis* s.str.

The advanced perianth type provides more effective pollination in compact thyrses with congested cymes of flowers and better dispersal of the achenes. Long filiform basal part of a tube joined to an equally long pedicel puts out a flower from the thyrse and rises up the swing amplitude of the fruit, increasing the chance of far-distant dispersal. Accrescent inner segments serve for better protection of the ovary and facilitate dispersal of fruits by wind in open communities. *Atraphaxis teretifolia* has the shortest filiform tube (Fig. 18; Table S4), but the longest pedicel (6–7 mm). This species from sandy deserts of Kazakhstan realizes another way of dispersal of the achenes hidden in a spherical perianth, by rolling on the surface, while the shortest tube does not prevent slipping along sandy surface.

Papillae found at the perianth tube of *A. toktogulica, A. atraphaxiformis,* and *A. ariana* are present as well at the perianth of some *Polygonum* s.str., *P.* sect. *Spinescentia, Oxygonum,* and *Fagopyrum* (Hong, Ronse De Craene & Smets, 1998), far distant from *Atraphaxis* and *Polygonum* in phylogenetic reconstructions (Sanchez et al., 2011; Schuster, Reveal & Kron, 2011b). Along with protective functions, papillae at the perianth surface are considered as an adaptation to insect-pollination mechanism (Hong, Ronse De Craene & Smets, 1998), playing a tactil role for recognition by pollinators (Whitney et al., 2009; Ojeda, Francisco-Ortega & Cronk, 2009). Being present at the perianths of *Polygonum* sect. *Spinescentia, A. toktogulica, A. atraphaxiformis,* and *A. ariana*, the papillae are absend from more specialized perianths of the majority of *Atraphaxis* species characterized by accrescent inner segments and filiform basal part of tube, compact thyrses of congested cymes of flowers, and striato-perforate sporoderm ornamentation, a morphological complex, that was possibly caused by another pollination mode predominating in *Atraphaxis.*

The subclades recovered in the clade *Atraphaxis* s.str. in the ITS-based phylogeny do not correspond to the sections *Atraphaxis, Tragopyrum*, and *Physopyrum,* which previously were described in the genus *Atraphaxis* (Table S2). However, in the plastid phylogeny, the members of the section *Atraphaxis* with tetrameric perianth and dimeric gynoecium form a separate subclade.

Flower merosity was traditionally the most important characteristic discriminating the section *Atraphaxis* (*A. spinosa, A. fischeri, A. canescens, A. replicata, A. karataviensis, A. compacta*) from the section *Tragopyrum*. According to Krasnov (1888), *A. muschketowi, A. laetevirens,* and *A. variabilis* (=*A. billardierei*) from the section *Tragopyrum* with a pentamerous perianth, in arid conditions have smaller flowers with four segments and dimeric gynoecium. Variable flower merosity in some taxa might be a result of intrageneric hybridization. Finally, tetrameric perianth and dimeric gynoecium peculiar for the section *Atraphaxis* might be useful for the achene and fruit dispersal in open sandy deserts of Central Asia. The lenticular achenes hidden between two flat papery inner segments possibly have higher windage, than the fruits with three inner segments hiding triquetrous achenes. Sharp and flat ribs present at the lenticular achenes of the section *Atraphaxis* possibly facilitate dispersal of the achenes by wind in open steppe and semi-desert communities.

#### *Atraphaxis* sect. *Ovczinnikovia*

The major focus of our research is *Atraphaxis* sect. *Ovczinnikovia* O.V. Yurtseva ex S. Tavakkoli. We already demonstrated that the morphologically remarkable *Polygonum ovczinnikovii* from Pamir is distinct from the rest members of *Atraphaxis* in pollen morphology (Yurtseva et al., 2012a; Yurtseva, Severova & Bovina, 2014). The recent and well-supported phylogenetic placement of *Polygonum* section *Spinescentia* as a sister to *Atraphaxis* s.str. (Tavakkoli et al., 2015, see also Figs. 1 and 2) followed by the sistership of *A. ovczinnikovii* from Tien Shan to the clade (*Atraphaxis* s.str. + *Polygonum* sect. *Spinescentia*) (Figs. 1 and 2) makes the recent inclusion of *A. ovczinnikovii* from Tien Shan in *Atraphaxis* (Tavakkoli et al., 2015) very questionable.

Our analysis of combined plastid matrix showed, that Pamirian and Tian-Shanian accessions of *Atraphaxis ovczinnikovii* are not intermixed with each other, but appeared as well supported sister groups: one includes the Pamirian plants, with the Tian Shanian accession as an immediate sister (Fig. 2). Moreover, *Atraphaxis* sect. *Ovczinnikovia* was recognized as paraphyletic based on the results of the phylogenetic analysis of the ITS matrix, with Tien-Shanian accession fell as a sister to *Atraphaxis* s.l. clade (incl. *A.* sect. *Ovczinnikovia* and *Polygonum sect. Spinescentia)*, as recently circumscribed by Tavakkoli et al. (2015) (Fig. 1).

These results may be arguing for the hybrid origin of the Tien Shanian accession of *Atraphaxis ovczinnikovii*. However, much more investigation is necessary due to the well-known issues with ITS marker (summarized in Álvarez & Wendel (2003), Baldwin et al. (1995) and Poczai & Hyvönen (2010)). This may be the subject of future considerations, but here we are arguing for the recognition of the Pamirian and Tian-Shanian samples of *Atraphaxis ovczinnikovii* as a new genus that seems to be well distinguishable from both *Atraphaxis* s.str. and *Polygonum* sect. *Spinescentia* based on morphological or phylogenetic standpoints (Figs. 1, 2, 4–6, 16A, 16B, 19A, 19B, 19K, 19L and 20).

Hereinafter, we assign both accessions *A. ovczinnikovii* to the genus *Bactria* O.V. Yurtseva & E. Mavrodiev gen. nov., the collection from Pamir (Tajikistan) assigned as *Bactria ovczinnikovii* (Chukav.) O.V. Yurtseva & E.V. Mavrodiev comb. nov., and the collection by G. Lazkov from Tien Shan (Kyrgyzstan) assigned as *Bactria lazkovii* O.V. Yurtseva & E.V. Mavrodiev spec. nov. Both species are small shrubs with frondulose thyrses and campanulate perianth divided to 4/5 (*B. lazkovii*) and to 8/10 (*B. ovczinnikovii*) in 5(6) equal-sized petalloid segments with papillae at the segment edge.

*Bactria ovczinnikovii* has microreticulate-foveolate sporoderm ornamentation with 4–6 angular pits, sharply defined, some with small perforations 0.1–0.2 μm in diameter (Figs. 19K and 19L). Similar palynotype was described also in *Fagopyrum* (Van Leeuwen, Punt & Hoen, 1988) and *Parapteropyrum* (Hong, 1995) from the tribe Fagopyreae (Sanchez et al., 2011), as well as in *Pteropyrum* (Hong, 1995) and *Calligonum* (Bao & Li, 1993) from the tribe Calligoneae (Sanchez et al., 2011).

The pollen sample of *Bactria lazkovii* includes the pollen with the sporoderm ornamentation peculiar for *Bactria ovczinnikovii,* and the pollen with foveolato-perforate ornamentation of the sporoderm, with small rounded foveolae and rare single or double perforations 0.5–1.5 μm in diameter unique in Polygoneae (Figs. 19A and 19B), as well as some transitional types. This findings need additional study.

Therefore, the microreticulato-foveolate sporoderm ornamentation of *Bactria* is similar to the tectate-perforate ornamentation detected in *Polygonum salicornioides*, as well as to the reticulate-perforate ornamentation found in *Polygonum aridum* from *Polygonum* sect. *Spinescentia* (Tavakkoli et al., 2015). These pollen types are very distinct from the striato-perforate sporoderm ornamentation of narrowly defined *Atraphaxis* (Hong, 1995; Yurtseva, Severova & Bovina, 2014), which presumably appeared by way of arranging the polygonal lumina into rows, and rising the lateral edges of adjacent lumina, resulted in formation of the striae (see also Yurtseva, Severova & Bovina, 2014).

## Conclusions

1. *Atraphaxis ovczinnikovii* (*Atraphaxis* sect. *Ovczinnikovia*) belongs to the genus *Bactria* O.V. Yurtseva & E.V. Mavrodiev gen. nov.
2. The newly described genus is circumscribed with two morphologically and geographically distinct species: *Bactria ovczinnikovii* (Czukav.) O.V. Yurtseva & E.V. Mavrodiev comb. nov., and *B. lazkovii* O.V. Yurtseva & E.V. Mavrodiev spec. nov.
3. The recently proposed inclusion of *Polygonum* sect. *Spinescentia* Boissier (*A.* sect. *Polygonoides* S. Tavakkoli, Kaz. Osaloo & Mozaff.) in *Atraphaxis* is fairly questionable from a morphological standpoint and resulted in the loss of the morphological identity of *Atraphaxis* sensu Linnaeus (1753), Marschall Bieberstein (1819) and Jaubert & Spach (1844–1846). The rank of *Polygonum* sect. *Spinescentia* requires further clarification.
4. Our results are arguing for the narrow delimitation of *Atraphaxis* characterized by petalloid segments of perianth and striato-perforate sporoderm ornamentation of pollen as main morphological synapomorphies.
5. Due to the polyphyly of the genus *Polygonum* s.l., the generic composition of the tribe *Polygoneae* may also requires future reappraisals, but at the moment much more work is necessary to resolve the problem.

The key to the genera was constructed using own observations and reference data (Tables 1 and S4–S5).

## Key to the genera *Polygonum, Polygonella, Atraphaxis*, and *Bactria*

1. Perennial or annual herbs, or undershrubs with internodal branching, inflorescence bracteose, terminal with tightly congested cymes, perianth of five equal-sized or unequal segments, with long filiform basal part of tube. North America*Polygonella*
  —Shrubs, undershrubs or herbs with branches not adnate to stems, inflorescence frondose or bracteose, terminal or lateral, cymes congested or spaced, perianth of four-five segments, equal or inner ones greatly enlarged 2
2. Annual, rarely perennial herbs with campanulate or urceolate perianth divided to 1/2–5/6 in five equal-sized segments petalloid, green along midveins, with wide white or anthocyan-colored margin, flat, obtuse, or cucullate and keeled. Ochreae tubulate, with 5–11 veins, lacerate or fimbriate. Worldwide.*Polygonum sect. Polygonum* and *P*. sect. *Duravia*
  —Shrubs, dwarf shrubs, or undershrubs, perianth campanulate or urceolate, or with accrescent inner segments and long filiform tube. SW and Central Asia, Eurasia3
3. Perianth large (6.5–14.5 mm), mostly with four-five unequal segments (two-three inner segments accescent in fruits and much larger then two outer segments), and filiform tube 2.5–7.5 mm long; rarely perianth campanulate, divided to 1/2–3/4 in five equal or subequal segments, with tube 1–3 mm long, the filiform basal part of tube no more then 0.4–1.0 mm long, however the segments always are petalloid, thin, widen to ends, white or brightly colored, fully enclosing the achene. Stamens 6 to 8; styles 2 or 3. Shrubs or undershrubs with compact bracteose, or rarely frondulose terminal or lateral thyrses. Ochreae 3–10 mm, tubulate, with two veins, the upper part membranous, transparent, lacerate in two lateral aristate lacinulae and finely serrate-incised middle part. Ochreae in thyrses oblique-funnel form, membranous, transparent, with a small herbaceous outgrowth or a narrow keel. Mts of SW and Central Asia, steppes of Eurasia*Atraphaxis*
  —Perianth 2.0–6.5 mm, campanulate or urceolate, with 5(6) equal-sized segments, petalloid or leathery, tube widely funnel-form or cup-shaped, filiform basal part of tube does not exceed 0.3 mm. Stamens 8(9); styles 3. Ochreae lanceolate-tubulate, split in two acuminate lacinulae4
4. Dwarf shrubs with shoots never prickly, perianth divided almost to base (4/5–8/10) in 5(6) equal-sized segments petalloid, glabrous, with papillae only at segment edge. Pamir, Tien Shan*Bactria*
  —Dwarf shrubs or undershrubs, some with prickly shoots, with perianth divided to 1/2–3/4, fully puberulent or glabrous, at last case without papillae at segment edge5
5. Cushion-shaped caespitose undershrubs, dwarf shrubs, or perennials, some with prickly shoots. Perianth urceolate, 2–6 mm long, fully densely shortly puberulent, rarely glabrous (*P. botuliforme* from Iran), divided to 2/3–3/4 in segments oblong-elliptical or lanceolate, gradually narrowed to the top, leathery (coriaceous), rigid, green-purple, with a narrow (less then 0.2 mm) membranous margin. Ochreae tubulate, 3–7 mm long, membranous, truncate and shortly bidentate, later bilacerate, without veins, or with 2–6 veins. Endemics of Iran*Polygonum* sect. *Spinescentia*
  —Small shrubs or undershrubs, shoots not prickly. Perianth urceolate, 2–3 mm, glabrous or papillate only at the tube, divided to 1/2–3/4, segments thin, petalloid, green, with wide white or anthocyan-colored margin, flat, obtuse, or keeled and cucullate. Ochreae tubulate, 3–10 mm, membranous, with 5–11 veins, lacerate-fimbriate. Mts of SW and Central Asia*Polygonum*

## New names and combinations

***Bactria* O.V. Yurtseva & E.V. Mavrodiev,** genus novum.

**Type**: *Bactria ovczinnikovii* (Czukav.) O.V. Yurtseva & E.V. Mavrodiev.

= *Atraphaxis* section *Ovczinnikovia* O.V. Yurtseva ex S. Tavakolli (2015, e. publ. 2014), Plant Systematics and Evolution 301(4):1157–1170.

Type: *A. ovczinnikovii* (Czukav.) O.V. Yurtseva.

Small shrub 10–30 cm tall with divaricately-branched woody shoots covered with gray bark, fibrously desintegrated, annual shoots leafy, shortly puberulent. Leaf blades thick, coriaceous, broadly-ovate to oblong-elliptical, narrowed to a petiole 1–2 mm long, obtuse or shortly acuminate, revolute at margin, joined with articulation. Thyrses terminal, leafy, with 3–7 clusters of 1–2(3) flowers. Ochreae 2–4 mm, in thyrses ovate, cup-form inflated under the petiole, at vegetative shoots lanceolate-tubulate, at base herbaceous and shortly papillate, above membranous, transparent, later bidentate or bilacerate. Perianth campanulate, enclosing the achene or not, 2–4 mm long, (to 3–6 mm in fruiting), divided to 4/5–8/10 in 5(6) equal segments papillate at margin. Stamens 8(9). Pollen tricolporate, oblong-spheroidal to spheroidal (P/E = 1.1–1.4), elliptical in equatorial view, rounded-trilobed in polar view, sporoderm ornamentation microreticulate or foveolate-perforate. Styles 3, stigmata capitate. Achene 2.5–5.0 × 1.8–2.8 mm, ovoid, trigonous, glabrous, shiny.

**Distribution:** Pamir-Alay (South Tajikistan), Tien Shan (Kyrgyzstan).

**Name:** The genus is named for Bactria, an ancient historical region covering the territories of modern Tajikistan, Afghanistan, Pakistan to south of the Pamir Mountains, and the Amu-Darya.

**Affinity:** *Bactria* resembles *Atraphaxis* by shrubby habit, terminal thyrses, coriaceous broadly-ovate leaves, papillate covering of annual shoots and leaves, but differs by perianth morphology. It resembles *Polygonum* by campanulate perianth, but differs by partition into segments almost to base and papillae at segment edge. Pollen grains resemble pollen of *Atraphaxis* and *Polygonum* in shape and size, but differ by microreticulate or foveolate-perforate sporoderm ornamentation.

The genus includes two species.

## Key to the species of *Bactria*

1. Perianth divided almost to base (to 5/6–8/10) in 5(6) equal segments lanceolate, gradually acuminate, slightly keeled and cucullate, concave, leathery (coriaceous), purple-green with a narrow pinkish margin; the tube funnel-form. Dwarf shrub 20–30 cm tall. Leaf blades grayish-green, thick, broadly ovate, shortly acuminate or obtuse, with prominent midvein and laterals below. Stamens 8(9), styles 3, connate at base. Endemic of Pamir (Tajikistan)1. *B. ovczinnikovii*
  —Perianth divided to 4/5–5/6 in 5 equal broadly ovate or elliptical segments, obtuse, membranous, petalloid, with wide pink margin; tube cup-shaped or sacciform. Dwarf shrubs 10–15 cm tall. Leaf blades bright-green, coriaceous, oblong-elliptical or lanceolate, acuminate, with prominent midvein below, gradually narrowed to a pedicel. Stamens 8, styles 3, free. Endemic of Tien Shan (Kyrgyzstan)2. *B. lazkovii*

1. ***Bactria ovczinnikovii* (Czukav.) O.V. Yurtseva & E.V. Mavrodiev**, comb. nov. ≡ *Polygonum ovczinnikovii* Czukav. (1962) in Izv. Akad. Nauk Tadzhiksk. SSR, Otd. Biol. Nauk 2(9): 62; Chukav. (1968) in Fl. Tadzhiksk. SSR, 3: 250. ≡ *Atraphaxis ovczinnikovii* (Czukav.) O.V. Yurtseva (2014), Plant Systematics and Evolution 300(4): 763 (Figs. S2, 3B, 4, 5, 16A, 19A and 19B).
  **Holotype:** South Tajikistan, village Bag on the river Pyandzh, red and gray sandstones on the left bank of the Aarzy-Su river. 2 June 1960. *V. Botschantzev & T. Egorova, 814*. (LE!).
  **Distribution:** Endemic of Pamir-Alay (Tajikistan).
2. ***Bactria lazkovii* O.V. Yurtseva & E.V. Mavrodiev**, spec. nov.
  **Holotype:** Kyrgyzstan, Naryn reg., Dzhumgal d., Kavak-Too Ridge, 5 km N of village Sary-Bulun, at rocks. 7 July 2006. *G.A. Lazkov, 24*. (MW!; isotypes: B, LE) (Figs. S1, 3A, 6, 16B, 18A, 19K and 19L).

Dwarf shrub 10–20 cm tall, much branched with spread woody shoots departed under 45°, covered with gray, longitudinally exfoliating bark. Annual shoots 3–5 cm, leafy, finely ribbed, foxy-brown, densely puberulent, some terminated by thyrses of 4–5 clusters of 1(2) flowers. Ochreae up to 2–4 mm, lanceolate-tubulate, herbaceous, brownish and short-puberulent at base, membranous and transparent above, pubescent along the keel and split in two lanceolate lacinulae, each with single reddish vein. In thyrses the ochreae are 4–5 mm, cup-shape inflated, at base greenish-brown and densely puberulent, above membranous and transparent, bidentate or bilacerate. Leaves alternate, bright green, coriaceous, 7–10 × 2.5–3 mm, oblong-elliptical, apex obtuse or mucronate, base cuneate, gradually narrowed into a petiole 1 mm long, joined with articulation, margin revolute, adaxially glabrous, abaxially with conspicuous reddish midvein dilatated to the base and shortly puberulent along with a petiole.

Flowers one or two per a cyme, with very short pedicells 0.3–0.5 mm, hidden in the ochreae, easy to fall down.

Perianth 2.5–3.0 mm, campanulate, divided to 4/5–5/6 in five equal segments, broadly-elliptical or broadly-ovate, green-brownish along the keel, with wide pinkish margin papillate at the edge. Segments oblong-elliptical or ovate, 2.0–2.5 × 1.8–2.0 mm, the outer segments slightly keeled, cucullate, inner segments flat, obtuse. Tube cup-shaped or sacciform.

Stamens 8; filaments lanceolate, inner ones twice as wide as the outer ones, expanded to base. Anthers orbiculate, pink. Ovary ovoid, styles 3, free from base; stigmata capitate.

Achenes 2.5–3.0 × 1.8–2.0 mm, ovoid, trigonous, with flat faces and obtuse ribs, shiny, black, surface minutely foveolate or smooth.

**Phenology:** fl. June–July, fr. July–August.

**Distribution and habitat:** endemic of Central Tien Shan (Kyrgyzstan), at rooks.

**Etymology:** The species is named for G.A. Lazkov, the collector of the type specimen.

**Affinity:** The species is similar to *Bactria ovczinnikovii* in habit, terminal frondulose thyrses, papillate covering of annual shoots, ochreae, leaves, but differs in foxy-brown slightly ribbed annual shoots, oblong-ovate leaf blades puberulent only at midvein abaxially, obtuse petalloid segments, cup-shaped or sacciform base of tube, flat achene sides and free styles not connate at base.

## Supplemental Information

10.7717/peerj.1977/supp-1 Supplemental Information 1 Taxonomic history of position of the genus Atraphaxis L. in Polygonoideae (Polygoneceae).

10.7717/peerj.1977/supp-2 Supplemental Information 2 Classification history of the genus Atraphaxis L.

10.7717/peerj.1977/supp-3 Supplemental Information 3 Taxa, voucher information, current and GenBank accession numbers used in the study.

10.7717/peerj.1977/supp-4 Supplemental Information 4 Characteristics of the perianth and the achene in *Bactria*, *Polygonum* sect. *Spinescentia*, and *Atraphaxis* s.str.

10.7717/peerj.1977/supp-5 Supplemental Information 5 Some perianth characteristics of the members of the tribe Polygoneae.

10.7717/peerj.1977/supp-6 Supplemental Information 6 Main pollen characteristics in the tribe Polygoneae.

10.7717/peerj.1977/supp-7 Supplemental Information 7 Origin of the material used for the SEM micrographs.

10.7717/peerj.1977/supp-8 Supplemental Information 8 Origin of the material used for LM micrographs.

10.7717/peerj.1977/supp-9 Supplemental Information 9 Atraphaxis ovczinnikovii from Tien Shan (= Bactria lazkovii O.V. Yurtseva & E.V. Mavrodiev sp. nov.). Kyrgyzstan, Naryn reg., Dzhumgal d., Kavak-Too Ridge, 5 km N of village Sary-Bulun, at rocks. 07.07.2006. n 24, G.A. Lazkov (MW, Holotypus). A dwarf shrub with divaricately branched shoots and frondulose terminal thyrses. Images: O. Yurtseva.

10.7717/peerj.1977/supp-10 Supplemental Information 10 Atraphaxis ovczinnikovii (= Bactria ovczinnikovii (Czuk.) O.V. Yurtseva et E.A. Mavrodiev; Polygonum ovczinnikovii Chukav). Tadjikistan, Khablon reg. Shuroabad d. the right bank of the river Piandzh, Bag × Bakhorak. h = 110 m a.s.l. 2.06.1960. No. 1624. C. Yu. A dwarf shrub with divaricately branched shoots and frondulose terminal thyrses. Images: O. Yurtseva.

10.7717/peerj.1977/supp-11 Supplemental Information 11 Polygonum salicornioides Jaub. et Spach (= Pol. oligophyllum Boiss.), Persia australis, In alpe Kuh-Delu. 10 June 1842. No. 468. Th. Kotschy. (LE, Isotypus). A dwarf undershrub with branched annual shoots and axillary cymes of flowers in axils of deciduous leaves. Images: O. Yurtseva.

10.7717/peerj.1977/supp-12 Supplemental Information 12 Atraphaxis ariana Grigorj. Turkmenistan, environs of V. Kushka. In cliff Shur-Sufi. 5.05.1928. K.P. Volkov. (LE, Typus). An undershrub or shrub with buried in sand manyheaded caudex and numerousl elongated generative shoots terminated by bracteose thyrses, or racemes of thyrses. Images: O. Yurtseva.

10.7717/peerj.1977/supp-13 Supplemental Information 13 Atraphaxis toktogulica (Lazkov). T.M. Schust. & Reveal. Kyrgyzstan, Toktogul d., Karajigach, left board of say Tor-Kolot. 5.07.1973. s.n. Ajdarova et al. (LE, Holotypus). An undershrub or shrub with manyheaded caudex and numerous elongated generative shoots terminated by bracteose thyrses or racemes of thyrses. Images: O. Yurtseva.

10.7717/peerj.1977/supp-14 Supplemental Information 14 Atraphaxis atraphaxiformis (Botsch.) T.M. Schust. & Reveal. [Kyrgyzstan], Alay Ridge, the interstream of the Isphara and the Sokh. Mts Baytok. At limestone rocks.15.07.1962. n.93. V.P. Botschantzev (LE, Typus). A shrub with elongated intensively branched shoots terminated by bracteose thyrses. Images: O. Yurtseva.

10.7717/peerj.1977/supp-15 Supplemental Information 15 Atraphaxis tortuosa Lozinsk. [Mongolia occidentalis]: the Urot terra, Muni-Ula, (the north bend of the river Huang-He) ½.05.1872. Przhewalsky (LE, Isotypus). (A) A shrub with elongated intensively branched shoots, terminated by bracteose thyrses (above). (B) A fragment of bracteose thyrse with axillary cymes of flowers and the achene (below). Images: O. Yurtseva.

10.7717/peerj.1977/supp-16 Supplemental Information 16 Atraphaxis avenia Botsch. Kyrgyzstan, Alay Ridge, the Gulcha basin, the river Irgailysu above Sufi-Kurgan. 16.07.1987. No.407. M.G. Pimenov, E.V. Klujkov (MW). A shrub with elongatated generative shoots terminated by bracteose thyrses. Images: O. Yurtseva.

10.7717/peerj.1977/supp-17 Supplemental Information 17 Atraphaxis virgata (Regel) Krasn. Kazakhstan, South Kazakhstan reg., Tyulkubass d., the riverhead of Mashat, “Mashat,” the break stone slopes nearby the river. 29.09.2012. V.A. Sagalaev (MHA). A shrub with elongated generative annual shoots each terminated by a raceme of bracteose thyrses, the central shoot with two paracladia. Images: O. Yurtseva.

10.7717/peerj.1977/supp-18 Supplemental Information 18 Atraphaxis laetevrens (Ledeb.) Jaub. & Spach. Kazakhstan, Dzhungar Alatau, nothers spurs, Mts. Kaykan to the NE of Glinovka. At stony slopes. 18.06.1959. n.s. V.P. Goloskokov (MW). A shrub with lateral abracteose thyrses. Images: O. Yurtseva.

10.7717/peerj.1977/supp-19 Supplemental Information 19 Atraphaxis pyrifolia Bunge. Tajikistan, Badakhshan, Shugnan d., the left board of the Gunt river, Vozh × Shtamm. 02.08.2011. No. 26. E.V. Klujkov, E.A. Zakharova, U.A. Ukrainskaja (MW). A shrub with elongated prickly shoots and second-order branchlets, and congested thyrses lateral at the second-year branchlets. Images: O. Yurtseva.

10.7717/peerj.1977/supp-20 Supplemental Information 20 Atraphaxis spinosa L. Armenia, Ararat reg., environs of the Mt. Goravan, the refuge «Goravan Sands», sandy slopes.13.08.2012. D. Lyskov (MW). A shrub with prickly elongated shoots and branchlets. Images: O. Yurtseva.

10.7717/peerj.1977/supp-21 Supplemental Information 21 Atraphaxis spinosa L., Afghanistan, Prov. Samangan, N-Hänge des Koh-i-Chungar, 12 km SW Rabatak, 1,550 m, Lößboden; 68/21–36/05. 7.6.1978. n. 31519. Leg. et det. D. Podlech. (LE). A shrub with elongated annual shoots and axillary short thyrses. Images: O. Yurtseva.

## Additional Information and Declarations

### DNA Deposition

The following information was supplied regarding the deposition of DNA sequences: GenBank accession numbers are provided in Table S3.

### Data Deposition

The following information was supplied regarding data availability: The type specimen of *Bactria lazkovii* O.V. Yurtseva & E.V. Mavrodiev spec. nov. is available in Fig. S1, the type of *Bactria* O.V. Yurtseva & E.V. Mavrodiev genus novum (*B. ovczinnikovii* (Czhukav.) O.V. Yurtseva & E.V. Mavrodiev) is available in Fig. S2.

### New Species Registration

The following information was supplied regarding the registration of a newly described species: *Bactria* O.V. Yurtseva & E.V. Mavrodiev spec. nov.: *Bactria* 77154106-1. *Bactria ovczinnikovii* (Czukav.) O.V. Yurtseva & E.V. Mavrodiev. comb. nov.: *Bactria ovczinnikovii* 77154107-1. *Bactria lazkovii* O.V. Yurtseva & E.V. Mavrodiev spec. nov.: *Bactria lazkovii* 77154108-1.
