# Supplementary material for: What is Atraphaxis L. (Polygonaceae, Polygoneae): cryptic taxa and resolved taxonomic complexity instead of the formal lumping and the lack of morphological synapomorphies
Source: PeerJ. 2016 May 3;4:e1977. doi: 10.7717/peerj.1977 (PMC4860328; doi:10.7717/peerj.1977)
Supplement: Supplemental Information 1 [file peerj-04-1977-s001.doc]

**Table S1**. **Taxonomic history of position of the genus *Atraphaxis* L. in Polygonoideae (Polygonaceae)**

| Tribes | Polygoneae Rchb. | Atraphaxideae Dummer | Calligoneae  C.A. Mey. | Rumiceae Dumort. | Rhabarbareae Meisn. |
| --- | --- | --- | --- | --- | --- |
| Endlicher 1836-1840 | ***Atraphaxis* L.**  *Calligonum* L.  *Ceratogonum* Meisn.  *Coccoloba* P. Browne  *Fagopyrum* Mill.  *Koenigia* L.  *Polygonum* L. s.lat.  *Emex* Neck. ex Campd.  *Oxygonum* Burch.  *Oxyria* Hill.  *Podopterus* Bonpl.  *Rheum* L.  *Rumex* L.  ***Tragopyrum* M.Bieb.**  *Triplaris* Loefl. |  |  |  |  |
| Ledebour 1847-1849 | (As subtribe “Eupolygoneae”):  *Fagopyrum*  *Polygonum* L.s.lat. (incl. Aconogonon (Meisn.)Rchb., *Avicularia* Meisn., *Bistorta* (L.)Mill., *Persicaria* (L.)Mill., *Tiniaria* (Meisn.)Rchb.)  *Koenigia* |  | *Calligonum*  *Calliphysa* Fischer & C.A.Mey.  *Pterococcus* Pall. | ***Atraphaxis*** *Oxyri*a  *Rheum*  *Rumex*  ***Tragopyrum*** |  |
| Meisner 1857 | (As subtribe “Eupolygoneae”):  *Fagopyrum*  *Koenigia*  *Polygonum* L. s.lat.(incl. *Aconogonon, Bistorta, Persicaria*) |  | *Calligonum*  *Callyphysa*  *Pterococcus* | ***Atraphaxis***  *Polygonella* Michx.  *Thysanella* Salisb.  *Rumex* | *Oxygonum*  *Oxyria*  *Pteropyrum* Jaub. et Spach  *Rheum* |
| Bentham & Hooker 1880 | ***Atraphaxis***  *Fagopyrum*  *Polygonella*  *Polygonum* L. s.str.  *Calligonum*  *Pteropyrum*  *Oxygonum* |  |  |  |  |
| Dammer 1893 | *Fagopyrum*  *Oxygonum*  *Polygonella*  *Polygonum* L. s.str. | ***Atraphaxis*** *Calligonum*  *Pteropyrum* Jaub. et Spach |  | *Emex*  *Oxyria*  *Rheum*  *Rumex* |  |
| Perdrigeat 1900 | F*agopyrum*  *Ceratogonum*  *Oxygonum*  *Polygonella*  *Polygonum* |  | ***Atraphaxis***  *Calligonum*  *Pteropyrum*  *Pterococcus* | *Emex*  *Oxyria*  *Rheum*  *Rumex*  *Koenigia*  *Eriogonum* and others |  |
| Gross 1913 | *Oxygoninae:*  *Oxygonum*  *Atraphaxidinae:*  ***Atraphaxis***  *Calligonum*  *Pteropyrum*  *Polygoninae:*  *Bistorta*  *Fagopyrum*  *Koenigia*  *Persicaria*  *Polygonella*  *Polygonum* *Pleuropterus* Turcz.  *Pteroxygonum* Dammer & Diels.  *Thysanella* Salisb. |  |  | *Emex*  *Oxyria*  *Rheum*  *Rumex*. |  |
| Jaretsky 1925 | ***Atraphaxis***  *Bistorta*  *Calligonum*  *Fagopyrum*  *Koenigia*  *Oxygonum*  *Persicaria*  *Pleuropterus*  *Polygonella*  *Polygonum*  *Pteropyrum*  *Pteroxygonum*  *Thysanella* |  |  | *Emex*  *Oxyria*  *Rheum*  *Rumex*. |  |
| Haraldson 1978 | ***Atraphaxis***  *Calligonum*  *Fagopyrum* (incl. *Pteroxygonum*)  *Oxygonum*  *Polygonella*  *Polygonum* L. s.str.  *Pteropyrum* |  |  | *Emex*  *Oxyria*  *Rheum*  *Rumex*. |  |
| Ronse De Craene & Akeroyd 1988 | ***Atraphaxis***  *Calligonum*  *Fallopia* Adans. (incl. *Reynoutria* Houtt.)  *Oxygonum*  *Polygonella*  *Polygonum*  ***Pteropyrum*** |  |  |  |  |
| Brandbyge 1993 | ***Atraphaxis***  *Calligonum*  *Fallopia*  *Oxygonum*  *Polygonella*  *Polygonum* L. s.str.  *Pteropyrum*  *Reynoutria* |  |  | *Emex*  *Oxyria*  *Rheum*  *Rumex*. |  |
| Takhtajan 2009 | *Cephalophilon* (Meisn.) Börner  *Chylocalyx* Hassk.  *Fallopia*  *Knorringia* (Czukav.) Tzvelev  *Oxygonum*  *Polygonella*  *Polygonum* L.s.str.  *Reynoutria*  *Truellum* Houtt. | ***Atraphaxis***  *Calligonum*  *Parapteropyrum* A. J. Li  *Physopyrum* Popov  *Pteropyrum* |  | *Emex*  *Oxyria*  *Rheum*  *Rumex*. |  |
| Galasso et al. 2009 | ***Atraphaxis***  *Calligonum*  *Fallopia*  ***Homalocladium*** L. H. Bailey  *Muehlenbeckia* Meisn.  *Knorringia*  *Oxygonum*  *Parapteropyrum*  *Polygonum* L. s. str.  (incl. *Polygonella*)  *Pteropyrum*  *Reynoutria*  × *Reyllopia* Holub. |  |  | *Emex*  *Oxyria*  *Rheum*  *Rumex*. |  |
| Schuster, Wilson & Kron, 2011; Schuster, Reveal & Kron, 2011 | ***Atraphaxis***  *Duma* T.M.Schust.  *Fallopia*  *Knorringia*  *Muehlenbeckia* (incl. *Homalocladium*)  *Polygonum* L.s.str. (incl. *Polygonella*)  *Reynoutria* |  | *Calligonum*  *Pteropyrum* | *Emex*  *Oxyria*  *Rheum*  *Rumex*. |  |

Further reading

Dammer U. 1893.Polygonaceae. In: Engler A, Prantl K, eds.Die Natu¨rlichen Pflanzenfamilien,

vol. 3(1, A). Leipzig: W. Engelmann, 1–36.

Endlicher S. 1836–1840.Genera Plantarum Secundum Ordines Naturales Disposita. Vindobonae:

Fr. Beck Univ. Bibliopolam, 1–1483.

Jaretzky R. 1925.Beitra¨ge zur systematik der polygonaceae unter beru ¨cksichtigung des oxymethylanthrachinon-vorkommens: (Contributions to the systematics of thePolygonaceaewith

consideration of the oxymethyl-anthraquinone-occurrence).Feddes Repertorium Specierum

Novarum Regni Vegetabilis22(4–12):49–83DOI 10.1002/fedr.v22:4/12.

Ledebour CFr. 1847–1849.Flora Rossica,vol. 3. Stuttgart: E. Schwiezerbart, 1–866

Perdrigeat MC-A. 1900.Anatomie compare´e des polygone´es et ses rapports avec la morphologie

et la classification.Actes de la Socie´te ´Linne´enne de Bordeaux55(5):1–91.

Takhtajan AL. 2009.Flowering Plants. Berlin: Springer, 1–871 DOI 10.1007/978-1-4020-9609-9.
