# Supplementary material for: What is Atraphaxis L. (Polygonaceae, Polygoneae): cryptic taxa and resolved taxonomic complexity instead of the formal lumping and the lack of morphological synapomorphies
Source: PeerJ. 2016 May 3;4:e1977. doi: 10.7717/peerj.1977 (PMC4860328; doi:10.7717/peerj.1977)
Supplement: Supplemental Information 2 [file peerj-04-1977-s002.doc]

**Table S2. Classification history of the genus *Atraphaxis* L.**

| Linnaeus, 1753 | Marschall Bieberstein,  1808, 1819 | Jaubert & Spach, 1844–1846 | Meisner, 1857 | Boissier, 1879 | Krasnov, 1888 | Pavlov, 1936 | Lovelius,  1979 |
| --- | --- | --- | --- | --- | --- | --- | --- |
| *Atraphaxis* L:  P_4_A_6_G_(2)_ | *Atraphaxis* L. (1808) | *Atraphaxis* subgen. *Euatraphaxis* Jaub. & Spach | *Atraphaxis* sect. *Euatraphaxis* Jaub. & Spach | *Atraphaxis* sect. *Euatraphaxis* Jaub. & Spach | *Atraphaxis* sect. *Euatraphaxis* Meisn. | *Atraphaxis* subgen.  *Euatraphaxis* Jaub. & Spach | *Atraphaxis* sect. *Atraphaxis* |
| P_5_A_8_G_(3)_ | *Tragopyrum* M.Bieb. (1819) | *Atraphaxis* subgen. *Tragopyrum* (M.Bieb.) Jaub. & Spach | *Atraphaxis* sect. *Tragopyrum* (M.Bieb.) Meisn. | *Atraphaxis* sect. *Tragopyrum* (M.Bieb.) Meisn. | *Atraphaxis* sect. *Tragopyrum* (M.Bieb.) Meisn. | *Atraphaxis* subgen. *Tragopyrum* (M.Bieb.) Jaub. & Spach | *Atraphaxis* sect. *Tragopyrum* (M.Bieb.) Meisn. |
|  |  |  |  |  | *Atraphaxis* sect. *Protatraphaxis* Krasn. |  |  |
|  |  |  |  |  | *Atraphaxis* sect. *Atraphaxis* Krasn. |  |  |
|  |  | *Atraphaxis* subgen. *Tragatraphaxis* Jaub. & Spach | *Atraphaxis* sect. *Tragatraphaxis* Jaub. & Spach |  | *Atraphaxis* sect. *Tragatraphaxis (*Jaub. & Spach) Krasn. |  |  |
|  |  |  |  |  |  |  | *Atraphaxis* sect. *Physopyrum* (Popov) Lovelius |

Further reading:

Marschall Bieberstein LBFr. 1808. Flora Taurico-Caucasica 1. Charkov: Typis Academicis 1–428.
