# Supplementary material for: What is Atraphaxis L. (Polygonaceae, Polygoneae): cryptic taxa and resolved taxonomic complexity instead of the formal lumping and the lack of morphological synapomorphies
Source: PeerJ. 2016 May 3;4:e1977. doi: 10.7717/peerj.1977 (PMC4860328; doi:10.7717/peerj.1977)
Supplement: Supplemental Information 3 [file peerj-04-1977-s003.doc]

**Table S3. Taxa, voucher information, current and GenBank accession numbers used in the study**.

| Taxon | Source, voucher | Cur-rent number | ITS | *rpl*32-*trn*L^(UAG)^ | *trn*L-*trn*F |
| --- | --- | --- | --- | --- | --- |
| *Atraphaxis* *angustifolia* [Jaub.](http://www.ipni.org/ipni/idAuthorSearch.do?id=4441-1&back_page=%2Fipni%2FeditAdvPlantNameSearch.do%3Bjsessionid%3DCD5B69CD9903C9DD70928476C2EC3D1F%3Ffind_infragenus%3D%26find_isAPNIRecord%3Dtrue%26find_geoUnit%3D%26find_includePublicationAuthors%3Dtrue%26find_addedSince%3D%26find_family%3D%26find_genus%3DAtraphaxis%26find_sortByFamily%3Dtrue%26find_isGCIRecord%3Dtrue%26find_infrafamily%3D%26find_rankToReturn%3Dall%26find_publicationTitle%3D%26find_authorAbbrev%3D%26find_infraspecies%3D%26find_includeBasionymAuthors%3Dtrue%26find_modifiedSince%3D%26find_isIKRecord%3Dtrue%26find_species%3D%26output_format%3Dnormal) & [Spach](http://www.ipni.org/ipni/idAuthorSearch.do?id=9939-1&back_page=%2Fipni%2FeditAdvPlantNameSearch.do%3Bjsessionid%3DCD5B69CD9903C9DD70928476C2EC3D1F%3Ffind_infragenus%3D%26find_isAPNIRecord%3Dtrue%26find_geoUnit%3D%26find_includePublicationAuthors%3Dtrue%26find_addedSince%3D%26find_family%3D%26find_genus%3DAtraphaxis%26find_sortByFamily%3Dtrue%26find_isGCIRecord%3Dtrue%26find_infrafamily%3D%26find_rankToReturn%3Dall%26find_publicationTitle%3D%26find_authorAbbrev%3D%26find_infraspecies%3D%26find_includeBasionymAuthors%3Dtrue%26find_modifiedSince%3D%26find_isIKRecord%3Dtrue%26find_species%3D%26output_format%3Dnormal) | Armenia, Megri d. Zangezur Ridge. 1.06.1973. *Shvedchikova* (MW) | 4 | **KJ707454** | **KU724453** | **KU508756** |
| *A. ariana* (Grigorj.) T.M. Schust. & Reveal | Turkmenistan, [Badghys], v. Morgunovsky. 25.04.1988. *Gorelova* (LE) | 122 | GQ339970 | **KU724454** | **KU508757** |
| *A. atraphaxiformis* (Botsch.) T.M. Schust. & Reveal | Kyrgyzstan, Alay Ridge, Kadamzhay. 19.07.2005. Lazkov (FRU)  Yurtseva et al. 2010 | 41 | GQ339980 | **KU724455** | **KU508758** |
| *A. atraphaxiformis* | Uzbekistan, Alay, Turkestan Ridge, the Isphara. 07.1970. *Kamelin 532* (LE) | 43 | GQ339981 | **KU724456** | **KU508759** |
| *A. avenia* Botsch. | Kyrgyzstan, Alay Ridge, the Gulcha basin, Irgailysu, Sufi-Kurgan. 16.07.1987. *Pimenov, Klujkov 407*. (MW) | 58 | **KJ707526** | **KU724457** | **KJ690694** |
| *A.* *aucherii* [Jaub.](http://www.ipni.org/ipni/idAuthorSearch.do?id=4441-1&back_page=%2Fipni%2FeditAdvPlantNameSearch.do%3Bjsessionid%3DCD5B69CD9903C9DD70928476C2EC3D1F%3Ffind_infragenus%3D%26find_isAPNIRecord%3Dtrue%26find_geoUnit%3D%26find_includePublicationAuthors%3Dtrue%26find_addedSince%3D%26find_family%3D%26find_genus%3DAtraphaxis%26find_sortByFamily%3Dtrue%26find_isGCIRecord%3Dtrue%26find_infrafamily%3D%26find_rankToReturn%3Dall%26find_publicationTitle%3D%26find_authorAbbrev%3D%26find_infraspecies%3D%26find_includeBasionymAuthors%3Dtrue%26find_modifiedSince%3D%26find_isIKRecord%3Dtrue%26find_species%3D%26output_format%3Dnormal) & [Spach](http://www.ipni.org/ipni/idAuthorSearch.do?id=9939-1&back_page=%2Fipni%2FeditAdvPlantNameSearch.do%3Bjsessionid%3DCD5B69CD9903C9DD70928476C2EC3D1F%3Ffind_infragenus%3D%26find_isAPNIRecord%3Dtrue%26find_geoUnit%3D%26find_includePublicationAuthors%3Dtrue%26find_addedSince%3D%26find_family%3D%26find_genus%3DAtraphaxis%26find_sortByFamily%3Dtrue%26find_isGCIRecord%3Dtrue%26find_infrafamily%3D%26find_rankToReturn%3Dall%26find_publicationTitle%3D%26find_authorAbbrev%3D%26find_infraspecies%3D%26find_includeBasionymAuthors%3Dtrue%26find_modifiedSince%3D%26find_isIKRecord%3Dtrue%26find_species%3D%26output_format%3Dnormal) | Tavakkoli et al. 2015 |  | AB976642* | AB976694* | — |
| *A. badghysi* Kult. | Turkmenistan, Er Oylan-Duz. 21.04.1965. *Meschcheryakov* (LE) | 13 | **KJ707455** | **KU724458** | **KU508760** |
| *A._billardierei* Jaub. & Spach | Schuster et al. 2011b |  | JN161130* | — | — |
| *A. binaludensis* [S.Tavakkoli](http://www.ipni.org/ipni/idAuthorSearch.do?id=20024887-1&back_page=%2Fipni%2FeditAdvPlantNameSearch.do%3Bjsessionid%3DCD5B69CD9903C9DD70928476C2EC3D1F%3Ffind_infragenus%3D%26find_isAPNIRecord%3Dtrue%26find_geoUnit%3D%26find_includePublicationAuthors%3Dtrue%26find_addedSince%3D%26find_family%3D%26find_genus%3DAtraphaxis%26find_sortByFamily%3Dtrue%26find_isGCIRecord%3Dtrue%26find_infrafamily%3D%26find_rankToReturn%3Dall%26find_publicationTitle%3D%26find_authorAbbrev%3D%26find_infraspecies%3D%26find_includeBasionymAuthors%3Dtrue%26find_modifiedSince%3D%26find_isIKRecord%3Dtrue%26find_species%3D%26output_format%3Dnormal), [Mozaff.](http://www.ipni.org/ipni/idAuthorSearch.do?id=23696-1&back_page=%2Fipni%2FeditAdvPlantNameSearch.do%3Bjsessionid%3DCD5B69CD9903C9DD70928476C2EC3D1F%3Ffind_infragenus%3D%26find_isAPNIRecord%3Dtrue%26find_geoUnit%3D%26find_includePublicationAuthors%3Dtrue%26find_addedSince%3D%26find_family%3D%26find_genus%3DAtraphaxis%26find_sortByFamily%3Dtrue%26find_isGCIRecord%3Dtrue%26find_infrafamily%3D%26find_rankToReturn%3Dall%26find_publicationTitle%3D%26find_authorAbbrev%3D%26find_infraspecies%3D%26find_includeBasionymAuthors%3Dtrue%26find_modifiedSince%3D%26find_isIKRecord%3Dtrue%26find_species%3D%26output_format%3Dnormal) & [Kaz. Osaloo](http://www.ipni.org/ipni/idAuthorSearch.do?id=20000636-2&back_page=%2Fipni%2FeditAdvPlantNameSearch.do%3Bjsessionid%3DCD5B69CD9903C9DD70928476C2EC3D1F%3Ffind_infragenus%3D%26find_isAPNIRecord%3Dtrue%26find_geoUnit%3D%26find_includePublicationAuthors%3Dtrue%26find_addedSince%3D%26find_family%3D%26find_genus%3DAtraphaxis%26find_sortByFamily%3Dtrue%26find_isGCIRecord%3Dtrue%26find_infrafamily%3D%26find_rankToReturn%3Dall%26find_publicationTitle%3D%26find_authorAbbrev%3D%26find_infraspecies%3D%26find_includeBasionymAuthors%3Dtrue%26find_modifiedSince%3D%26find_isIKRecord%3Dtrue%26find_species%3D%26output_format%3Dnormal) | Tavakkoli et al. 2015 |  | AB976643* | AB976695* | — |
| *A. bracteata* Losinsk. | China, Inner Mongolia, Ikodzhoumen. 10.08.1957. *M.P.Petrow* (MW) | 157 | **KU508742** | **KU724459** | **KU508761** |
| *A.* *grandiflora* [Jaub.](http://www.ipni.org/ipni/idAuthorSearch.do?id=4441-1&back_page=%2Fipni%2FeditAdvPlantNameSearch.do%3Bjsessionid%3DCD5B69CD9903C9DD70928476C2EC3D1F%3Ffind_infragenus%3D%26find_isAPNIRecord%3Dtrue%26find_geoUnit%3D%26find_includePublicationAuthors%3Dtrue%26find_addedSince%3D%26find_family%3D%26find_genus%3DAtraphaxis%26find_sortByFamily%3Dtrue%26find_isGCIRecord%3Dtrue%26find_infrafamily%3D%26find_rankToReturn%3Dall%26find_publicationTitle%3D%26find_authorAbbrev%3D%26find_infraspecies%3D%26find_includeBasionymAuthors%3Dtrue%26find_modifiedSince%3D%26find_isIKRecord%3Dtrue%26find_species%3D%26output_format%3Dnormal) & [Spach](http://www.ipni.org/ipni/idAuthorSearch.do?id=9939-1&back_page=%2Fipni%2FeditAdvPlantNameSearch.do%3Bjsessionid%3DCD5B69CD9903C9DD70928476C2EC3D1F%3Ffind_infragenus%3D%26find_isAPNIRecord%3Dtrue%26find_geoUnit%3D%26find_includePublicationAuthors%3Dtrue%26find_addedSince%3D%26find_family%3D%26find_genus%3DAtraphaxis%26find_sortByFamily%3Dtrue%26find_isGCIRecord%3Dtrue%26find_infrafamily%3D%26find_rankToReturn%3Dall%26find_publicationTitle%3D%26find_authorAbbrev%3D%26find_infraspecies%3D%26find_includeBasionymAuthors%3Dtrue%26find_modifiedSince%3D%26find_isIKRecord%3Dtrue%26find_species%3D%26output_format%3Dnormal) | Turkey, Cappadocia, Aslan-Dach (Anti-Taurus), Tesaree. 7.08.1856. *Balansa* (LE). | 131 | **KU508743** | — | — |
| *A. fischeri* [Jaub.](http://www.ipni.org/ipni/idAuthorSearch.do?id=4441-1&back_page=%2Fipni%2FeditAdvPlantNameSearch.do%3Bjsessionid%3DCD5B69CD9903C9DD70928476C2EC3D1F%3Ffind_infragenus%3D%26find_isAPNIRecord%3Dtrue%26find_geoUnit%3D%26find_includePublicationAuthors%3Dtrue%26find_addedSince%3D%26find_family%3D%26find_genus%3DAtraphaxis%26find_sortByFamily%3Dtrue%26find_isGCIRecord%3Dtrue%26find_infrafamily%3D%26find_rankToReturn%3Dall%26find_publicationTitle%3D%26find_authorAbbrev%3D%26find_infraspecies%3D%26find_includeBasionymAuthors%3Dtrue%26find_modifiedSince%3D%26find_isIKRecord%3Dtrue%26find_species%3D%26output_format%3Dnormal) & [Spach](http://www.ipni.org/ipni/idAuthorSearch.do?id=9939-1&back_page=%2Fipni%2FeditAdvPlantNameSearch.do%3Bjsessionid%3DCD5B69CD9903C9DD70928476C2EC3D1F%3Ffind_infragenus%3D%26find_isAPNIRecord%3Dtrue%26find_geoUnit%3D%26find_includePublicationAuthors%3Dtrue%26find_addedSince%3D%26find_family%3D%26find_genus%3DAtraphaxis%26find_sortByFamily%3Dtrue%26find_isGCIRecord%3Dtrue%26find_infrafamily%3D%26find_rankToReturn%3Dall%26find_publicationTitle%3D%26find_authorAbbrev%3D%26find_infraspecies%3D%26find_includeBasionymAuthors%3Dtrue%26find_modifiedSince%3D%26find_isIKRecord%3Dtrue%26find_species%3D%26output_format%3Dnormal) | West Kazakhstan, Atyrau reg. Inder. 04.05.2011. *Onipchenko* (MW) | 33 | **KJ707528** | **KU724460** | **KJ690713** |
| *A.* *frutescens* ([L.](http://www.ipni.org/ipni/idAuthorSearch.do?id=12653-1&back_page=%2Fipni%2FeditAdvPlantNameSearch.do%3Bjsessionid%3DCD5B69CD9903C9DD70928476C2EC3D1F%3Ffind_infragenus%3D%26find_isAPNIRecord%3Dtrue%26find_geoUnit%3D%26find_includePublicationAuthors%3Dtrue%26find_addedSince%3D%26find_family%3D%26find_genus%3DAtraphaxis%26find_sortByFamily%3Dtrue%26find_isGCIRecord%3Dtrue%26find_infrafamily%3D%26find_rankToReturn%3Dall%26find_publicationTitle%3D%26find_authorAbbrev%3D%26find_infraspecies%3D%26find_includeBasionymAuthors%3Dtrue%26find_modifiedSince%3D%26find_isIKRecord%3Dtrue%26find_species%3D%26output_format%3Dnormal)) [K.Koch](http://www.ipni.org/ipni/idAuthorSearch.do?id=4899-1&back_page=%2Fipni%2FeditAdvPlantNameSearch.do%3Bjsessionid%3DCD5B69CD9903C9DD70928476C2EC3D1F%3Ffind_infragenus%3D%26find_isAPNIRecord%3Dtrue%26find_geoUnit%3D%26find_includePublicationAuthors%3Dtrue%26find_addedSince%3D%26find_family%3D%26find_genus%3DAtraphaxis%26find_sortByFamily%3Dtrue%26find_isGCIRecord%3Dtrue%26find_infrafamily%3D%26find_rankToReturn%3Dall%26find_publicationTitle%3D%26find_authorAbbrev%3D%26find_infraspecies%3D%26find_includeBasionymAuthors%3Dtrue%26find_modifiedSince%3D%26find_isIKRecord%3Dtrue%26find_species%3D%26output_format%3Dnormal) | China, Xinjiang. 2010. *Olonova* (MW) | 67 | **KJ707478** | **KU724461** | **KJ690702** |
| *A.* *intricata* [Mozaff.](http://www.ipni.org/ipni/idAuthorSearch.do?id=23696-1&back_page=%2Fipni%2FeditAdvPlantNameSearch.do%3Bjsessionid%3DCD5B69CD9903C9DD70928476C2EC3D1F%3Ffind_infragenus%3D%26find_isAPNIRecord%3Dtrue%26find_geoUnit%3D%26find_includePublicationAuthors%3Dtrue%26find_addedSince%3D%26find_family%3D%26find_genus%3DAtraphaxis%26find_sortByFamily%3Dtrue%26find_isGCIRecord%3Dtrue%26find_infrafamily%3D%26find_rankToReturn%3Dall%26find_publicationTitle%3D%26find_authorAbbrev%3D%26find_infraspecies%3D%26find_includeBasionymAuthors%3Dtrue%26find_modifiedSince%3D%26find_isIKRecord%3Dtrue%26find_species%3D%26output_format%3Dnormal) | Tavakkoli et al. 2015 |  | AB976646* | AB976698* | — |
| *Atraphaxis* *karataviensis* Pavlov & [Lipsch.](http://www.ipni.org/ipni/idAuthorSearch.do?id=12655-1&back_page=%2Fipni%2FeditAdvPlantNameSearch.do%3Bjsessionid%3DCD5B69CD9903C9DD70928476C2EC3D1F%3Ffind_infragenus%3D%26find_isAPNIRecord%3Dtrue%26find_geoUnit%3D%26find_includePublicationAuthors%3Dtrue%26find_addedSince%3D%26find_family%3D%26find_genus%3DAtraphaxis%26find_sortByFamily%3Dtrue%26find_isGCIRecord%3Dtrue%26find_infrafamily%3D%26find_rankToReturn%3Dall%26find_publicationTitle%3D%26find_authorAbbrev%3D%26find_infraspecies%3D%26find_includeBasionymAuthors%3Dtrue%26find_modifiedSince%3D%26find_isIKRecord%3Dtrue%26find_species%3D%26output_format%3Dnormal) | Kyrgyzstan, Ichkem-Tau Mts., Orlovka. 07.1973. *Kamelin* (LE) | 100 | **KJ707494** | **KU724462** | **KU508762** |
| *A.* *kopetdagensis* [Kovalevsk.](http://www.ipni.org/ipni/idAuthorSearch.do?id=4994-1&back_page=%2Fipni%2FeditAdvPlantNameSearch.do%3Bjsessionid%3DCD5B69CD9903C9DD70928476C2EC3D1F%3Ffind_infragenus%3D%26find_isAPNIRecord%3Dtrue%26find_geoUnit%3D%26find_includePublicationAuthors%3Dtrue%26find_addedSince%3D%26find_family%3D%26find_genus%3DAtraphaxis%26find_sortByFamily%3Dtrue%26find_isGCIRecord%3Dtrue%26find_infrafamily%3D%26find_rankToReturn%3Dall%26find_publicationTitle%3D%26find_authorAbbrev%3D%26find_infraspecies%3D%26find_includeBasionymAuthors%3Dtrue%26find_modifiedSince%3D%26find_isIKRecord%3Dtrue%26find_species%3D%26output_format%3Dnormal) | Turkmenistan, Kazandzhik d. Trgoy Mts. 11-15.05.1981. *Proskuryakova* (MHA) | 133 | **KU508744** | **KU724463** | **KU508763** |
| *A.* *laetevirens* [Jaub.](http://www.ipni.org/ipni/idAuthorSearch.do?id=4441-1&back_page=%2Fipni%2FeditAdvPlantNameSearch.do%3Bjsessionid%3DCD5B69CD9903C9DD70928476C2EC3D1F%3Ffind_infragenus%3D%26find_isAPNIRecord%3Dtrue%26find_geoUnit%3D%26find_includePublicationAuthors%3Dtrue%26find_addedSince%3D%26find_family%3D%26find_genus%3DAtraphaxis%26find_sortByFamily%3Dtrue%26find_isGCIRecord%3Dtrue%26find_infrafamily%3D%26find_rankToReturn%3Dall%26find_publicationTitle%3D%26find_authorAbbrev%3D%26find_infraspecies%3D%26find_includeBasionymAuthors%3Dtrue%26find_modifiedSince%3D%26find_isIKRecord%3Dtrue%26find_species%3D%26output_format%3Dnormal) & [Spach](http://www.ipni.org/ipni/idAuthorSearch.do?id=9939-1&back_page=%2Fipni%2FeditAdvPlantNameSearch.do%3Bjsessionid%3DCD5B69CD9903C9DD70928476C2EC3D1F%3Ffind_infragenus%3D%26find_isAPNIRecord%3Dtrue%26find_geoUnit%3D%26find_includePublicationAuthors%3Dtrue%26find_addedSince%3D%26find_family%3D%26find_genus%3DAtraphaxis%26find_sortByFamily%3Dtrue%26find_isGCIRecord%3Dtrue%26find_infrafamily%3D%26find_rankToReturn%3Dall%26find_publicationTitle%3D%26find_authorAbbrev%3D%26find_infraspecies%3D%26find_includeBasionymAuthors%3Dtrue%26find_modifiedSince%3D%26find_isIKRecord%3Dtrue%26find_species%3D%26output_format%3Dnormal) | Kazakhstan, Dzhungar Alatau, Kaikan Mts, Glinovka. 18.06.1959. *Goloskokov* (MW) | 109 | **KJ707497** | **KU724464** | **KU508764** |
| *A.* *muschketowi* [Krasn.](http://www.ipni.org/ipni/idAuthorSearch.do?id=5030-1&back_page=%2Fipni%2FeditAdvPlantNameSearch.do%3Bjsessionid%3DCD5B69CD9903C9DD70928476C2EC3D1F%3Ffind_infragenus%3D%26find_isAPNIRecord%3Dtrue%26find_geoUnit%3D%26find_includePublicationAuthors%3Dtrue%26find_addedSince%3D%26find_family%3D%26find_genus%3DAtraphaxis%26find_sortByFamily%3Dtrue%26find_isGCIRecord%3Dtrue%26find_infrafamily%3D%26find_rankToReturn%3Dall%26find_publicationTitle%3D%26find_authorAbbrev%3D%26find_infraspecies%3D%26find_includeBasionymAuthors%3Dtrue%26find_modifiedSince%3D%26find_isIKRecord%3Dtrue%26find_species%3D%26output_format%3Dnormal) | Kazakhstan, Tian Shan, Zaily Alatau, Alma-Ata d., Mt. Kok-Tebe. 24.05.1998. *Majorov 98-24в*. (MW) | 41 | **KJ707502** | **KU724465** | **KJ690697** |
| *A.* *pungens* [Jaub.](http://www.ipni.org/ipni/idAuthorSearch.do?id=4441-1&back_page=%2Fipni%2FeditAdvPlantNameSearch.do%3Bjsessionid%3DCD5B69CD9903C9DD70928476C2EC3D1F%3Ffind_infragenus%3D%26find_isAPNIRecord%3Dtrue%26find_geoUnit%3D%26find_includePublicationAuthors%3Dtrue%26find_addedSince%3D%26find_family%3D%26find_genus%3DAtraphaxis%26find_sortByFamily%3Dtrue%26find_isGCIRecord%3Dtrue%26find_infrafamily%3D%26find_rankToReturn%3Dall%26find_publicationTitle%3D%26find_authorAbbrev%3D%26find_infraspecies%3D%26find_includeBasionymAuthors%3Dtrue%26find_modifiedSince%3D%26find_isIKRecord%3Dtrue%26find_species%3D%26output_format%3Dnormal) & [Spach](http://www.ipni.org/ipni/idAuthorSearch.do?id=9939-1&back_page=%2Fipni%2FeditAdvPlantNameSearch.do%3Bjsessionid%3DCD5B69CD9903C9DD70928476C2EC3D1F%3Ffind_infragenus%3D%26find_isAPNIRecord%3Dtrue%26find_geoUnit%3D%26find_includePublicationAuthors%3Dtrue%26find_addedSince%3D%26find_family%3D%26find_genus%3DAtraphaxis%26find_sortByFamily%3Dtrue%26find_isGCIRecord%3Dtrue%26find_infrafamily%3D%26find_rankToReturn%3Dall%26find_publicationTitle%3D%26find_authorAbbrev%3D%26find_infraspecies%3D%26find_includeBasionymAuthors%3Dtrue%26find_modifiedSince%3D%26find_isIKRecord%3Dtrue%26find_species%3D%26output_format%3Dnormal) | Mongolia, Central aimag, Telengyim-Baishin, Mt. Dzamryn-Ula. 23.06.1988. *Budantsev et al. 27.* (MW) | 136 | **KU508745** | **KU724466** | **KU508765** |
| *A.* *pyrifolia* [Bunge](http://www.ipni.org/ipni/idAuthorSearch.do?id=12367-1&back_page=%2Fipni%2FeditAdvPlantNameSearch.do%3Bjsessionid%3DCD5B69CD9903C9DD70928476C2EC3D1F%3Ffind_infragenus%3D%26find_isAPNIRecord%3Dtrue%26find_geoUnit%3D%26find_includePublicationAuthors%3Dtrue%26find_addedSince%3D%26find_family%3D%26find_genus%3DAtraphaxis%26find_sortByFamily%3Dtrue%26find_isGCIRecord%3Dtrue%26find_infrafamily%3D%26find_rankToReturn%3Dall%26find_publicationTitle%3D%26find_authorAbbrev%3D%26find_infraspecies%3D%26find_includeBasionymAuthors%3Dtrue%26find_modifiedSince%3D%26find_isIKRecord%3Dtrue%26find_species%3D%26output_format%3Dnormal) | Tadjikistan, Badakhshan, Schugnan d. the Gunt, Vozh × Shtamm. 02.08.2011. *Klujkov et al. 26.* (MW) | 36 | **KJ707505** | **KU724467** | **KJ690696** |
| *A.* *radkanensis* [S.Tavakkoli](http://www.ipni.org/ipni/idAuthorSearch.do?id=20024887-1&back_page=%2Fipni%2FeditAdvPlantNameSearch.do%3Bjsessionid%3DCD5B69CD9903C9DD70928476C2EC3D1F%3Ffind_infragenus%3D%26find_isAPNIRecord%3Dtrue%26find_geoUnit%3D%26find_includePublicationAuthors%3Dtrue%26find_addedSince%3D%26find_family%3D%26find_genus%3DAtraphaxis%26find_sortByFamily%3Dtrue%26find_isGCIRecord%3Dtrue%26find_infrafamily%3D%26find_rankToReturn%3Dall%26find_publicationTitle%3D%26find_authorAbbrev%3D%26find_infraspecies%3D%26find_includeBasionymAuthors%3Dtrue%26find_modifiedSince%3D%26find_isIKRecord%3Dtrue%26find_species%3D%26output_format%3Dnormal), [Kaz.Osaloo](http://www.ipni.org/ipni/idAuthorSearch.do?id=20000636-2&back_page=%2Fipni%2FeditAdvPlantNameSearch.do%3Bjsessionid%3DCD5B69CD9903C9DD70928476C2EC3D1F%3Ffind_infragenus%3D%26find_isAPNIRecord%3Dtrue%26find_geoUnit%3D%26find_includePublicationAuthors%3Dtrue%26find_addedSince%3D%26find_family%3D%26find_genus%3DAtraphaxis%26find_sortByFamily%3Dtrue%26find_isGCIRecord%3Dtrue%26find_infrafamily%3D%26find_rankToReturn%3Dall%26find_publicationTitle%3D%26find_authorAbbrev%3D%26find_infraspecies%3D%26find_includeBasionymAuthors%3Dtrue%26find_modifiedSince%3D%26find_isIKRecord%3Dtrue%26find_species%3D%26output_format%3Dnormal) & [Mozaff.](http://www.ipni.org/ipni/idAuthorSearch.do?id=23696-1&back_page=%2Fipni%2FeditAdvPlantNameSearch.do%3Bjsessionid%3DCD5B69CD9903C9DD70928476C2EC3D1F%3Ffind_infragenus%3D%26find_isAPNIRecord%3Dtrue%26find_geoUnit%3D%26find_includePublicationAuthors%3Dtrue%26find_addedSince%3D%26find_family%3D%26find_genus%3DAtraphaxis%26find_sortByFamily%3Dtrue%26find_isGCIRecord%3Dtrue%26find_infrafamily%3D%26find_rankToReturn%3Dall%26find_publicationTitle%3D%26find_authorAbbrev%3D%26find_infraspecies%3D%26find_includeBasionymAuthors%3Dtrue%26find_modifiedSince%3D%26find_isIKRecord%3Dtrue%26find_species%3D%26output_format%3Dnormal) | Tavakkoli et al. 2015 |  | AB976649* | AB976701* | — |
| *A.* *replicata* [Lam.](http://www.ipni.org/ipni/idAuthorSearch.do?id=5227-1&back_page=%2Fipni%2FeditAdvPlantNameSearch.do%3Bjsessionid%3DCD5B69CD9903C9DD70928476C2EC3D1F%3Ffind_infragenus%3D%26find_isAPNIRecord%3Dtrue%26find_geoUnit%3D%26find_includePublicationAuthors%3Dtrue%26find_addedSince%3D%26find_family%3D%26find_genus%3DAtraphaxis%26find_sortByFamily%3Dtrue%26find_isGCIRecord%3Dtrue%26find_infrafamily%3D%26find_rankToReturn%3Dall%26find_publicationTitle%3D%26find_authorAbbrev%3D%26find_infraspecies%3D%26find_includeBasionymAuthors%3Dtrue%26find_modifiedSince%3D%26find_isIKRecord%3Dtrue%26find_species%3D%26output_format%3Dnormal) | Kazakhstan, Usturt, Mangistau reg., Beyneu. 3.06.2003. *Sukhorukov* (MW) | 24 | **KJ707516** | **KU724468** | **KJ690714** |
| *A. replicata* Lam. | Kyrgyzstan, Alay, the Gulcha, Kyzyl-Kurgan. 08.07.1986. *Kuvaev 518-3*. (MW) | 31 | **KJ707514** | **KU724469** | **KJ690716** |
| *A.* *seravschanica* Pavlov | Kyrgyzstan, Chatkal Ridge, Alabuksay, Alabuk. 2.09.1982. *Borodina, Philatova* (LE) | 98 | **KJ707524** | **KU724470** | **KJ690693** |
| *A. spinosa* L. | West Kazakhstan, Mangystau reg. Beyneu. 07.05.2011. *Onipchenko* (MW) | 34 | **KJ707534** | **KU724471** | **KJ690715** |
| *A. spinosa* L. | Armenia, Ararat reg. Gorevan. 13.08.2012. *Lyskov* (MW) | 141 | **KU508746** | **KU724472** | **KU508766** |
| *Atraphaxis* *suaedifolia* [Jaub.](http://www.ipni.org/ipni/idAuthorSearch.do?id=4441-1&back_page=%2Fipni%2FeditAdvPlantNameSearch.do%3Bjsessionid%3DCD5B69CD9903C9DD70928476C2EC3D1F%3Ffind_infragenus%3D%26find_isAPNIRecord%3Dtrue%26find_geoUnit%3D%26find_includePublicationAuthors%3Dtrue%26find_addedSince%3D%26find_family%3D%26find_genus%3DAtraphaxis%26find_sortByFamily%3Dtrue%26find_isGCIRecord%3Dtrue%26find_infrafamily%3D%26find_rankToReturn%3Dall%26find_publicationTitle%3D%26find_authorAbbrev%3D%26find_infraspecies%3D%26find_includeBasionymAuthors%3Dtrue%26find_modifiedSince%3D%26find_isIKRecord%3Dtrue%26find_species%3D%26output_format%3Dnormal) & [Spach](http://www.ipni.org/ipni/idAuthorSearch.do?id=9939-1&back_page=%2Fipni%2FeditAdvPlantNameSearch.do%3Bjsessionid%3DCD5B69CD9903C9DD70928476C2EC3D1F%3Ffind_infragenus%3D%26find_isAPNIRecord%3Dtrue%26find_geoUnit%3D%26find_includePublicationAuthors%3Dtrue%26find_addedSince%3D%26find_family%3D%26find_genus%3DAtraphaxis%26find_sortByFamily%3Dtrue%26find_isGCIRecord%3Dtrue%26find_infrafamily%3D%26find_rankToReturn%3Dall%26find_publicationTitle%3D%26find_authorAbbrev%3D%26find_infraspecies%3D%26find_includeBasionymAuthors%3Dtrue%26find_modifiedSince%3D%26find_isIKRecord%3Dtrue%26find_species%3D%26output_format%3Dnormal) | Tavakkoli et al. 2015 |  | AB542773* | AB976705* | — |
| *A.* *teretifolia* ([Popov](http://www.ipni.org/ipni/idAuthorSearch.do?id=7848-1&back_page=%2Fipni%2FeditAdvPlantNameSearch.do%3Bjsessionid%3DCD5B69CD9903C9DD70928476C2EC3D1F%3Ffind_infragenus%3D%26find_isAPNIRecord%3Dtrue%26find_geoUnit%3D%26find_includePublicationAuthors%3Dtrue%26find_addedSince%3D%26find_family%3D%26find_genus%3DAtraphaxis%26find_sortByFamily%3Dtrue%26find_isGCIRecord%3Dtrue%26find_infrafamily%3D%26find_rankToReturn%3Dall%26find_publicationTitle%3D%26find_authorAbbrev%3D%26find_infraspecies%3D%26find_includeBasionymAuthors%3Dtrue%26find_modifiedSince%3D%26find_isIKRecord%3Dtrue%26find_species%3D%26output_format%3Dnormal)) [Kom.](http://www.ipni.org/ipni/idAuthorSearch.do?id=4948-1&back_page=%2Fipni%2FeditAdvPlantNameSearch.do%3Bjsessionid%3DCD5B69CD9903C9DD70928476C2EC3D1F%3Ffind_infragenus%3D%26find_isAPNIRecord%3Dtrue%26find_geoUnit%3D%26find_includePublicationAuthors%3Dtrue%26find_addedSince%3D%26find_family%3D%26find_genus%3DAtraphaxis%26find_sortByFamily%3Dtrue%26find_isGCIRecord%3Dtrue%26find_infrafamily%3D%26find_rankToReturn%3Dall%26find_publicationTitle%3D%26find_authorAbbrev%3D%26find_infraspecies%3D%26find_includeBasionymAuthors%3Dtrue%26find_modifiedSince%3D%26find_isIKRecord%3Dtrue%26find_species%3D%26output_format%3Dnormal) | Kazakhstan, Karaganda reg., Dzheskazgan × Ula-Tau. 23.06.1958. *Rachkovskaya 6185*. (LE) | 142 | **KU508747** | **KU724473** | **KU508767** |
| *A.* *toktogulica* ([Lazkov](http://www.ipni.org/ipni/idAuthorSearch.do?id=36817-1&back_page=%2Fipni%2FeditAdvPlantNameSearch.do%3Bjsessionid%3DCD5B69CD9903C9DD70928476C2EC3D1F%3Ffind_infragenus%3D%26find_isAPNIRecord%3Dtrue%26find_geoUnit%3D%26find_includePublicationAuthors%3Dtrue%26find_addedSince%3D%26find_family%3D%26find_genus%3DAtraphaxis%26find_sortByFamily%3Dtrue%26find_isGCIRecord%3Dtrue%26find_infrafamily%3D%26find_rankToReturn%3Dall%26find_publicationTitle%3D%26find_authorAbbrev%3D%26find_infraspecies%3D%26find_includeBasionymAuthors%3Dtrue%26find_modifiedSince%3D%26find_isIKRecord%3Dtrue%26find_species%3D%26output_format%3Dnormal)) [T.M.Schust.](http://www.ipni.org/ipni/idAuthorSearch.do?id=20018666-1&back_page=%2Fipni%2FeditAdvPlantNameSearch.do%3Bjsessionid%3DCD5B69CD9903C9DD70928476C2EC3D1F%3Ffind_infragenus%3D%26find_isAPNIRecord%3Dtrue%26find_geoUnit%3D%26find_includePublicationAuthors%3Dtrue%26find_addedSince%3D%26find_family%3D%26find_genus%3DAtraphaxis%26find_sortByFamily%3Dtrue%26find_isGCIRecord%3Dtrue%26find_infrafamily%3D%26find_rankToReturn%3Dall%26find_publicationTitle%3D%26find_authorAbbrev%3D%26find_infraspecies%3D%26find_includeBasionymAuthors%3Dtrue%26find_modifiedSince%3D%26find_isIKRecord%3Dtrue%26find_species%3D%26output_format%3Dnormal) & [Reveal](http://www.ipni.org/ipni/idAuthorSearch.do?id=8314-1&back_page=%2Fipni%2FeditAdvPlantNameSearch.do%3Bjsessionid%3DCD5B69CD9903C9DD70928476C2EC3D1F%3Ffind_infragenus%3D%26find_isAPNIRecord%3Dtrue%26find_geoUnit%3D%26find_includePublicationAuthors%3Dtrue%26find_addedSince%3D%26find_family%3D%26find_genus%3DAtraphaxis%26find_sortByFamily%3Dtrue%26find_isGCIRecord%3Dtrue%26find_infrafamily%3D%26find_rankToReturn%3Dall%26find_publicationTitle%3D%26find_authorAbbrev%3D%26find_infraspecies%3D%26find_includeBasionymAuthors%3Dtrue%26find_modifiedSince%3D%26find_isIKRecord%3Dtrue%26find_species%3D%26output_format%3Dnormal) | Kyrgyzstan, Susamyr Ridge, Kara-Dzhigach. 7.07.2005, *Lazkov* (FRU) | 35 | GQ340057 | **KU724474** | **KU508768** |
| *A.* *tortuosa* [Losinsk.](http://www.ipni.org/ipni/idAuthorSearch.do?id=5767-1&back_page=%2Fipni%2FeditAdvPlantNameSearch.do%3Bjsessionid%3DCD5B69CD9903C9DD70928476C2EC3D1F%3Ffind_infragenus%3D%26find_isAPNIRecord%3Dtrue%26find_geoUnit%3D%26find_includePublicationAuthors%3Dtrue%26find_addedSince%3D%26find_family%3D%26find_genus%3DAtraphaxis%26find_sortByFamily%3Dtrue%26find_isGCIRecord%3Dtrue%26find_infrafamily%3D%26find_rankToReturn%3Dall%26find_publicationTitle%3D%26find_authorAbbrev%3D%26find_infraspecies%3D%26find_includeBasionymAuthors%3Dtrue%26find_modifiedSince%3D%26find_isIKRecord%3Dtrue%26find_species%3D%26output_format%3Dnormal) | Mongolia, South Goby aimag, SE of Nomgon, Shilt-Ula Mt. 19.07.1974. *Rachkovskaya, Volkova 6525* (LE) | 12  12-1 | **KJ707536**  **KJ707537** | **KU724475** | **KU508769** |
| *A.* *tortuosa* [Losinsk.](http://www.ipni.org/ipni/idAuthorSearch.do?id=5767-1&back_page=%2Fipni%2FeditAdvPlantNameSearch.do%3Bjsessionid%3DCD5B69CD9903C9DD70928476C2EC3D1F%3Ffind_infragenus%3D%26find_isAPNIRecord%3Dtrue%26find_geoUnit%3D%26find_includePublicationAuthors%3Dtrue%26find_addedSince%3D%26find_family%3D%26find_genus%3DAtraphaxis%26find_sortByFamily%3Dtrue%26find_isGCIRecord%3Dtrue%26find_infrafamily%3D%26find_rankToReturn%3Dall%26find_publicationTitle%3D%26find_authorAbbrev%3D%26find_infraspecies%3D%26find_includeBasionymAuthors%3Dtrue%26find_modifiedSince%3D%26find_isIKRecord%3Dtrue%26find_species%3D%26output_format%3Dnormal) | Mongolia, East Goby aimag, SW of Khuvsgul. 30.07.1971. *Isachenko, Rachkovskaya 1891*. (LE) | 52-10 | **KJ707542** | **KU724476** | **KU508770** |
| *A.* *tortuosa* [Losinsk.](http://www.ipni.org/ipni/idAuthorSearch.do?id=5767-1&back_page=%2Fipni%2FeditAdvPlantNameSearch.do%3Bjsessionid%3DCD5B69CD9903C9DD70928476C2EC3D1F%3Ffind_infragenus%3D%26find_isAPNIRecord%3Dtrue%26find_geoUnit%3D%26find_includePublicationAuthors%3Dtrue%26find_addedSince%3D%26find_family%3D%26find_genus%3DAtraphaxis%26find_sortByFamily%3Dtrue%26find_isGCIRecord%3Dtrue%26find_infrafamily%3D%26find_rankToReturn%3Dall%26find_publicationTitle%3D%26find_authorAbbrev%3D%26find_infraspecies%3D%26find_includeBasionymAuthors%3Dtrue%26find_modifiedSince%3D%26find_isIKRecord%3Dtrue%26find_species%3D%26output_format%3Dnormal) | Mongolia, South Goby aimag, SE of Khan-Bogd. 1972. *Rachkovskaya, Guricheva 21581*. (LE) | 99  99-1 | **KJ707546**  **KJ707547** | **KU724477** | **KU508771** |
| *A.* *tournefortii* [Jaub.](http://www.ipni.org/ipni/idAuthorSearch.do?id=4441-1&back_page=%2Fipni%2FeditAdvPlantNameSearch.do%3Bjsessionid%3DCD5B69CD9903C9DD70928476C2EC3D1F%3Ffind_infragenus%3D%26find_isAPNIRecord%3Dtrue%26find_geoUnit%3D%26find_includePublicationAuthors%3Dtrue%26find_addedSince%3D%26find_family%3D%26find_genus%3DAtraphaxis%26find_sortByFamily%3Dtrue%26find_isGCIRecord%3Dtrue%26find_infrafamily%3D%26find_rankToReturn%3Dall%26find_publicationTitle%3D%26find_authorAbbrev%3D%26find_infraspecies%3D%26find_includeBasionymAuthors%3Dtrue%26find_modifiedSince%3D%26find_isIKRecord%3Dtrue%26find_species%3D%26output_format%3Dnormal) & [Spach](http://www.ipni.org/ipni/idAuthorSearch.do?id=9939-1&back_page=%2Fipni%2FeditAdvPlantNameSearch.do%3Bjsessionid%3DCD5B69CD9903C9DD70928476C2EC3D1F%3Ffind_infragenus%3D%26find_isAPNIRecord%3Dtrue%26find_geoUnit%3D%26find_includePublicationAuthors%3Dtrue%26find_addedSince%3D%26find_family%3D%26find_genus%3DAtraphaxis%26find_sortByFamily%3Dtrue%26find_isGCIRecord%3Dtrue%26find_infrafamily%3D%26find_rankToReturn%3Dall%26find_publicationTitle%3D%26find_authorAbbrev%3D%26find_infraspecies%3D%26find_includeBasionymAuthors%3Dtrue%26find_modifiedSince%3D%26find_isIKRecord%3Dtrue%26find_species%3D%26output_format%3Dnormal) | Turkey, Yozgat. 13.06.1975. *Browicz, Zielinski* *582*. (LE) | 126 | **KU508748** | **KU724478** | **KU508772** |
| *A.* *tournefortii* [Jaub.](http://www.ipni.org/ipni/idAuthorSearch.do?id=4441-1&back_page=%2Fipni%2FeditAdvPlantNameSearch.do%3Bjsessionid%3DCD5B69CD9903C9DD70928476C2EC3D1F%3Ffind_infragenus%3D%26find_isAPNIRecord%3Dtrue%26find_geoUnit%3D%26find_includePublicationAuthors%3Dtrue%26find_addedSince%3D%26find_family%3D%26find_genus%3DAtraphaxis%26find_sortByFamily%3Dtrue%26find_isGCIRecord%3Dtrue%26find_infrafamily%3D%26find_rankToReturn%3Dall%26find_publicationTitle%3D%26find_authorAbbrev%3D%26find_infraspecies%3D%26find_includeBasionymAuthors%3Dtrue%26find_modifiedSince%3D%26find_isIKRecord%3Dtrue%26find_species%3D%26output_format%3Dnormal) & [Spach](http://www.ipni.org/ipni/idAuthorSearch.do?id=9939-1&back_page=%2Fipni%2FeditAdvPlantNameSearch.do%3Bjsessionid%3DCD5B69CD9903C9DD70928476C2EC3D1F%3Ffind_infragenus%3D%26find_isAPNIRecord%3Dtrue%26find_geoUnit%3D%26find_includePublicationAuthors%3Dtrue%26find_addedSince%3D%26find_family%3D%26find_genus%3DAtraphaxis%26find_sortByFamily%3Dtrue%26find_isGCIRecord%3Dtrue%26find_infrafamily%3D%26find_rankToReturn%3Dall%26find_publicationTitle%3D%26find_authorAbbrev%3D%26find_infraspecies%3D%26find_includeBasionymAuthors%3Dtrue%26find_modifiedSince%3D%26find_isIKRecord%3Dtrue%26find_species%3D%26output_format%3Dnormal) | Tavakkoli et al. 2015 |  | AB976654* | AB976706* | — |
| *Bactria lazkovii* O.V.Yurtseva & E.V.Mavrodiev | Kyrgyzstan, Naryn reg. Dzumgal d. Kavak-Too Ridge, Sary-Bulun. 7.07.2006. *Lazkov 24*. (MW) | 24 **(3**) | JQ288761 | **KU724451** | **KU508753** |
| *Bactria* *ovczinnikovii* (Czhukav.) O.V.Yurtseva & E.V.Mavrodiev (=*Polygonum ovczinnikovii Czukav.*) | Tadjikistan, Khablon reg. Shuroabad d. the Piandzh, Bakhorak × Bag. 25.07.2013. *Ukrainskaya et al. 12*. (MW) | 134 **(1)** | **KU508740** | **KU724452** | **KU508754** |
| *B. ovczinnikovii* | Tadjikistan, Schpilau, the Piandzh, Bag. 1.06.1960. *G.Nepli* (LE) | 2011-9**(2)** | **KU508741** | — | **KU508755** |
| *Duma florulenta* (Meisn.) T.M. Schuster | Schuster et al., 2011a |  | JF831205* | — | JF831298* |
| *D. coccoloboides* (J.M.Black) T.M.Schuster | Schuster et al., 2011a |  | JF831204* | — | JF831297* |
| *D. horrida* (H.Gross.) T.M.Schuster | Schuster et al., 2011a |  | JF831206* | — | JF831299* |
| *Fallopia convolvulus* (L.) Á Löve | Yurtseva et al. 2010; Tavakkoli et al. 2015;  Yu et al. (unpublished) |  | JQ288753 | AB976708* | EU024782* |
| *F. dumetorum* (L.) Holub | Tavakkoli et al., 2015; Yu et al. (unpublished) |  | AB976657* | AB976709* | EU024785* |
| *F. baldschuanica* (Regel) Holub | Tavakkoli et al. 2015 |  | AB976655* | AB976707* | — |
| *Knorringia sibirica* (Laxm.) Tzvelev | Sanchez et al. 2009;  Wei, Ze (unpublished) |  | GQ206253* | — | EU109596* |
| *Muehlenbeckia astonii* Petrie | Schuster et al. 2011a |  | — | — | JF831302* |
| *M. australis* Meisn. | Schuster et al., 2011b |  | JF831208* | — | — |
| *M. platyclada* (F.Muell.) Meisn. | Won, Park (unpublished); Fan et al (2013);  Schuster et al., 2011a |  | AF189738* | JN235019* | JF831311* |
| *M. rhyticarya* F. Muell. | Schuster et al. 2011a |  | — | — | JF831312* |
| *Polygonella americana* Small | USA, Arcansas, Polk Co., Ouchita Nat. Forest. 19.09.1966. *Memaree 54535*. [NDA] | 74 | **KU508738** | — | — |
| *P. articulata* Meisn. | Kim, Donohue, 2008 |  | EF653683* | — | — |
| *P. basiramia* (Small.) G.L.Nesom & V.M.Bates | Schuster et al., 2011b |  | — | — | JN161141* |
| *Polygonella ciliata* Meisn. | Schuster et al., 2011b |  | — | — | JN161142* |
| *P. gracilis* Meisn. | USA, South California, Jasper Co., N of Savannah River. 17.09.1967. *Radford et al. 11506* [NDA] | 76 | **KU508736** | — | — |
| *P. macrophylla* Small | Schuster et al., 2011b |  | — | — | JN161143* |
| *P. polygama* Engelm. & A.Gray | USA, Florida, Marion Co., N. of Silver Glen Springs, 24.10.2002. *Slaughter, Meeks 13590.* [NDA] | 77 | **KU508737** | — | — |
| *Polygonum* *acetosum* [M.Bieb.](http://www.ipni.org/ipni/idAuthorSearch.do?id=6129-1&back_page=%2Fipni%2FeditAdvPlantNameSearch.do%3Ffind_infragenus%3D%26find_isAPNIRecord%3Dtrue%26find_geoUnit%3D%26find_includePublicationAuthors%3Dtrue%26find_addedSince%3D%26find_family%3D%26find_genus%3DPolygonum%26find_sortByFamily%3Dtrue%26find_isGCIRecord%3Dtrue%26find_infrafamily%3D%26find_rankToReturn%3Dall%26find_publicationTitle%3D%26find_authorAbbrev%3D%26find_infraspecies%3D%26find_includeBasionymAuthors%3Dtrue%26find_modifiedSince%3D%26find_isIKRecord%3Dtrue%26find_species%3D%26output_format%3Dnormal) | Yurtseva et al. 2010 |  | GQ339948 | — | — |
| *P.* *alpestre* [C.A.Mey.](http://www.ipni.org/ipni/idAuthorSearch.do?id=6411-1&back_page=%2Fipni%2FeditAdvPlantNameSearch.do%3Ffind_infragenus%3D%26find_isAPNIRecord%3Dtrue%26find_geoUnit%3D%26find_includePublicationAuthors%3Dtrue%26find_addedSince%3D%26find_family%3D%26find_genus%3DPolygonum%26find_sortByFamily%3Dtrue%26find_isGCIRecord%3Dtrue%26find_infrafamily%3D%26find_rankToReturn%3Dall%26find_publicationTitle%3D%26find_authorAbbrev%3D%26find_infraspecies%3D%26find_includeBasionymAuthors%3Dtrue%26find_modifiedSince%3D%26find_isIKRecord%3Dtrue%26find_species%3D%26output_format%3Dnormal) | Yurtseva et al. 2010; Tavakkoli et al. 2015 |  | GQ339958 | AB976711* | — |
| *P. arenarium* [Waldst.](http://www.ipni.org/ipni/idAuthorSearch.do?id=12989-1&back_page=%2Fipni%2FeditAdvPlantNameSearch.do%3Ffind_infragenus%3D%26find_isAPNIRecord%3Dtrue%26find_geoUnit%3D%26find_includePublicationAuthors%3Dtrue%26find_addedSince%3D%26find_family%3D%26find_genus%3DPolygonum%26find_sortByFamily%3Dtrue%26find_isGCIRecord%3Dtrue%26find_infrafamily%3D%26find_rankToReturn%3Dall%26find_publicationTitle%3D%26find_authorAbbrev%3D%26find_infraspecies%3D%26find_includeBasionymAuthors%3Dtrue%26find_modifiedSince%3D%26find_isIKRecord%3Dtrue%26find_species%3D%26output_format%3Dnormal) & [Kit.](http://www.ipni.org/ipni/idAuthorSearch.do?id=4819-1&back_page=%2Fipni%2FeditAdvPlantNameSearch.do%3Ffind_infragenus%3D%26find_isAPNIRecord%3Dtrue%26find_geoUnit%3D%26find_includePublicationAuthors%3Dtrue%26find_addedSince%3D%26find_family%3D%26find_genus%3DPolygonum%26find_sortByFamily%3Dtrue%26find_isGCIRecord%3Dtrue%26find_infrafamily%3D%26find_rankToReturn%3Dall%26find_publicationTitle%3D%26find_authorAbbrev%3D%26find_infraspecies%3D%26find_includeBasionymAuthors%3Dtrue%26find_modifiedSince%3D%26find_isIKRecord%3Dtrue%26find_species%3D%26output_format%3Dnormal) | Yurtseva et al. 2010 | 1 | GQ339924 | — | **KU508749** |
| *P.* *aridum* [Boiss.](http://www.ipni.org/ipni/idAuthorSearch.do?id=16284-1&back_page=%2Fipni%2FeditAdvPlantNameSearch.do%3Bjsessionid%3DCD5B69CD9903C9DD70928476C2EC3D1F%3Ffind_infragenus%3D%26find_isAPNIRecord%3Dtrue%26find_geoUnit%3D%26find_includePublicationAuthors%3Dtrue%26find_addedSince%3D%26find_family%3D%26find_genus%3DAtraphaxis%26find_sortByFamily%3Dtrue%26find_isGCIRecord%3Dtrue%26find_infrafamily%3D%26find_rankToReturn%3Dall%26find_publicationTitle%3D%26find_authorAbbrev%3D%26find_infraspecies%3D%26find_includeBasionymAuthors%3Dtrue%26find_modifiedSince%3D%26find_isIKRecord%3Dtrue%26find_species%3D%26output_format%3Dnormal) & [Hausskn.](http://www.ipni.org/ipni/idAuthorSearch.do?id=3732-1&back_page=%2Fipni%2FeditAdvPlantNameSearch.do%3Bjsessionid%3DCD5B69CD9903C9DD70928476C2EC3D1F%3Ffind_infragenus%3D%26find_isAPNIRecord%3Dtrue%26find_geoUnit%3D%26find_includePublicationAuthors%3Dtrue%26find_addedSince%3D%26find_family%3D%26find_genus%3DAtraphaxis%26find_sortByFamily%3Dtrue%26find_isGCIRecord%3Dtrue%26find_infrafamily%3D%26find_rankToReturn%3Dall%26find_publicationTitle%3D%26find_authorAbbrev%3D%26find_infraspecies%3D%26find_includeBasionymAuthors%3Dtrue%26find_modifiedSince%3D%26find_isIKRecord%3Dtrue%26find_species%3D%26output_format%3Dnormal) ex [Boiss.](http://www.ipni.org/ipni/idAuthorSearch.do?id=16284-1&back_page=%2Fipni%2FeditAdvPlantNameSearch.do%3Bjsessionid%3DCD5B69CD9903C9DD70928476C2EC3D1F%3Ffind_infragenus%3D%26find_isAPNIRecord%3Dtrue%26find_geoUnit%3D%26find_includePublicationAuthors%3Dtrue%26find_addedSince%3D%26find_family%3D%26find_genus%3DAtraphaxis%26find_sortByFamily%3Dtrue%26find_isGCIRecord%3Dtrue%26find_infrafamily%3D%26find_rankToReturn%3Dall%26find_publicationTitle%3D%26find_authorAbbrev%3D%26find_infraspecies%3D%26find_includeBasionymAuthors%3Dtrue%26find_modifiedSince%3D%26find_isIKRecord%3Dtrue%26find_species%3D%26output_format%3Dnormal) | Tavakkoli et al. 2015 |  | AB976641* | AB976693* | — |
| *P.* *aviculare* L. | Yurtseva et al. 2010;  Fan et al. 2013;  Kim, Donohue, 2008 |  | GQ339928 | JN235018* | EF653787* |
| *P.* *boreale* [Small](http://www.ipni.org/ipni/idAuthorSearch.do?id=9754-1&back_page=%2Fipni%2FeditAdvPlantNameSearch.do%3Ffind_infragenus%3D%26find_isAPNIRecord%3Dtrue%26find_geoUnit%3D%26find_includePublicationAuthors%3Dtrue%26find_addedSince%3D%26find_family%3D%26find_genus%3DPolygonum%26find_sortByFamily%3Dtrue%26find_isGCIRecord%3Dtrue%26find_infrafamily%3D%26find_rankToReturn%3Dall%26find_publicationTitle%3D%26find_authorAbbrev%3D%26find_infraspecies%3D%26find_includeBasionymAuthors%3Dtrue%26find_modifiedSince%3D%26find_isIKRecord%3Dtrue%26find_species%3D%26output_format%3Dnormal) | Russia, Murmansk Reg., Kandalaksha distr., Kandalakshsky reservation, Porya Guba. 10.07.2011. *Kozhin* (MW) | 11 | **KU508739** | — | **KU508750** |
| *P. bornmuelleri* Litv. | Yurtseva et al. 2010 |  | GQ339987 | — | — |
| *P.* *botuliforme* [Mozaff.](http://www.ipni.org/ipni/idAuthorSearch.do?id=23696-1&back_page=%2Fipni%2FeditAdvPlantNameSearch.do%3Bjsessionid%3DCD5B69CD9903C9DD70928476C2EC3D1F%3Ffind_infragenus%3D%26find_isAPNIRecord%3Dtrue%26find_geoUnit%3D%26find_includePublicationAuthors%3Dtrue%26find_addedSince%3D%26find_family%3D%26find_genus%3DAtraphaxis%26find_sortByFamily%3Dtrue%26find_isGCIRecord%3Dtrue%26find_infrafamily%3D%26find_rankToReturn%3Dall%26find_publicationTitle%3D%26find_authorAbbrev%3D%26find_infraspecies%3D%26find_includeBasionymAuthors%3Dtrue%26find_modifiedSince%3D%26find_isIKRecord%3Dtrue%26find_species%3D%26output_format%3Dnormal) | Tavakkoli et al. 2015 |  | AB976644* | AB976696* | — |
| *P. cognatum* Meisn. | Yurtseva et al. 2010;  Xu et al. (unpublished) |  | GQ339994 | — | FJ627272* |
| *P.* *confertiflorum* [Nutt.](http://www.ipni.org/ipni/idAuthorSearch.do?id=24198-1&back_page=%2Fipni%2FeditAdvPlantNameSearch.do%3Ffind_infragenus%3D%26find_isAPNIRecord%3Dtrue%26find_geoUnit%3D%26find_includePublicationAuthors%3Dtrue%26find_addedSince%3D%26find_family%3D%26find_genus%3DPolygonum%26find_sortByFamily%3Dtrue%26find_isGCIRecord%3Dtrue%26find_infrafamily%3D%26find_rankToReturn%3Dall%26find_publicationTitle%3D%26find_authorAbbrev%3D%26find_infraspecies%3D%26find_includeBasionymAuthors%3Dtrue%26find_modifiedSince%3D%26find_isIKRecord%3Dtrue%26find_species%3D%26output_format%3Dnormal) ex [Piper](http://www.ipni.org/ipni/idAuthorSearch.do?id=24883-1&back_page=%2Fipni%2FeditAdvPlantNameSearch.do%3Ffind_infragenus%3D%26find_isAPNIRecord%3Dtrue%26find_geoUnit%3D%26find_includePublicationAuthors%3Dtrue%26find_addedSince%3D%26find_family%3D%26find_genus%3DPolygonum%26find_sortByFamily%3Dtrue%26find_isGCIRecord%3Dtrue%26find_infrafamily%3D%26find_rankToReturn%3Dall%26find_publicationTitle%3D%26find_authorAbbrev%3D%26find_infraspecies%3D%26find_includeBasionymAuthors%3Dtrue%26find_modifiedSince%3D%26find_isIKRecord%3Dtrue%26find_species%3D%26output_format%3Dnormal) | Yurtseva et al. 2010 |  | GQ339996 | — | — |
| *P.* *douglasii* [Greene](http://www.ipni.org/ipni/idAuthorSearch.do?id=3366-1&back_page=%2Fipni%2FeditAdvPlantNameSearch.do%3Ffind_infragenus%3D%26find_isAPNIRecord%3Dtrue%26find_geoUnit%3D%26find_includePublicationAuthors%3Dtrue%26find_addedSince%3D%26find_family%3D%26find_genus%3DPolygonum%26find_sortByFamily%3Dtrue%26find_isGCIRecord%3Dtrue%26find_infrafamily%3D%26find_rankToReturn%3Dall%26find_publicationTitle%3D%26find_authorAbbrev%3D%26find_infraspecies%3D%26find_includeBasionymAuthors%3Dtrue%26find_modifiedSince%3D%26find_isIKRecord%3Dtrue%26find_species%3D%26output_format%3Dnormal) | Yurtseva et al. 2010 |  | GQ339998 | — | — |
| *P.* *dumosum* [Boiss.](http://www.ipni.org/ipni/idAuthorSearch.do?id=16284-1&back_page=%2Fipni%2FeditAdvPlantNameSearch.do%3Bjsessionid%3DCD5B69CD9903C9DD70928476C2EC3D1F%3Ffind_infragenus%3D%26find_isAPNIRecord%3Dtrue%26find_geoUnit%3D%26find_includePublicationAuthors%3Dtrue%26find_addedSince%3D%26find_family%3D%26find_genus%3DAtraphaxis%26find_sortByFamily%3Dtrue%26find_isGCIRecord%3Dtrue%26find_infrafamily%3D%26find_rankToReturn%3Dall%26find_publicationTitle%3D%26find_authorAbbrev%3D%26find_infraspecies%3D%26find_includeBasionymAuthors%3Dtrue%26find_modifiedSince%3D%26find_isIKRecord%3Dtrue%26find_species%3D%26output_format%3Dnormal) | Tavakkoli et al. 2015 |  | AB976645* | AB976697* | — |
| *P. luzuloides* [Jaub.](http://www.ipni.org/ipni/idAuthorSearch.do?id=4441-1&back_page=%2Fipni%2FeditAdvPlantNameSearch.do%3Ffind_infragenus%3D%26find_isAPNIRecord%3Dtrue%26find_geoUnit%3D%26find_includePublicationAuthors%3Dtrue%26find_addedSince%3D%26find_family%3D%26find_genus%3DPolygonum%26find_sortByFamily%3Dtrue%26find_isGCIRecord%3Dtrue%26find_infrafamily%3D%26find_rankToReturn%3Dall%26find_publicationTitle%3D%26find_authorAbbrev%3D%26find_infraspecies%3D%26find_includeBasionymAuthors%3Dtrue%26find_modifiedSince%3D%26find_isIKRecord%3Dtrue%26find_species%3D%26output_format%3Dnormal) & [Spach](http://www.ipni.org/ipni/idAuthorSearch.do?id=9939-1&back_page=%2Fipni%2FeditAdvPlantNameSearch.do%3Ffind_infragenus%3D%26find_isAPNIRecord%3Dtrue%26find_geoUnit%3D%26find_includePublicationAuthors%3Dtrue%26find_addedSince%3D%26find_family%3D%26find_genus%3DPolygonum%26find_sortByFamily%3Dtrue%26find_isGCIRecord%3Dtrue%26find_infrafamily%3D%26find_rankToReturn%3Dall%26find_publicationTitle%3D%26find_authorAbbrev%3D%26find_infraspecies%3D%26find_includeBasionymAuthors%3Dtrue%26find_modifiedSince%3D%26find_isIKRecord%3Dtrue%26find_species%3D%26output_format%3Dnormal) | Yurtseva et al. 2010; Tavakkoli et al. 2015 |  | GQ339937 | AB976713* | — |
| *P.* *khajeh-jamali* [Khosravi](http://www.ipni.org/ipni/idAuthorSearch.do?id=20001696-1&back_page=%2Fipni%2FeditAdvPlantNameSearch.do%3Bjsessionid%3DCD5B69CD9903C9DD70928476C2EC3D1F%3Ffind_infragenus%3D%26find_isAPNIRecord%3Dtrue%26find_geoUnit%3D%26find_includePublicationAuthors%3Dtrue%26find_addedSince%3D%26find_family%3D%26find_genus%3DAtraphaxis%26find_sortByFamily%3Dtrue%26find_isGCIRecord%3Dtrue%26find_infrafamily%3D%26find_rankToReturn%3Dall%26find_publicationTitle%3D%26find_authorAbbrev%3D%26find_infraspecies%3D%26find_includeBasionymAuthors%3Dtrue%26find_modifiedSince%3D%26find_isIKRecord%3Dtrue%26find_species%3D%26output_format%3Dnormal) & [Poormahdi](http://www.ipni.org/ipni/idAuthorSearch.do?id=20014587-1&back_page=%2Fipni%2FeditAdvPlantNameSearch.do%3Bjsessionid%3DCD5B69CD9903C9DD70928476C2EC3D1F%3Ffind_infragenus%3D%26find_isAPNIRecord%3Dtrue%26find_geoUnit%3D%26find_includePublicationAuthors%3Dtrue%26find_addedSince%3D%26find_family%3D%26find_genus%3DAtraphaxis%26find_sortByFamily%3Dtrue%26find_isGCIRecord%3Dtrue%26find_infrafamily%3D%26find_rankToReturn%3Dall%26find_publicationTitle%3D%26find_authorAbbrev%3D%26find_infraspecies%3D%26find_includeBasionymAuthors%3Dtrue%26find_modifiedSince%3D%26find_isIKRecord%3Dtrue%26find_species%3D%26output_format%3Dnormal) | Tavakkoli et al. 2015 |  | AB976648* | AB976700* | — |
| *Polygonum kelloggii* Greene | Yurtseva et al. 2010 |  | GQ340035 | — | — |
| *P. maritimum* L. | Yurtseva et al. 2010 |  | GQ340014 | — | — |
| *P.* *molliiforme* [Boiss.](http://www.ipni.org/ipni/idAuthorSearch.do?id=16284-1&back_page=%2Fipni%2FeditAdvPlantNameSearch.do%3Ffind_infragenus%3D%26find_isAPNIRecord%3Dtrue%26find_geoUnit%3D%26find_includePublicationAuthors%3Dtrue%26find_addedSince%3D%26find_family%3D%26find_genus%3DPolygonum%26find_sortByFamily%3Dtrue%26find_isGCIRecord%3Dtrue%26find_infrafamily%3D%26find_rankToReturn%3Dall%26find_publicationTitle%3D%26find_authorAbbrev%3D%26find_infraspecies%3D%26find_includeBasionymAuthors%3Dtrue%26find_modifiedSince%3D%26find_isIKRecord%3Dtrue%26find_species%3D%26output_format%3Dnormal) | Yurtseva et al. 2012b | 8 | JQ288763 | — | **KU508751** |
| *P.* *paronychioides* [C.A.Mey.](http://www.ipni.org/ipni/idAuthorSearch.do?id=6411-1&back_page=%2Fipni%2FeditAdvPlantNameSearch.do%3Ffind_infragenus%3D%26find_isAPNIRecord%3Dtrue%26find_geoUnit%3D%26find_includePublicationAuthors%3Dtrue%26find_addedSince%3D%26find_family%3D%26find_genus%3DPolygonum%26find_sortByFamily%3Dtrue%26find_isGCIRecord%3Dtrue%26find_infrafamily%3D%26find_rankToReturn%3Dall%26find_publicationTitle%3D%26find_authorAbbrev%3D%26find_infraspecies%3D%26find_includeBasionymAuthors%3Dtrue%26find_modifiedSince%3D%26find_isIKRecord%3Dtrue%26find_species%3D%26output_format%3Dnormal) | Yurtseva et al. 2012b; Tavakkoli et al. 2015; | 21 | JQ288768 | AB976714* | **KU508752** |
| *P.* *plebeium* R.Br. | Yurtseva et al. 2012b; Wei, Ze (unpublished) |  | JQ288762 | — | EU109598 |
| *P.* *thymifolium* [Jaub.](http://www.ipni.org/ipni/idAuthorSearch.do?id=4441-1&back_page=%2Fipni%2FeditAdvPlantNameSearch.do%3Ffind_infragenus%3D%26find_isAPNIRecord%3Dtrue%26find_geoUnit%3D%26find_includePublicationAuthors%3Dtrue%26find_addedSince%3D%26find_family%3D%26find_genus%3DPolygonum%26find_sortByFamily%3Dtrue%26find_isGCIRecord%3Dtrue%26find_infrafamily%3D%26find_rankToReturn%3Dall%26find_publicationTitle%3D%26find_authorAbbrev%3D%26find_infraspecies%3D%26find_includeBasionymAuthors%3Dtrue%26find_modifiedSince%3D%26find_isIKRecord%3Dtrue%26find_species%3D%26output_format%3Dnormal) & [Spach](http://www.ipni.org/ipni/idAuthorSearch.do?id=9939-1&back_page=%2Fipni%2FeditAdvPlantNameSearch.do%3Ffind_infragenus%3D%26find_isAPNIRecord%3Dtrue%26find_geoUnit%3D%26find_includePublicationAuthors%3Dtrue%26find_addedSince%3D%26find_family%3D%26find_genus%3DPolygonum%26find_sortByFamily%3Dtrue%26find_isGCIRecord%3Dtrue%26find_infrafamily%3D%26find_rankToReturn%3Dall%26find_publicationTitle%3D%26find_authorAbbrev%3D%26find_infraspecies%3D%26find_includeBasionymAuthors%3Dtrue%26find_modifiedSince%3D%26find_isIKRecord%3Dtrue%26find_species%3D%26output_format%3Dnormal) | Yurtseva et al. 2010; Tavakkoli et al. 2015 |  | GQ340055 | AB976715* | — |
| *P.* *salicornioides* [Jaub.](http://www.ipni.org/ipni/idAuthorSearch.do?id=4441-1&back_page=%2Fipni%2FeditAdvPlantNameSearch.do%3Bjsessionid%3DCD5B69CD9903C9DD70928476C2EC3D1F%3Ffind_infragenus%3D%26find_isAPNIRecord%3Dtrue%26find_geoUnit%3D%26find_includePublicationAuthors%3Dtrue%26find_addedSince%3D%26find_family%3D%26find_genus%3DAtraphaxis%26find_sortByFamily%3Dtrue%26find_isGCIRecord%3Dtrue%26find_infrafamily%3D%26find_rankToReturn%3Dall%26find_publicationTitle%3D%26find_authorAbbrev%3D%26find_infraspecies%3D%26find_includeBasionymAuthors%3Dtrue%26find_modifiedSince%3D%26find_isIKRecord%3Dtrue%26find_species%3D%26output_format%3Dnormal) & [Spach](http://www.ipni.org/ipni/idAuthorSearch.do?id=9939-1&back_page=%2Fipni%2FeditAdvPlantNameSearch.do%3Bjsessionid%3DCD5B69CD9903C9DD70928476C2EC3D1F%3Ffind_infragenus%3D%26find_isAPNIRecord%3Dtrue%26find_geoUnit%3D%26find_includePublicationAuthors%3Dtrue%26find_addedSince%3D%26find_family%3D%26find_genus%3DAtraphaxis%26find_sortByFamily%3Dtrue%26find_isGCIRecord%3Dtrue%26find_infrafamily%3D%26find_rankToReturn%3Dall%26find_publicationTitle%3D%26find_authorAbbrev%3D%26find_infraspecies%3D%26find_includeBasionymAuthors%3Dtrue%26find_modifiedSince%3D%26find_isIKRecord%3Dtrue%26find_species%3D%26output_format%3Dnormal) | Tavakkoli et al. 2015 |  | AB976650* | AB976702* | — |
| *P.* *spinosum* H.Gross | Tavakkoli et al. 2015 |  | AB976647* | AB976699* | — |
| *Reynoutria japonica* Houtt. | Won, Park (unpublished);  Fan et al. 2013;  Yu et al. (unpublished) |  | AF040071* | JN235020* | EU024786* |
| *R. sachalinensis* Nakai | Won, Park, 1997 ;  Schuster et al., 2011a |  | AF040074* | — | JF831320* |

MW — Lomonosov Moscow State University, Russia, Moscow.

LE — V.L. Komarov Botanical Institute of the Russian Academy of Sciences (RAS), Saint Petersburg, Russia.

MHA — Main Botanical Garden RAS, Moscow, Russia.

FRU — Main Botanical Garden, National Academy of Science, Bishkek, Kyrgyzstan.

NDA — North Dakota State University, Fargo, North Dakota, U.S.A.

* — sequences from Genbank.

Numbers in bold were obtained for this study

References

Fan DM, Chen JH, Meng Y, Wen J, Huang JL, Yang YP. Molecular phylogeny of *Koenigia* L. (Polygonaceae: Persicarieae): implications for classification, character evolution and biogeography. Molecular Phylogenetics and Evolution 69 (3), 1093-1100 (2013).

Kim S-T, Donoghue MJ. Molecular phylogeny of Persicaria (Persicarieae, Polygonaceae) Systematic Botany 33 (1), 77-86 (2008).

Schuster TM, Wilson KL, Kron KA (2011a) Phylogenetic relationships of *Muehlenbeckia*, *Fallopia*, and *Reynoutria* (Polygonaceae) investigated with chloroplast and nuclear sequence data. International Journal of Plant Sciences 172(8): 1053–1066.

Schuster TM, Reveal JL, Kron KA (2011b) Phylogeny of *Polygoneae* (Polygonaceae: polygonoideae). Taxon 60(6): 1653–1666.

Wei S., Ze Z-Z. Molecular phylogeny of tribes Persicarieae and Polygoneae (Polygonaceae) based on chloroplast sequence (unpublished).

Won H, Park C.-W. Direct Submission Submitted (23-DEC-1997) Department of Biology, Seoul National University, Shillim-dong san 56-1, Kwanak-ku, Seoul 151-742, Korea.

Won H, Park C-W. Molecular Phylogeny of the Genus *Fallopia* (Polygonaceae) (unpublished).

Xu CM, Yu WG, Li FZ. Phylogenetic origin of Polygonum perfoliatum inferred from plastid trnL-F sequences (unpublished).

Yu W, Li F, Xu C, Zhu L. Systematic position of *Reynoutria* Houtt. and *Polygonum sibiricum* Laxm. based on chloroplast trnL-F and matK sequences (unpublished).

Yu W, Li F, Xu C. The phylogeny of Polygoneae inferred from chloroplast DNA trnL-F sequences (unpublished).

Yu W, Li F, Xu C, Zhu L. Systematic position of Reynoutria Houtt. and Polygonum sibiricum Laxm. based on chloroplast trnL-F and matK (unpublished).
