# Supplementary material for: What is Atraphaxis L. (Polygonaceae, Polygoneae): cryptic taxa and resolved taxonomic complexity instead of the formal lumping and the lack of morphological synapomorphies
Source: PeerJ. 2016 May 3;4:e1977. doi: 10.7717/peerj.1977 (PMC4860328; doi:10.7717/peerj.1977)
Supplement: Supplemental Information 4 [file peerj-04-1977-s004.doc]

**Table S4.** Characteristics of the perianth and the achene in *Bactria*, *Polygonum* sect. *Spinescentia,* and *Atraphaxis* s.str.

| Species | N of segments | Length of tube,  mm | Filiform part of tube, mm | Extended  part of tube,  mm | Outer segments, mm | Inner segments, mm | Shape of tube exten-sion | Shape of segments | Outer/innersegments length ratio | Perianth partition | Achene size, mm |
| --- | --- | --- | --- | --- | --- | --- | --- | --- | --- | --- | --- |
| *Bactria lazkovii*  *B. ovczinnikovii* | 5  5-6 | 0.5-0.6  0.5-0.8 | 0  0.10-0.15 | 0.5-0.6  0.4-0.7 | 2.0-3.3 × 1.5-1.7  3.0-4.0 × 1.2-1.7 | 2.0-2.5 × 1.2-1.5  3.0-4.0 × 1.4-1.6 | cup  funnel | oblong-elliptical  lanceolate | equal  equal | 4/5-5/6  5/6-8/10 | 2.5-3.0 × 1.8-2.0  4.0-5.0 × 2.2-2.8 |
| *P. salicornioides* | 5 | 1.6-2.2 | 0.3 | 1.3-1.9 | 3.9-4.1 × 2.1-2.3 | 3.6-3.8 × 1.8-1.9 | funnel | oblong-ovate | equal | 2/3 | 3.5-4.0 × 2.8-3.4 |
| *Atraphaxis ariana* | 5 | 2.0-2.5 | 0.4-0.5 | 1.6-2.0 | 3.0-5.0 × 3.0-4.0 | 3.0-5.0 × 3.0-4.0 | funnel | broadly-ovate | equal | 3/4 | 2.5-3.0 × 1.5-1.8 |
| *A. toktogulica* | 5 | 1.2-1.5 | 0.7-1.0 | 0.7-1.0 | 2.5-3.5 × 1.2-1.4 | 2.5-3.5 × 1.0-1.3 | funnel | oblong-elliptical | equal | 3/4 | 2.5-3.0 × 1.5-2.0 |
| *A. atraphaxiformis*  *A. tortuosa* | 5  5 | 1.0-1.5  1.0-1.7 | 0.5-0.7  0.5-0.7 | 0.5-1.0  0.5-1.0 | 2.2-3.2 × 1.2-1.3  2.5-3.7 × 1.2-1.5 | 2.2-3.2 × 1.2-1.3  2.5-3.8 × 1.3-1.6 | funnel  funnel | oblong-elliptical oblong-elliptical | equal  equal | 2/3-3/4  2/3-3/4 | 3.0-3.7 × 1.5-2.1  3.5-4.5 × 2.0-3.0 |
| *A. badghysi*  *A. aucherii* | 5  5 | 2.7-4.0  1.4-2.0 | 1.7-3.0  1.0-1.5 | 1.0  0.4-0.5 | 3.5-4.5 × 2.0-2.5  2.0-2.5 × 1.3-1.7 | 3.5-4.5 × 3.0-4.0  2.0-3.2 × 1.3-1.9 | funnel  wedge | rotundate  broadly-elliptical | equal  equal | ½  2/3 | 4.5-5.0 × 3.0-3.5  4.0-4.5 × 2.0-2.5 |
| *A. grandiflora*  *A. angustifolia*  *A. manshurica* | 5  5  5 | 1.5-2.1  1.5-2.5  1.0-1.6 | 0.8-1.4  1.0-1,9  0.5-1.0 | 0.7-1.0  0.5-0.6  0.5-0.6 | 4.6-5.0 × 2.4-2.5  2.8-3.7 × 2.2-2.5  1.5-1.8 × 1.1-1.2 | 5.5-6.0 × 4.3-5.5  3.5-4.5 × 3.2-3.7  1.8-2.0× 1.1-1.2 | cup  cup  cup | broadly-elliptical  broadly-elliptical  broadly elliptical | subequal  subequal  subequal | ¾  2/3  2/3 | 3.5-4.0 × 3.0-3.1  3.0-3.5 × 2.0-2.2  4.0-5.0 × 1.5-2.5 |
| *A. bracteata* | 5 | 1.5-2.1 | 1.0-1.5 | 0.5-1.0 | 2.0-3.0 × 2.0-3.0 | 5.5-6.5 × 7.0-8.0 | wedge | rotundate | ½ | 2/3 | 4.0-5.0 × 1.5-2.5 |
| *A. avenia*  *A. seravschanica*  *A. kopetdagensis*  *A. muschketowi*  *A. laetevirens*  *A. pyrifolia* | 5  5  5  5  5  5 | 5.0-7.5  5.0-6.5  5.2-5.5  4.0-6.5  4.5-6.0  4.5-7.5 | 4.0-6.5  3.5-5.5  3.0-3.5  3.0-6.0  4.0-5.5  5.0-7.0 | 1.0  1.0-1.5  1.9-2.0  0.5-1.0  0.5-0.7  0.5-1.0 | 2.5-3.0 × 1.5-2.0  3.0-3.5 × 2.0-3.0  2.5-3.3 × 2.0-2.7  2.3-4.0 × 1.2-2.0  2.5-4.0 × 2.0-2.5  3.0-4.0 × 2.0-2.5 | 5.0-6.0 × 4.0-4.5  6.0-8.0 × 6.0-8.0  6.0-7.0 × 7.0-9.0  6.5-7.5 × 7.0-9.0  6.0-8.0 × 6.0-9.0  6.0-7.0 × 6.5-7.0 | wedge  cup  cup  wedge  wedge  wedge | broadly elliptical  broadly elliptical  cordiate  cordiate-rotundate  cordiate-rotundate  cordiate-rotundate | ½  ½  ½  ½  ½  ½ | ½  ½  ½  ½  1/2  1/2 | 3.0-3.2 × 2.3-2.8  3.0-4.0 × 2.0-3.0  3.5-4.0 × 2.3-2.5  3.0-3.5 × 1.8-2.0  3.0-3.5 × 2.3-2.5  3.0-3.2 × 1.8-2.0 |
| *A. caucasica* | 4-5 | 5.0-5.5 | 4.5-5.0 | 0.5 | 4.0-4.5 × 3.0-4.0 | 6.0-7.0 × 7.5-9.0 | wedge | rotundate | 1/2 | 1/2 | 3.0-3.3 × 1.6-2.0 |
| *A.teretifolia* | 5 | 0.6-0.8 | 0.5-0.7 | 0.1 | 1.0-1.5 × 1.0-1.5 | 4.0-5.0 × 4.0-6.0 | wedge | rotundate | 1/3-1/4 | 9/10 | 2.5-2.6 × 1.4-1.6 |
| *A. pungens* | 5 | 2.7-3.5 | 2.8-3.0 | 0.5 | 2.0-2.5 × 2.5-4.0 | 4.5-6.0 × 4.5-7.0 | Wedge | rotundate | 1/2 | 2/3 | 2.6-3.3 × 1.3-2.0 |
| *A. frutescens*  *A. decipiens*  *A. virgata*  *A. rodinii* | 5  5  5  5 | 2.5-3.0  2.0-3.0  2.5-3.0  2.5-3.5 | 2.0-2.5  1.3-2.0  1.5-2.5  2.0-2.5 | 0.5-1.0  0.7-1.0  0.8-1.0  0.5-1.0 | 1.8-2.0 × 1.0-1.5  1.3-2.5 × 1.0-1.6  2.0-2.5 × 1.2-1.5  1.8-2.0 × 1.2-1.5 | 4.0-6.0 × 4.0-6.0  3.7-4.6 × 3.5-5.0  4.5-5.0 × 3.5-4.0  4.5-6.0 × 4.5-5.0 | wedge  cup  wedge  cup | broadly elliptical  broadly elliptical  broadly elliptical  broadly elliptical | **½**  ½  ½  ½ | 2/3  2/3  2/3  2/3 | 3.4-3.5 × 1.6-1.7  3.0-3.5 × 1.3-1.5  3.0-3.7 × 1.4-1.7  4.0-5.0 × 2,0-2.5 |
| *A. spinosa*  *A. fischeri*  *A. canescens*  *A. compacta*  *A. karataviensis*  *A. replicata* | 4  4  4  4  4  4 | 2.0-3.0  2.5-3.8  1.7-3.0  2.5-2.6  2.0-3.0  3.0-4.0 | 2.0-2.5  2.2-3.3  1.5-2.0  1.5-2.0  1.5-2.5  3.0-3.1 | 0.3-0.5  0.3-0.5  0.3-0.5  0.5-1.0  0.5-0.6  0.5-1.0 | 1.7-2.0 × 1.0-1.5  1.7-2.0 × 1.0-1.5  2.0-3.5 × 1.5-2.0  3.4-4.5 × 2.7-4.0  2.0-2.5× 1.0-1.5  2.5-3.0 × 1.5-2.0 | 4.5-6.0 × 4.5-6.0  4.5-5.5 × 4.0-7.0  4.0-5.0 × 4.0-6.0  7.0-8.0 × 8.0-10  5.0-6.0 × 5.0-6.0  6.0-8.0 × 8.0-9.0 | wedge  wedge  wedge  wedge  wedge  wedge | cordiate  cordiate  cordiate  reniform  reniform  reniform | ½  ½  ½  ½  ½  ½ | 2/3  2/3  2/3  ¾  2/3  2/3 | 3.0-3.5 × 2.0-2.5  3.0-3.5 × 2.0-2.5  3.0-3.5 × 2.0-2.5  3.5-5.0 × 3.0-3.5  3.5-5.0 × 3.0-5.0  4.5-5.5 × 3.0-3.1 |

Note: from one to five specimens for a species were analyzed.
