# Supplementary material for: What is Atraphaxis L. (Polygonaceae, Polygoneae): cryptic taxa and resolved taxonomic complexity instead of the formal lumping and the lack of morphological synapomorphies
Source: PeerJ. 2016 May 3;4:e1977. doi: 10.7717/peerj.1977 (PMC4860328; doi:10.7717/peerj.1977)
Supplement: Supplemental Information 5 [file peerj-04-1977-s005.doc]

**Table S5. Some perianth characteristics of the members of the tribe Polygoneae**

| Taxon and source | Perianth shape in flowering | Segments shape | Outer segments apex | Segments in fruiting | Perianth tube shape | Perianth tube length, mm | Stigmata |
| --- | --- | --- | --- | --- | --- | --- | --- |
| *Atraphaxis* (own observations) | campanulate with a long filiform tube | ovate, rotundate, cordiate or reniform | obtuse, flat or undulate at margin | inner segments accrescent, surrounding the achene; outer segments reflected or spreading | filiform with wedge-shaped or cup-shaped extention | 1.0–6.0 | fimbriate to capitate |
| *Bactria lazkovii* (own observations) | campanulate | ovate or elliptical | obtuse, plain | nonaccrescent, all surrounding the achene | cup-shaped | 0.3 | capitate |
| *Bactria ovczinnikovii* (own observations) | campanulate | lanceolate, with keeled midvein | acuminate or obtuse, cucullate | non-accrescent, all surrounding the achene | funnel-shaped | 0.3 | capitate |
| *Duma*  (Bradbyge 1992; Schuster, Wilson & Kron, 2011) | campanulate | ovate to broadly ovate | acuminate to rounded, plain | non-accrescent, all surrounding the achene | Funnel-form, widely campanulate | 1.0–1.5 | fimbriate |
| *Fallopia convolvulus* (own observations) | campanulate with a long or short tube | elliptical, outer segments with keeled or winged midvein | obtuse to acuminate, cucullate | Three outer segments accrescent, surrounding the achene | filiform with wedge-shaped extention | 0.5–8.0 | Capitate, smooth or papillate |
| *Knorringia*  (Hong, 1998) | campanulate | Oblong-ovate | obtuse, plain | non-accrescent | funnel-form | 1.0 | capitate |
| *Muehlenbeckia*  (Brandbyge, 1992) | campanulate | Ovate to broadly-ovate | acuminate to rounded, plain | slightly accrescent and succulent | funnel-form, widely campanulate | 0.5–1.0 | fimbriate |
| *Reynoutria* (own observations) | campanulate with a long tube | Obovate to elliptical, outer segments with keeled or winged midvein | obtuse to acuminate, cucullate | 3 outer segments accrescent, surrounding the achene; | filiform with wedge-shaped extention | 2.0–3.0 | fimbriate |
| *Polygonella*  (Horton, 1963) | campanulate | oblong to suborbicular | obtuse, plain | non-accrescent, all surrounding the achene | filiform with wedge-shaped extention | 0.1–3.0 | capitate |
| *Polygonum* (own observations) | campanulate or urceolate | ovate | obtuse or acuminate, plain to cucullate | non-accrescent, tightly surrounding the achene | funnel-form to cup-shaped | 0.3–0.5 | capitate |
| *Polygonum salicorniloides*  (own observations) | urceolate | oblong-elliptical or lanceolate | oblong-elliptical or lanceolate | non-accrescent, tightly surrounding the achene | funnel-form | 0.3 | capitate |
