# Supplementary material for: What is Atraphaxis L. (Polygonaceae, Polygoneae): cryptic taxa and resolved taxonomic complexity instead of the formal lumping and the lack of morphological synapomorphies
Source: PeerJ. 2016 May 3;4:e1977. doi: 10.7717/peerj.1977 (PMC4860328; doi:10.7717/peerj.1977)
Supplement: Supplemental Information 6 [file peerj-04-1977-s006.doc]

**Table S6. Main pollen characteristics in the tribe Polygoneae**

| Taxon | Palynotype | P/E | Pollen shape | Exine μm | Sporoderm surface | Reference |
| --- | --- | --- | --- | --- | --- | --- |
| *Atraphaxis* | Atraphaxis | 1.05–1,34 | spheroidal to oblong-spheroidal | 1.6–2.4 | striato-foveolate to striato-perforate with distinct or smoothened striae and small perforations in rows in grooves between the striae | Hong, 1995; Yurtseva, Severova & Bovina, 2014 |
| *Bactria lazkovii* | Lazkovia | 1.13 | speroidal to oblong-spheroidal | 1.66 | Foveolate-perforate with 4-6 angular or rounded pits 0.5-1.5 µm in diam., and rare perforations 0.5–1.5 µm in diam., single or two at the lumina | This paper |
| *Bactria ovczinnikovii* | Bactria | 1.18 | speroidal to oblong-spheroidal | 1.7 | Microreticulate-foveolate with 4–6 angular pits, rarely with small perforations less then 0.2 µm in diam. | This paper |
| *Duma* | Avicularia | 1.0–1.1 | subprolate to prolate | 1.5 | microfoveolate, microspinulose | Brandbyge, 1992 |
| *Fallopia convolvulus* | Fallopia | 1.00–1.43 | Prolate to spheroidal | 2.0–3.5 | microspinulose around the colpi and psilate or punctate at mesocolpia and poles | Nowicke & Skvarla, 1979, Van Leeuwen, Punt & Hoen, 1988 |
| *Knorringia* | — | 1.06–1.67 | prolate to subprolate or spheroidal | 2–2.8 | reticulate with rugulose muri, ± regular or irregular lumina in shape and size | Hong, 1989 |
| *Muehlenbeckia* | — | 1.0–1.1 | Prolate to subprolate | 1.5 | micropunctate to microreticulate with lumina angular or rounded (Nowicke and Skvarla 1977), punctate-striate (Brandbyge, 1992; Nowicke and Skvarla 1977; 1979); microfoveolate, sparsely spinulose (Nowicke and Skvarla 1977) | Nowicke & Skvarla, 1977; Nowicke & Skvarla, 1979; Brandbyge, 1992 |
| *Polygonum* sect *Polygonum* | Avicularia | 1.04–1.75 | subprolate or prolate-spheroidal | 1.2–3.7 | psilate, micropunctate and microspinulose | Hong, Oh &, Ronse De Craene, 2005 |
| *Polygonum* sect. *Tephis* | Avicularia | 1.21–1.41 | subprolate | 2.5–3.5 | psilate, micropunctate, microspinulate | Hong, Oh & Ronse De Craene, 2005 |
| *Polygonum* sect. *Pseudomollia* | Pseudomollia | 1.24–1.42 | subprolate to prolate | 1.5–2.3 | psilate around the colpi, verrucate at 1/3 mesocolpia and poles | Hong, Oh & Ronse De Craene, 2005 |
| *Polygonum* sect. *Duravia* | Duravia | 1.28–1.96 | subprolate to prolate | 1.5–5.5 | rugulate or foveolate with microspinules around the colpi, semitectate-reticulate at mesocolpia | Hong, Oh & Ronse De Craene, 2005 |
| *Polygonella* | Duravia | 1.34–1.83 | prolate | 1.0–3.3 | rugulate-reticulate with microspinules around the colpi, semitectate-reticulate at mesocolpia | Hong, Oh & Ronse De Craene, 2005 |
| *Polygonum salicornioides, P. aridum* | — | — | prolate to subprolate |  | Reticulato-perforate | Tavakkoli et al., 2015 |
