# Supplementary material for: What is Atraphaxis L. (Polygonaceae, Polygoneae): cryptic taxa and resolved taxonomic complexity instead of the formal lumping and the lack of morphological synapomorphies
Source: PeerJ. 2016 May 3;4:e1977. doi: 10.7717/peerj.1977 (PMC4860328; doi:10.7717/peerj.1977)
Supplement: Supplemental Information 7 [file peerj-04-1977-s007.doc]

**Data S3. Origin of the material used for the SEM micrographs.**

***Atraphaxis* *ariana* ( Grigorj.) T.M.Schust. & Reveal —** Turkmenskaya SSR, [Badghyz, Kushka d.,] vill. Morgunovsky, 25.04.1988. s.n. Gorelova (LE); Turkmenistan, [Badghyz, Kushka d.,], 3 km to SE of the railway station Morgunovka, 15.04.1952. *Zhudova, Gruzdeva s.n.* (LE).

***Atraphaxis* *atraphaxiformis* ( Botsch.) T.M.Schust. & Reveal** — Tajikistan, Turkestan ridge, N slope, Isphara d., 4-5 km to SE of Vorukh, 1653 m a.s.l. Juniperus forest at river Kshemysh, 14.06.1962. *Filatov 174*, (LE).

***Atraphaxis avenia* Botsch. —** South Tajikistan, Mts surrounding village Bag on the river Pjandzh, red sandstones at the right bank of the river Shpilyau. 1.06.1960. *Botschantzev, Egorova, 779*. (LE).

***Atraphaxis badghysi* Kult. —** Turkmenistan, E Kara-Kum, Tej-Murgab interfluve, 15 km to S from Kureli to Gumbezli. 4.05.1956. *Akimuradov s.n.* (LE).

***Atraphaxis caucasica* (Hoffm.) Pavlov** — Georgia, Mtskheta d., in cliff Armazi. 17.06.1969. *Grebenschikov et al. s.n.* (MW).

***Atraphaxis frutescens* (L.) C.Koch** — Mongolia, the Mongol Altay, Bayan-Olуgiy aimag, 15 km to N of Bulgan. Mt. slope in the estuary of the Ikh-Dzhegalanta, right inflow of the river Bulgan, 2100 m a.s.l. 27.08.1983. *Gubanov 7281*. (MW); SW Mongolia, Dzungaria, Khovd aimag, N slope of Baitag-Bogdo Ridge, the lower part of the canyon Budun-Khargaityn-Gol, 15 km to E of the outpost Baitag-Bogdo. 1800-2000 m a.s.l. 30.07.1988. *Gubanov et al. 2551*. (MW).

***Atraphaxis kopetdagensisKovalevsk.—***Turkmenistan, Central Kopet-Dagh, Geok-Tepe d., Kara-Agach. 30.05.1972. *Mescheryakov* (LE)

***Atraphaxis laetevirens* (Ledeb.) Jaub. et Spach** — Kazakhstan, Talas Alatau, Aksu-Dzhabagly, pass Kshy-Kaindy. Above Akshy-Aksu, S slopes. 11.08.1948. *Kultiasov s.n.* (MW).

***Atraphaxis manschurica Kitag***. China, the Inner Mongolia, between u. Tunlyon and u. Dunkehoutsy, vill. Shuan-Fumiao. 23.06.1951. *Lion 3156.* (LE); Inst. Bot. Acad. Sinicae. 2008. *Hortus Botanicus Pekinensis 256*.

***Atraphaxis muschketowi* Krasn. —** Kazakhstan, Tien Shan, Zaily Alatau, near Alma-Ata, Mt. Kok-Tebe. 24.05.1998. *Majorov 98-24a.* (MW); Kazakhstan, N Tien Shan, Zaily Alatau, Alma-Ata, 17.06.1933. *Popov 4260* (LE).

***Atraphaxis pungens* (Bieb.) Jaub. et Spach** — Siberia, Tuva ASSR, Ulug-Khem d., Ridge Uyuk, stony steppe near Bayan-Kolsky parom. 6.08.1976. *Lomonosova, Ivanova 2408*. (MW); Krasnoyarsky kray, Khakasia, 12 km to SW of Charkov. 1.07.1953. *Polozhyi et al. s.n.* (MW).

***Atraphaxis replicata* Lam**. — Daghestan, Buinaksk d., Kumtorkale. 29.09.1963. *Rotov s.n.* (MW).

***Atraphaxis seravschanica* Pavlov** — Kyrgyzstan, Ketmen-Tyube basin, Susamyr Ridge, Kapchichay, between Sary-Sogot and Torkent. 8.06.1960. *Botbaeva s.n.* (MW); Uzbekistan, West Tian Shan, south spures of the Chatkal Ridge, Angren Ridge, 40 km to NE of Mt Angren. 16.05.1965. *Boryaev and Gubanov 43*. (MW).

***Atraphaxis teretifolia* (M.Pop.) Kom. ex Pavl.** — Kazakhstan, Karaganda reg., Pribalkhashye, N of the bay Sar-Tchagan. 26.05.1951. *Pavlov 333.* (MW).

***Atraphaxis* *toktogulica* (Lazkov ) T.M.Schust. & Reveal —** Kyrgyzstan, Toktogul d., Karajigach, left board of say Tor-Kolot. 5.07.1973. *Ajdarova et al. s.n.* (LE, Holotypus).

***Atraphaxi*s *tournefortii* Jaub. et Spach —** Persia, Azerbaijan, 26 km W of Rezaiyeh to Serow. Green Centre 1600-1700 m a.s.l. 14.06.1971. *Lamond 4137* (LE).

***Atraphaxis virgata* (Regel.) Krasn. —** Mongolia, Khovd aimag, Dzhungar Goby, 45 km to W of the well Takhi-Us. 30.07.1979. *Gubanov 7227*. (MW); Mongolia, Altay Goby, foothills of Atas-Bogdo, 15 km N of Del-Ula. 3.08.1978. *Ogureeva 171*. (MW).

***Bactria lazkovii* O.V.Yurtseva** Kyrgyzstan, Naryn reg., Dzhumgal d., Kavak-Too Ridge, Sary-Bulun. 7.07.2006. *Lazkov 24.* (MW).

***Bactria ovczinnikovii* (Czukav.) O.V.Yurtseva & E.V.Mavrodiev—** South Tajikistan, vill. Bag at the river Pyandzh, red and gray sandstones on the left bank of the Aarzy-Su. 2.06.1960. *V.Botschantzev & T. Egorova # 814*. (LE).
