# Supplementary material for: What is Atraphaxis L. (Polygonaceae, Polygoneae): cryptic taxa and resolved taxonomic complexity instead of the formal lumping and the lack of morphological synapomorphies
Source: PeerJ. 2016 May 3;4:e1977. doi: 10.7717/peerj.1977 (PMC4860328; doi:10.7717/peerj.1977)
Supplement: Supplemental Information 8 [file peerj-04-1977-s008.doc]

**Data S4. Origin of the material used for LM micrographs**

***Atraphaxis* *ariana* ( Grigorj.) T.M.Schust. & Reveal —** Turkmenistan, [Kushka d.], foothills near the fortress Kushka. 17.05.1934. *Androsov* (LE); Turkmenistan, Badghyz, at the Kushka pass, 2-3 km W of vil. Poltavsky. 3.06.1949. *Nikitin* (LE).

***Atraphaxis* *atraphaxiformis* (**[**Botsch.**](http://www.ipni.org/ipni/idAuthorSearch.do?id=1010-1&back_page=%2Fipni%2FeditAdvPlantNameSearch.do%3Ffind_infragenus%3D%26find_isAPNIRecord%3Dtrue%26find_geoUnit%3D%26find_includePublicationAuthors%3Dtrue%26find_addedSince%3D%26find_family%3D%26find_genus%3DAtraphaxis%26find_sortByFamily%3Dtrue%26find_isGCIRecord%3Dtrue%26find_infrafamily%3D%26find_rankToReturn%3Dall%26find_publicationTitle%3D%26find_authorAbbrev%3D%26find_infraspecies%3D%26find_includeBasionymAuthors%3Dtrue%26find_modifiedSince%3D%26find_isIKRecord%3Dtrue%26find_species%3D%26output_format%3Dfull)**)** [**T.M.Schust.**](http://www.ipni.org/ipni/idAuthorSearch.do?id=20018666-1&back_page=%2Fipni%2FeditAdvPlantNameSearch.do%3Ffind_infragenus%3D%26find_isAPNIRecord%3Dtrue%26find_geoUnit%3D%26find_includePublicationAuthors%3Dtrue%26find_addedSince%3D%26find_family%3D%26find_genus%3DAtraphaxis%26find_sortByFamily%3Dtrue%26find_isGCIRecord%3Dtrue%26find_infrafamily%3D%26find_rankToReturn%3Dall%26find_publicationTitle%3D%26find_authorAbbrev%3D%26find_infraspecies%3D%26find_includeBasionymAuthors%3Dtrue%26find_modifiedSince%3D%26find_isIKRecord%3Dtrue%26find_species%3D%26output_format%3Dfull) **&** [**Reveal**](http://www.ipni.org/ipni/idAuthorSearch.do?id=8314-1&back_page=%2Fipni%2FeditAdvPlantNameSearch.do%3Ffind_infragenus%3D%26find_isAPNIRecord%3Dtrue%26find_geoUnit%3D%26find_includePublicationAuthors%3Dtrue%26find_addedSince%3D%26find_family%3D%26find_genus%3DAtraphaxis%26find_sortByFamily%3Dtrue%26find_isGCIRecord%3Dtrue%26find_infrafamily%3D%26find_rankToReturn%3Dall%26find_publicationTitle%3D%26find_authorAbbrev%3D%26find_infraspecies%3D%26find_includeBasionymAuthors%3Dtrue%26find_modifiedSince%3D%26find_isIKRecord%3Dtrue%26find_species%3D%26output_format%3Dfull) — Tajikistan, N slope of Turkestan Ridge, Isphara d., 4-5 km to SE of Vorukh, h=1653 m a.s.l. juniper stand at the river Kshemysh. 14.06.1962. *Filatov 174*. (LE); Tajikistan, Turkestan ridge, the Isphara basin, above vil. Vorukh, rocks. 07.1970. *Kamelin 532*. (LE) (=43)

***Atraphaxis badghysi* Kult.** Turkmenistan, lake Er Oylan-Duz. 21.04.1965. Mesheryakov (LE)

***Atraphaxis fischeri* Jaub & Spach.** – Russia, Volgograd reg., Kirov d., Bolshaya Otrada. 7.05.09. *Klinkova, Suprun 2.* (MW); Russia, Astrakhan prov., Mt Bolshoe Bogdo, Mt near lake Baskunchak, south slopes. 25.05.1990. *G.Klinkova et al.* (MHA)

***Atraphaxis spinosa* L.** — Armenia, Ararat reg., environs of the Mt. Goravan, the refuge «Goravan Sands», sandy slopes.13.08.2012. *D. Lyskov* (MW)

***Atraphaxis teretifolia* (M.Pop.) Kom. ex Pavl.** — Kazakhstan, Karaganda reg., the Balkhash lake, at bank N of the bay Sar-Tchagan. 26.05.1951. *Pavlov 333*. (MW); Kazakhstan, Karaganda reg., 40 km N of Dzheskazgan at the road to Ula-Tau. Limestone. 23.06.1958. *E.I.Rachkovskaya 6185*. (LE) (=142)

***Atraphaxis* *toktogulica* (Lazkov ) T.M.Schust. & Reveal —** Kyrgyzstan, Toktogul d., Karajigach, left board of say Tor-Kolot. 5.07.1973. *Ajdarova et al. s.n.* (LE, Holotypus).

***Atraphaxis tortuosa* Losinsk.** (=*Polygonum intramongolicum* Fu & Zhao; *=P.* *tortuosum* (Losinskaja) Lovelius) **—** Mongolia occ., the north band of the river Huang-He, Muni-Ula. ½.05.1872. *Przhewalsky* (LE, Isotypus); Mongolia, South Goby aimak, 22 km SE of somon Khan-Bogd. 23.06.1972. *E.I.Rachkovskaya, I.I.Guricheva. 0151*. (LE)

***Atraphaxis virgata* (Regel) Krasn.** — Kazakhstan, South Kazakhstan reg., Tyulkubass d., the riverhead of Mashat, “Mashat”, the break stone slopes nearby the river. 29.09.2012. *V.A. Sagalaev* (MHA). (=143)

***Bactria lazkovii* O.V.Yurtseva.** — Kyrgyzstan, Naryn reg., Dzumgal d., Kavak-Too Ridge, Sary-Bulun. 7.07.2006. *Lazkov 24*. (Holotypus, MW).

***Bactria ovczinnikovii* (Czukav.) O.V.Yurtseva (=*Polygonum ovczinnikovii* Czukav.)** — Southern Tajikistan, Schpilau, right bank of the river Piandzh, mountain slopes near Bag. 1.06.1960. *Nepli* (LE); Tajikistan**,** right band of the river Piandzh, between Bog and Bakhorak, 1100 m a. s. l., 2.06.1960, *Yunusov 1624***,** (LE).

***Polygonum salicornioides* Jaub. et Spach** — Persia australis. In alpe Kuh-Delu. 10 june 1842. *Kotschy 468* (Isotypus, LE).
