# Supplementary figures and images for: What is Atraphaxis L. (Polygonaceae, Polygoneae): cryptic taxa and resolved taxonomic complexity instead of the formal lumping and the lack of morphological synapomorphies
Source: PeerJ. 2016 May 3;4:e1977. doi: 10.7717/peerj.1977 (PMC4860328; doi:10.7717/peerj.1977)

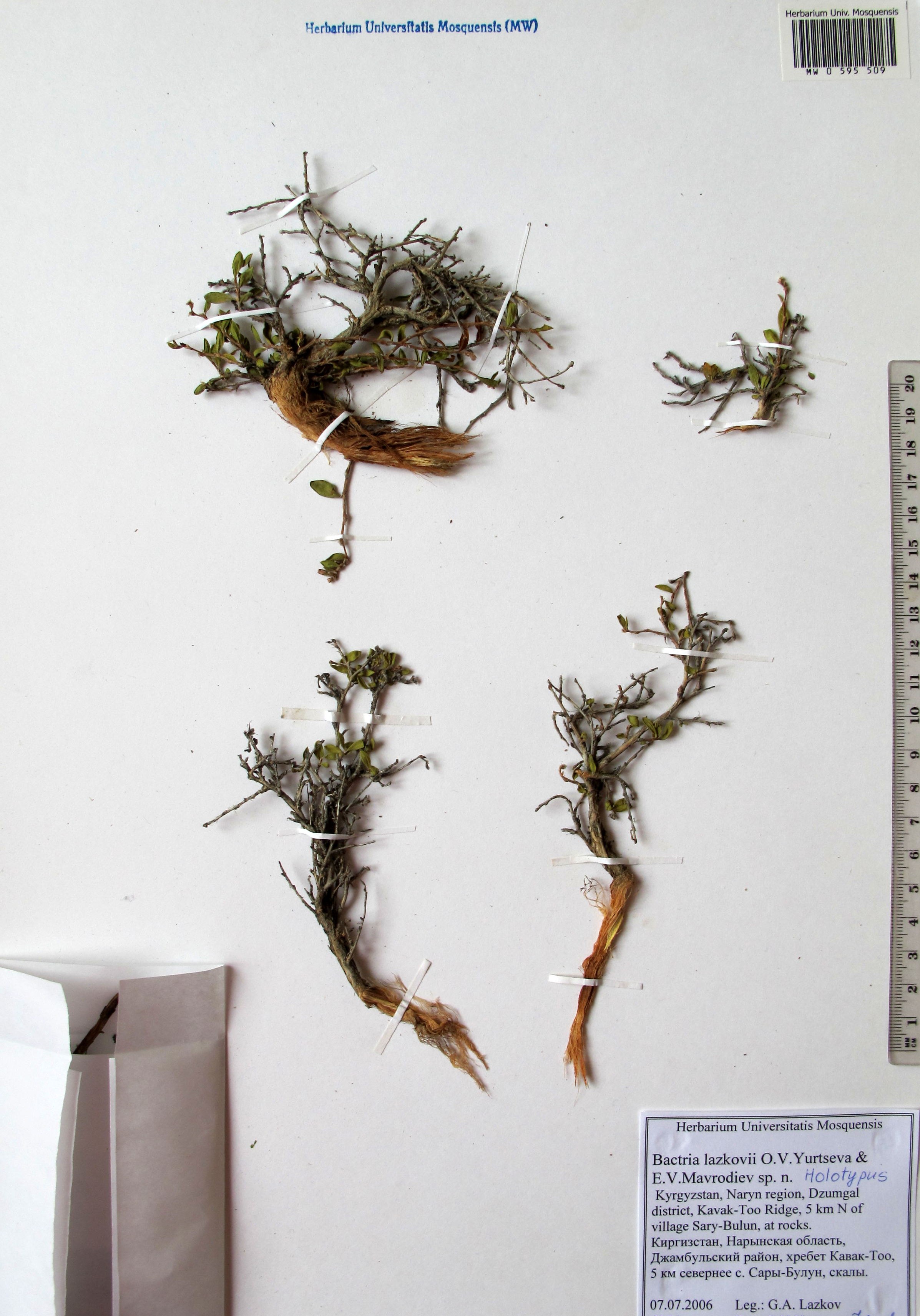

Supplement: Supplemental Information 9 — A dwarf shrub with divaricately branched shoots and frondulose terminal thyrses. Images: O. Yurtseva. [file peerj-04-1977-s009.png]

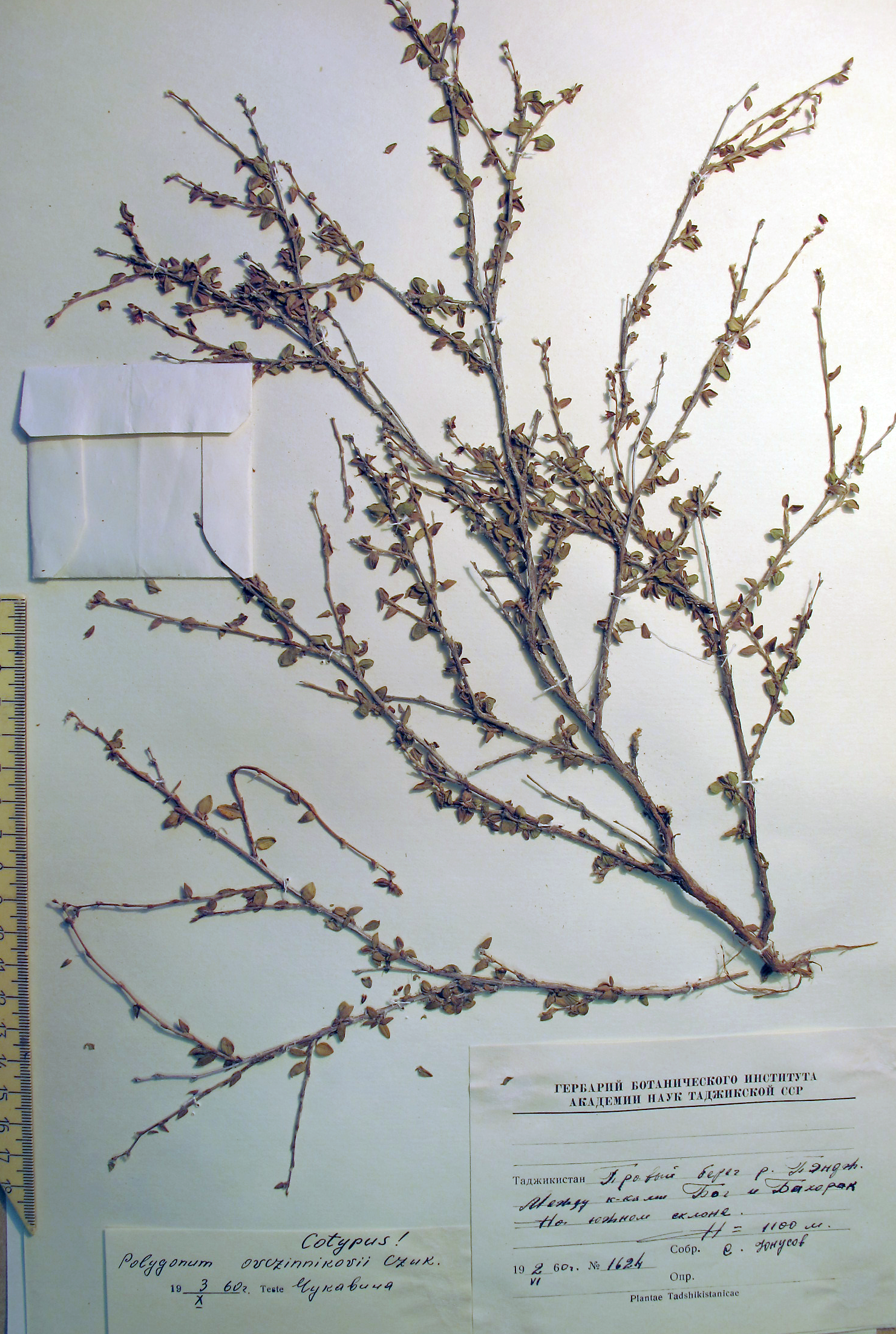

Supplement: Supplemental Information 10 — A dwarf shrub with divaricately branched shoots and frondulose terminal thyrses. Images: O. Yurtseva. [file peerj-04-1977-s010.png]

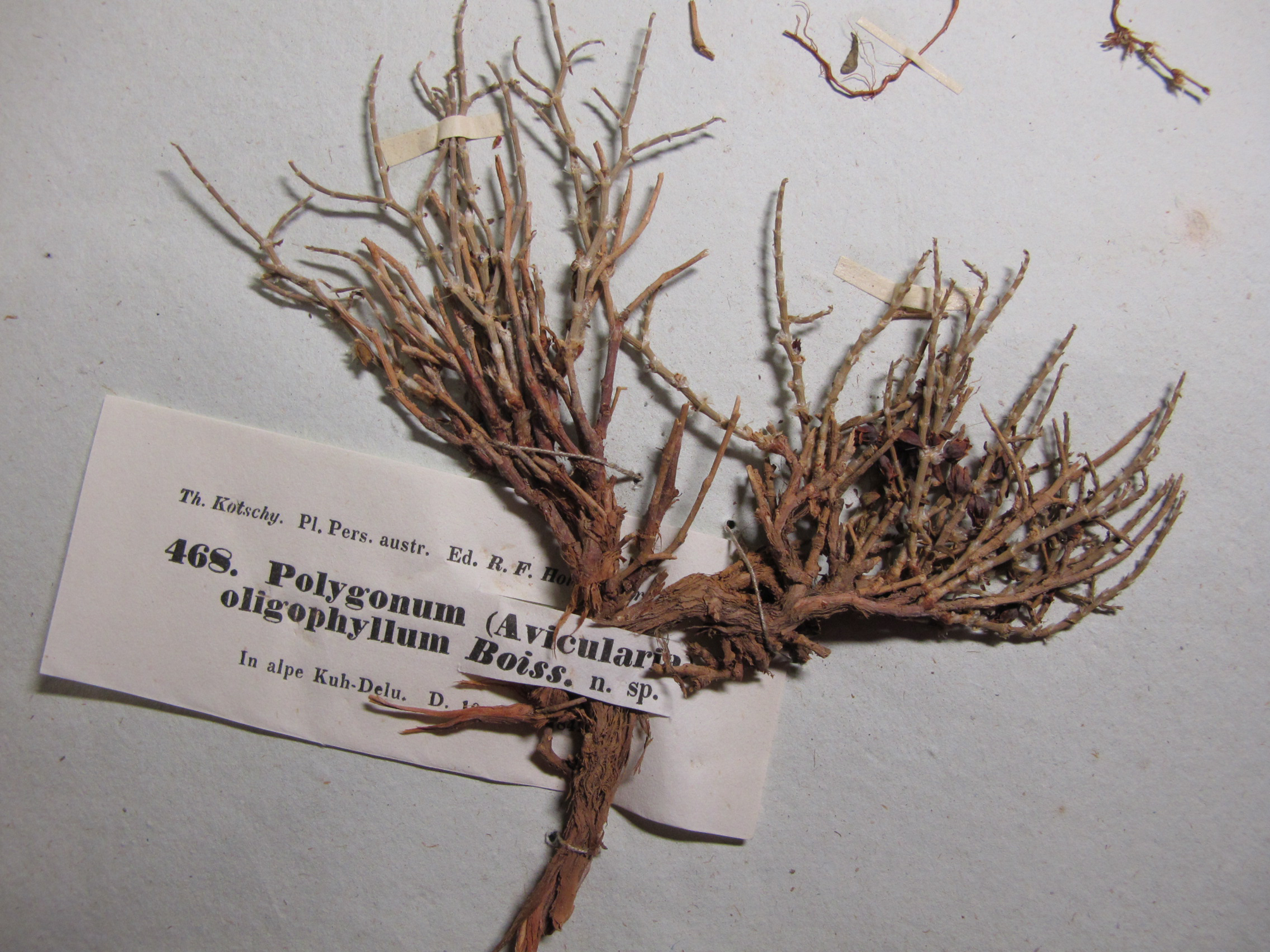

Supplement: Supplemental Information 11 — A dwarf undershrub with branched annual shoots and axillary cymes of flowers in axils of deciduous leaves. Images: O. Yurtseva. [file peerj-04-1977-s011.png]

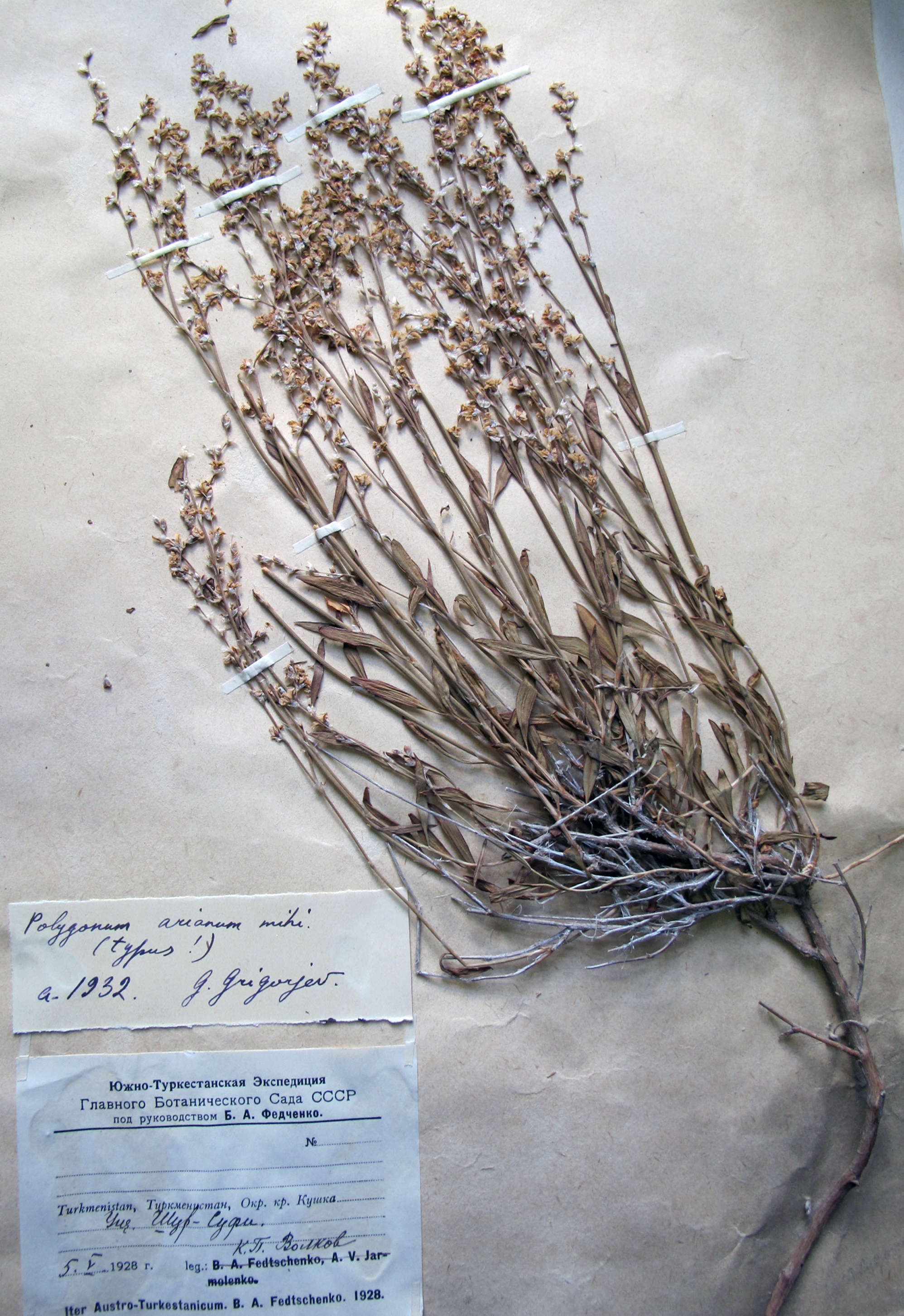

Supplement: Supplemental Information 12 — An undershrub or shrub with buried in sand manyheaded caudex and numerousl elongated generative shoots terminated by bracteose thyrses, or racemes of thyrses. Images: O. Yurtseva. [file peerj-04-1977-s012.png]

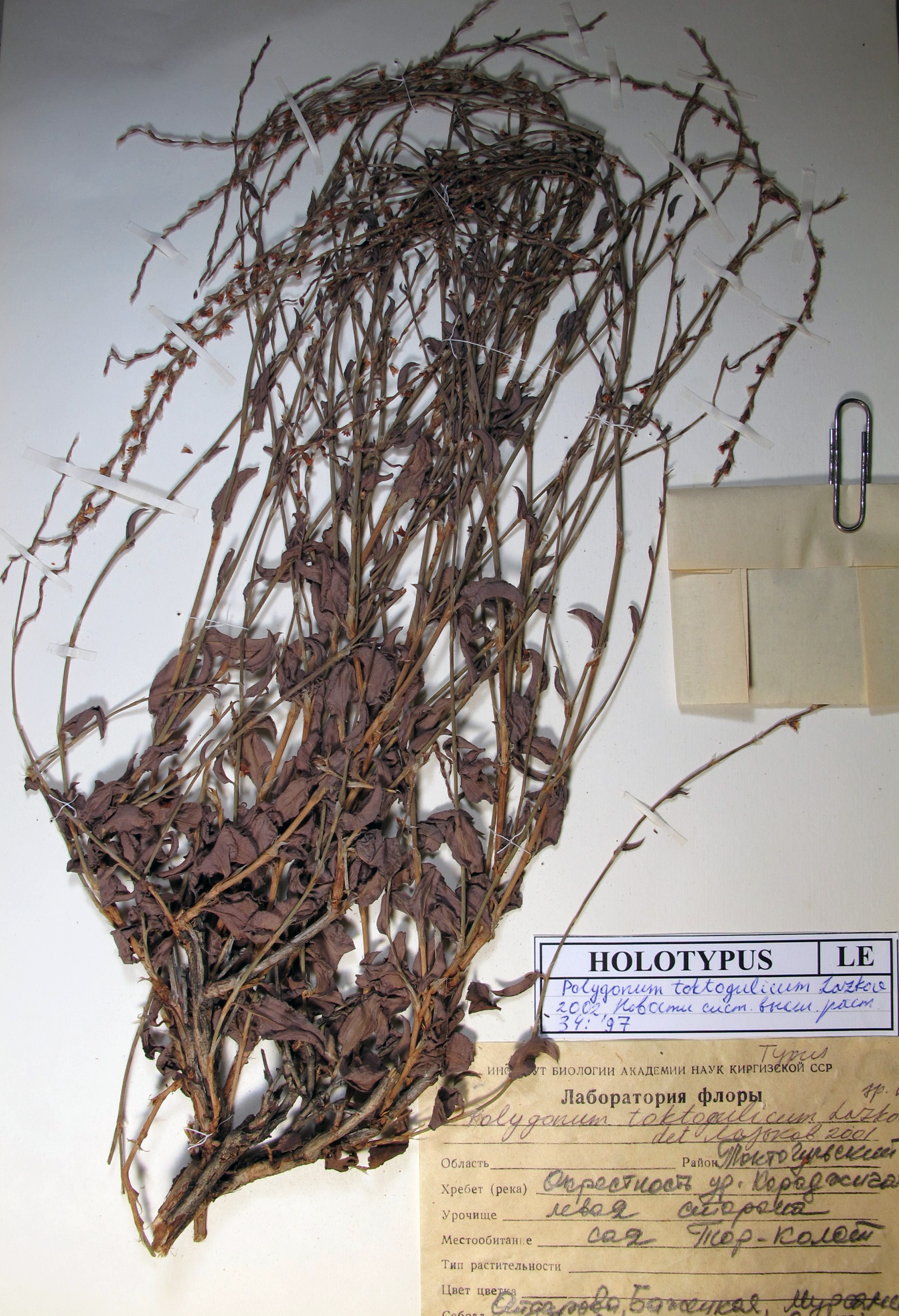

Supplement: Supplemental Information 13 — An undershrub or shrub with manyheaded caudex and numerous elongated generative shoots terminated by bracteose thyrses or racemes of thyrses. Images: O. Yurtseva. [file peerj-04-1977-s013.png]

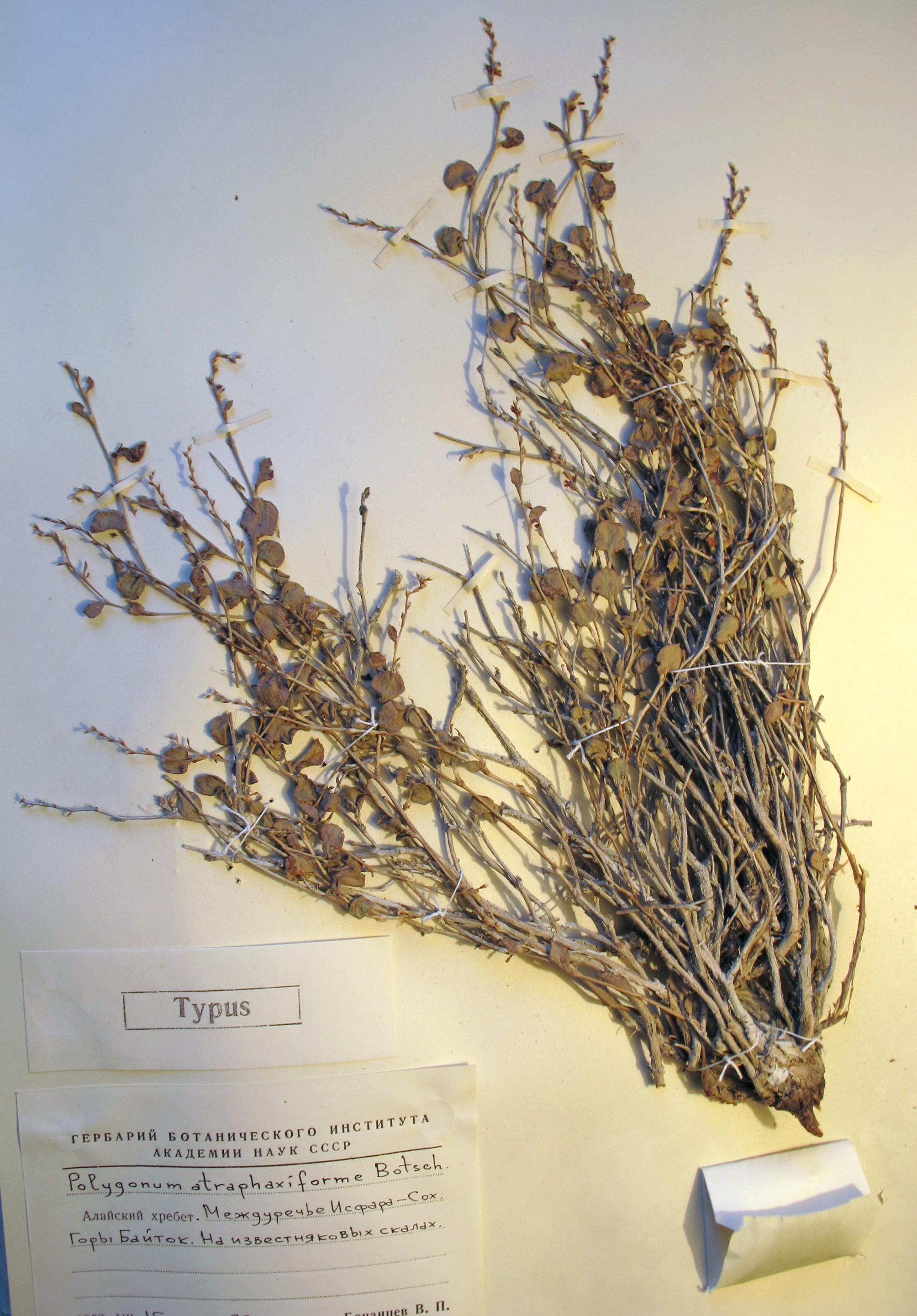

Supplement: Supplemental Information 14 — A shrub with elongated intensively branched shoots terminated by bracteose thyrses. Images: O. Yurtseva. [file peerj-04-1977-s014.png]

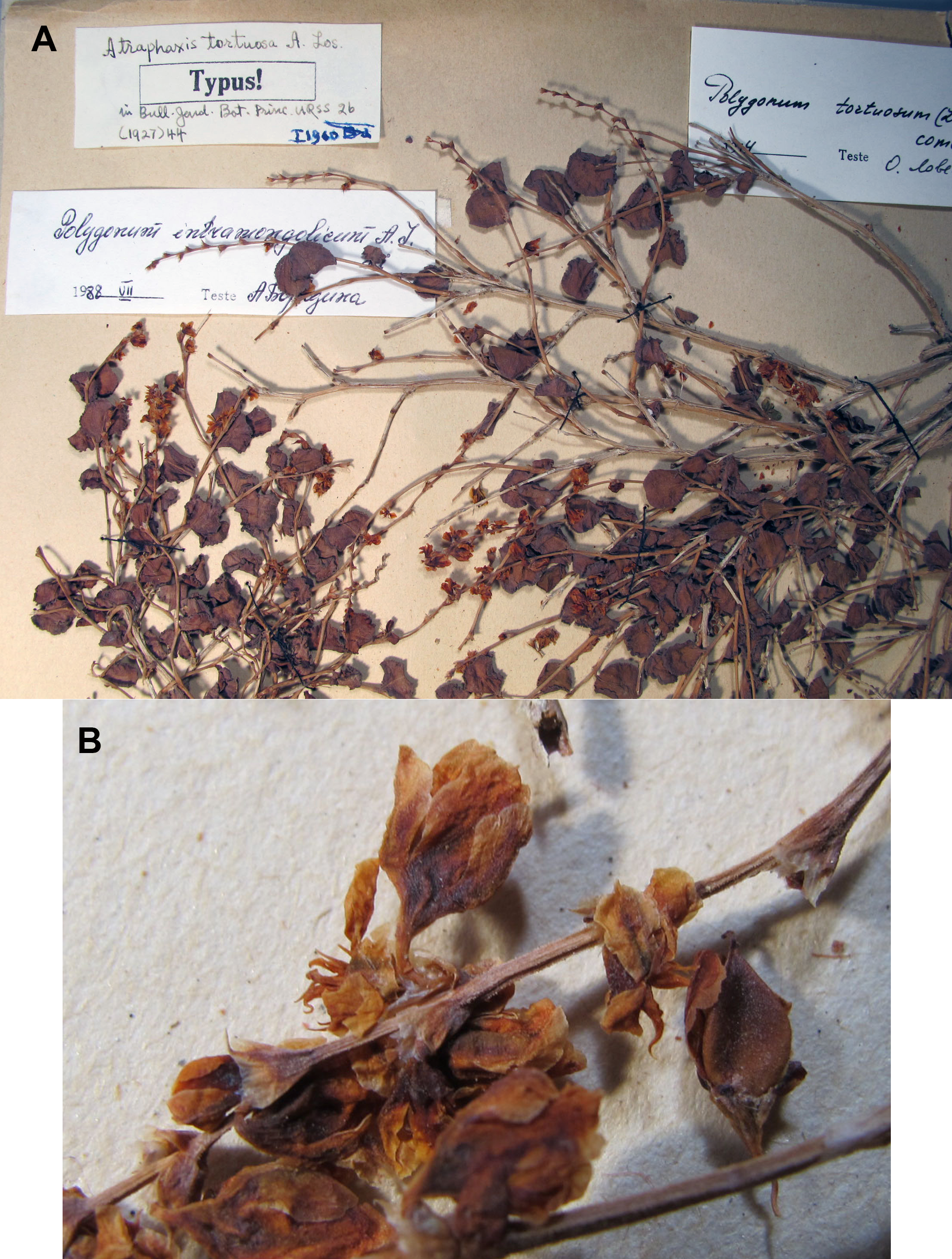

Supplement: Supplemental Information 15 — (A) A shrub with elongated intensively branched shoots, terminated by bracteose thyrses (above). (B) A fragment of bracteose thyrse with axillary cymes of flowers and the achene (below). Images: O. Yurtseva. [file peerj-04-1977-s015.png]

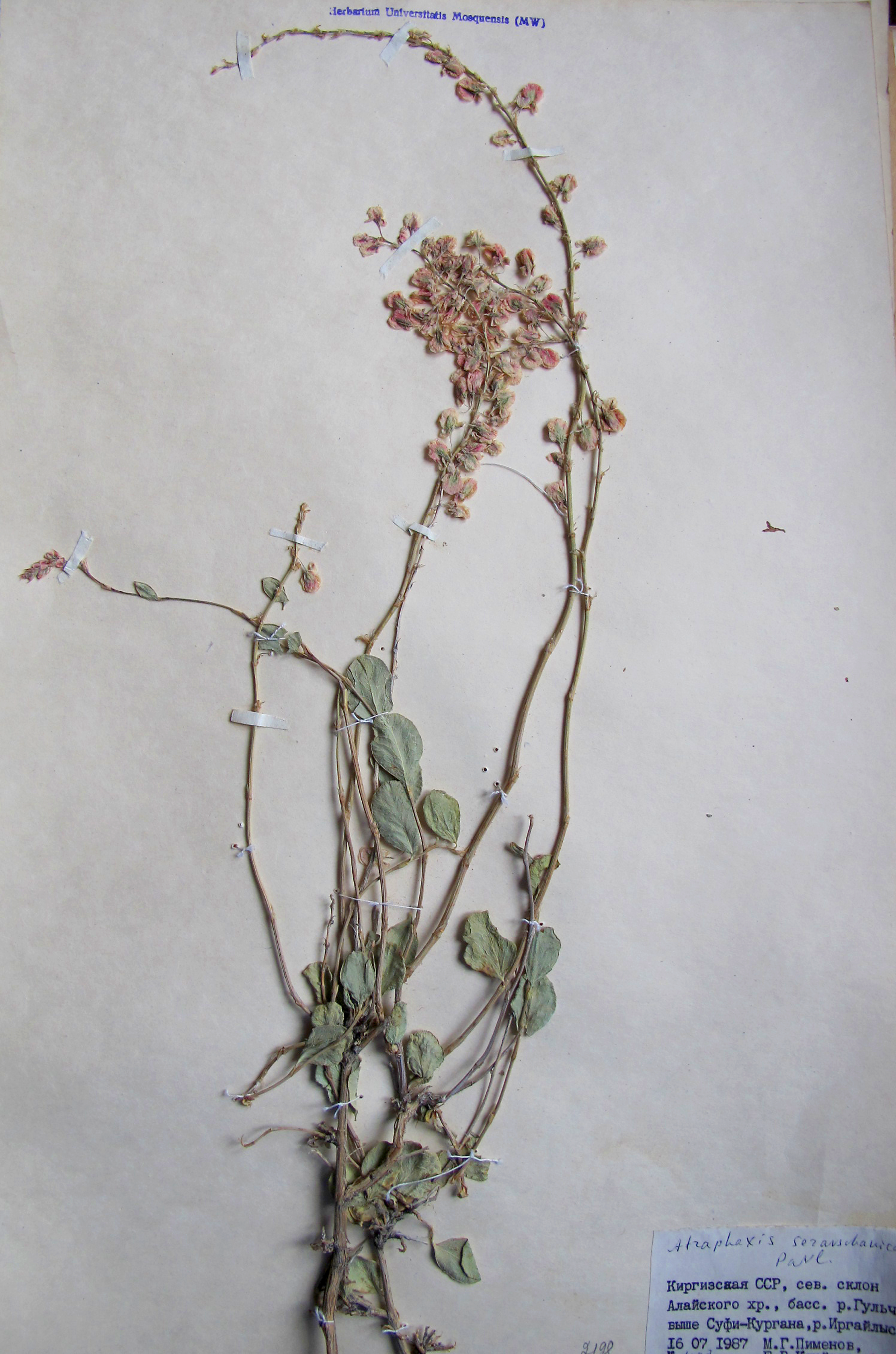

Supplement: Supplemental Information 16 — A shrub with elongatated generative shoots terminated by bracteose thyrses. Images: O. Yurtseva. [file peerj-04-1977-s016.png]

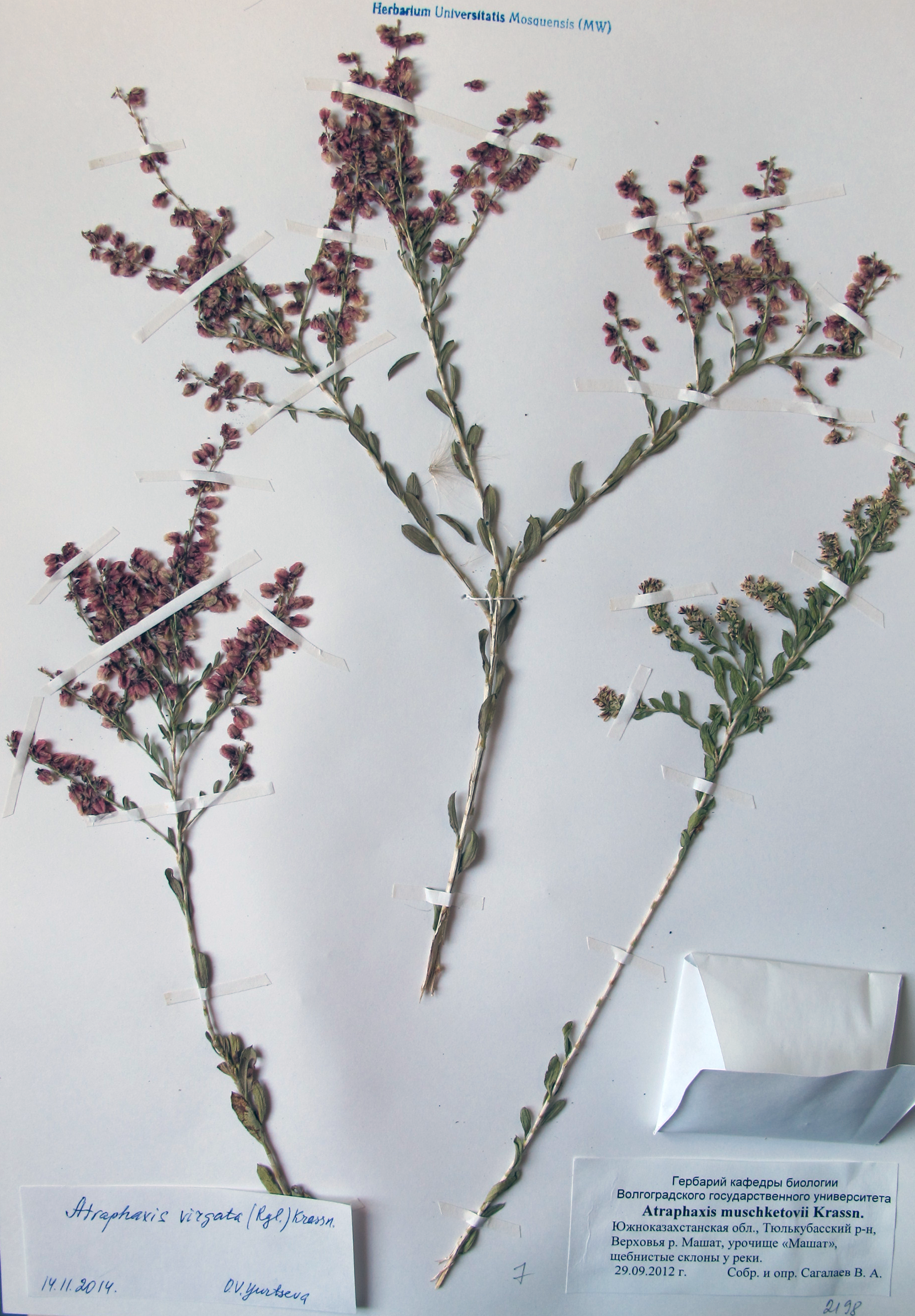

Supplement: Supplemental Information 17 — A shrub with elongated generative annual shoots each terminated by a raceme of bracteose thyrses, the central shoot with two paracladia. Images: O. Yurtseva. [file peerj-04-1977-s017.png]

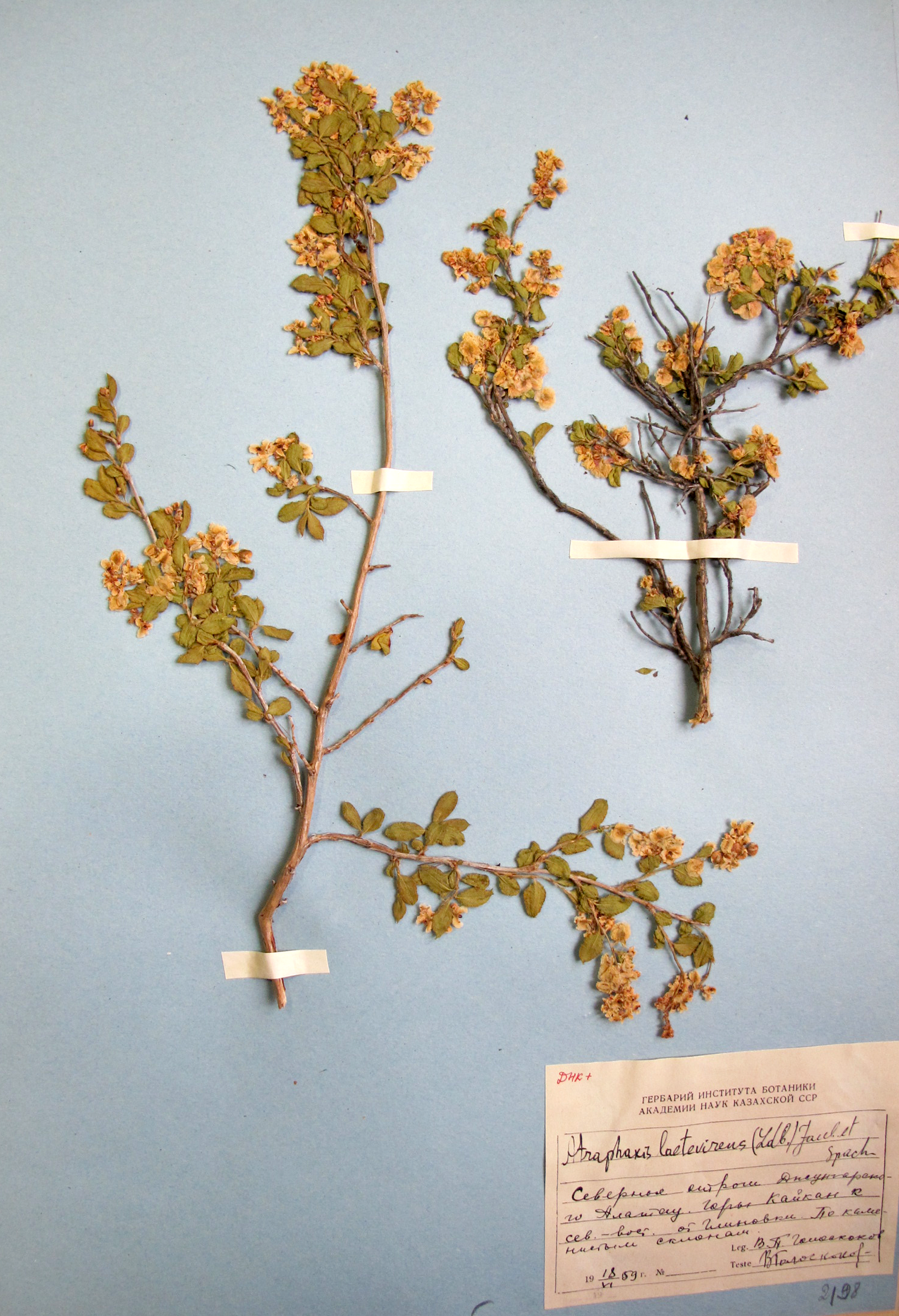

Supplement: Supplemental Information 18 — A shrub with lateral abracteose thyrses. Images: O. Yurtseva. [file peerj-04-1977-s018.png]

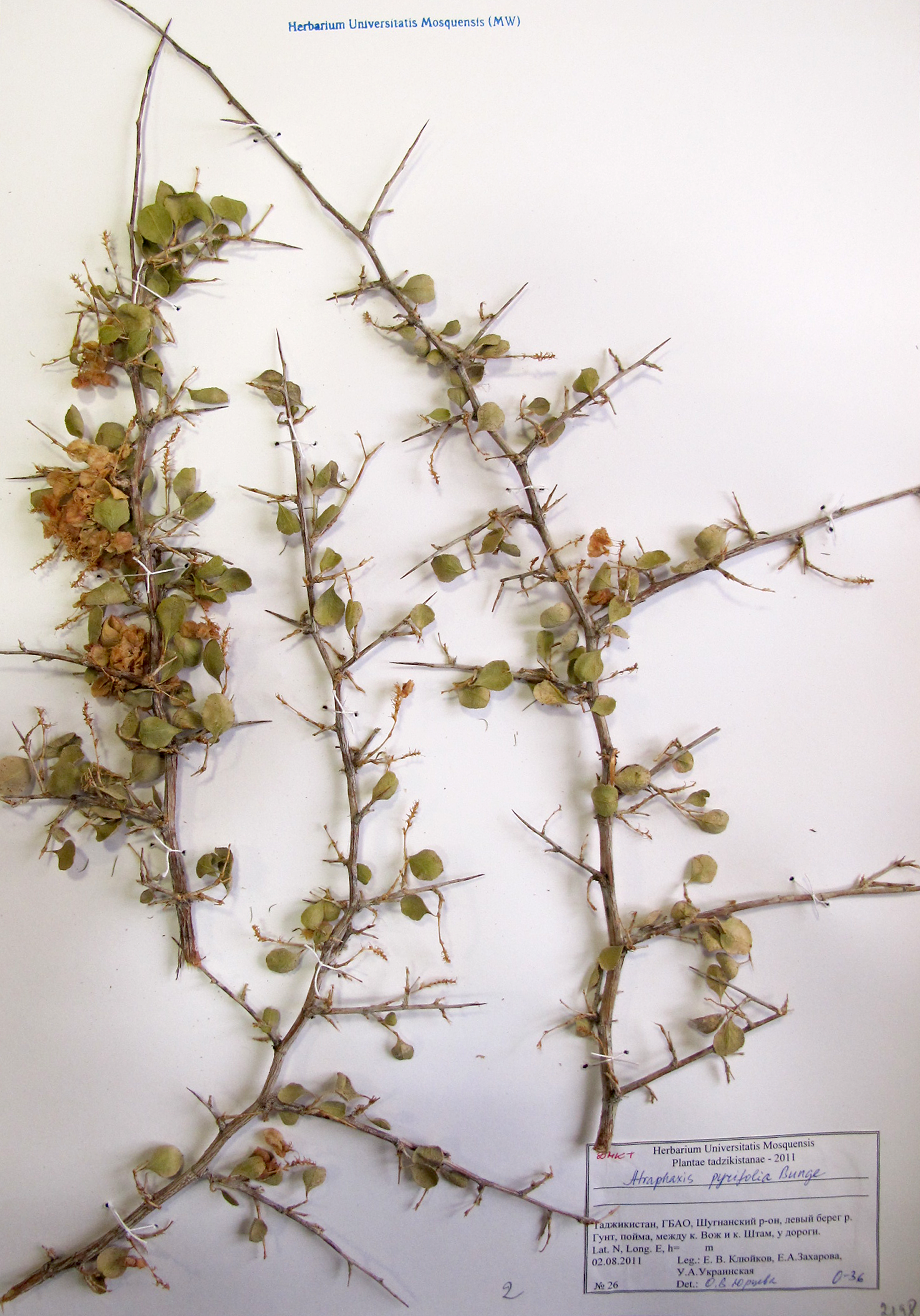

Supplement: Supplemental Information 19 — A shrub with elongated prickly shoots and second-order branchlets, and congested thyrses lateral at the second-year branchlets. Images: O. Yurtseva. [file peerj-04-1977-s019.png]

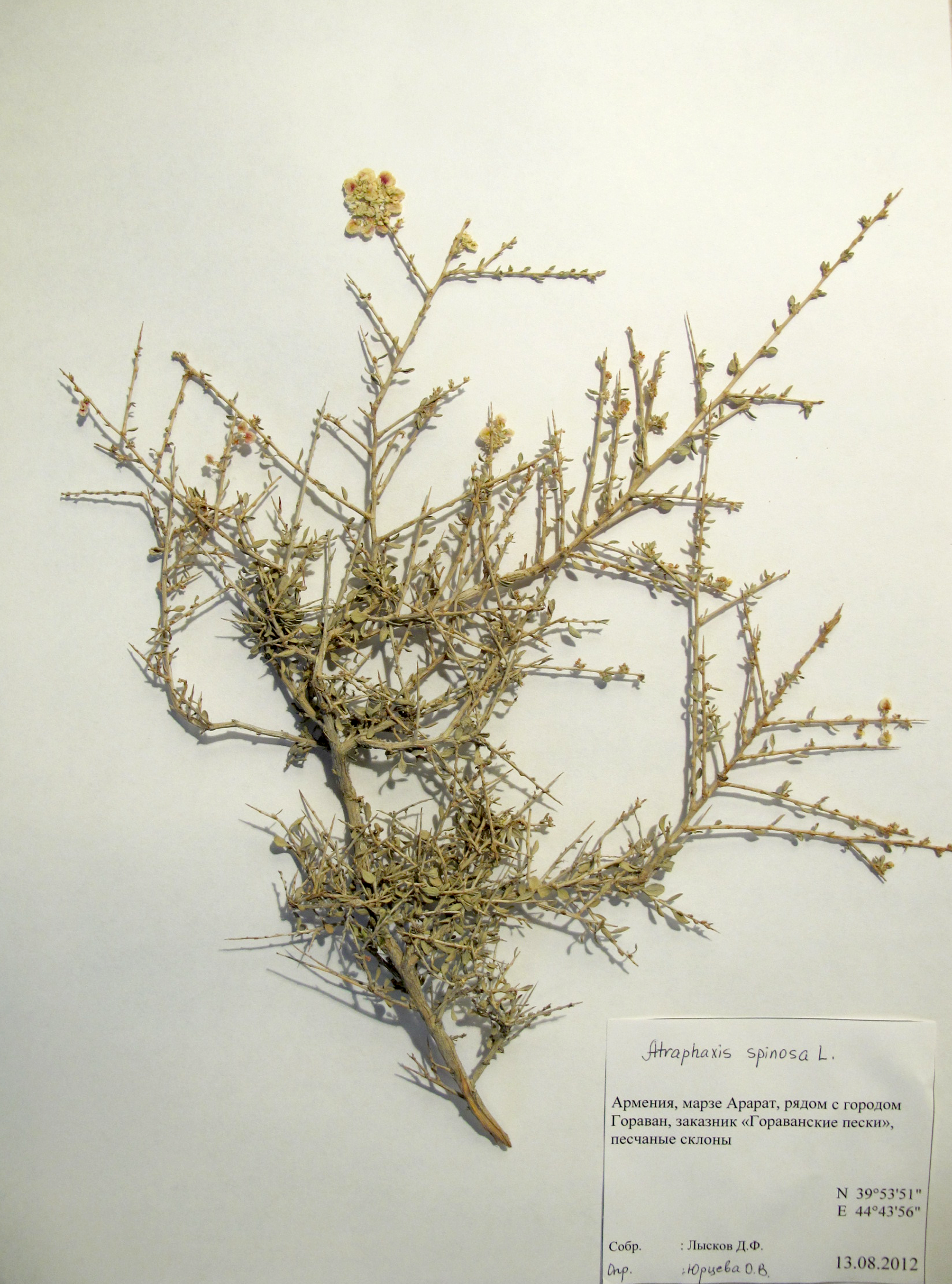

Supplement: Supplemental Information 20 — A shrub with prickly elongated shoots and branchlets. Images: O. Yurtseva. [file peerj-04-1977-s020.png]

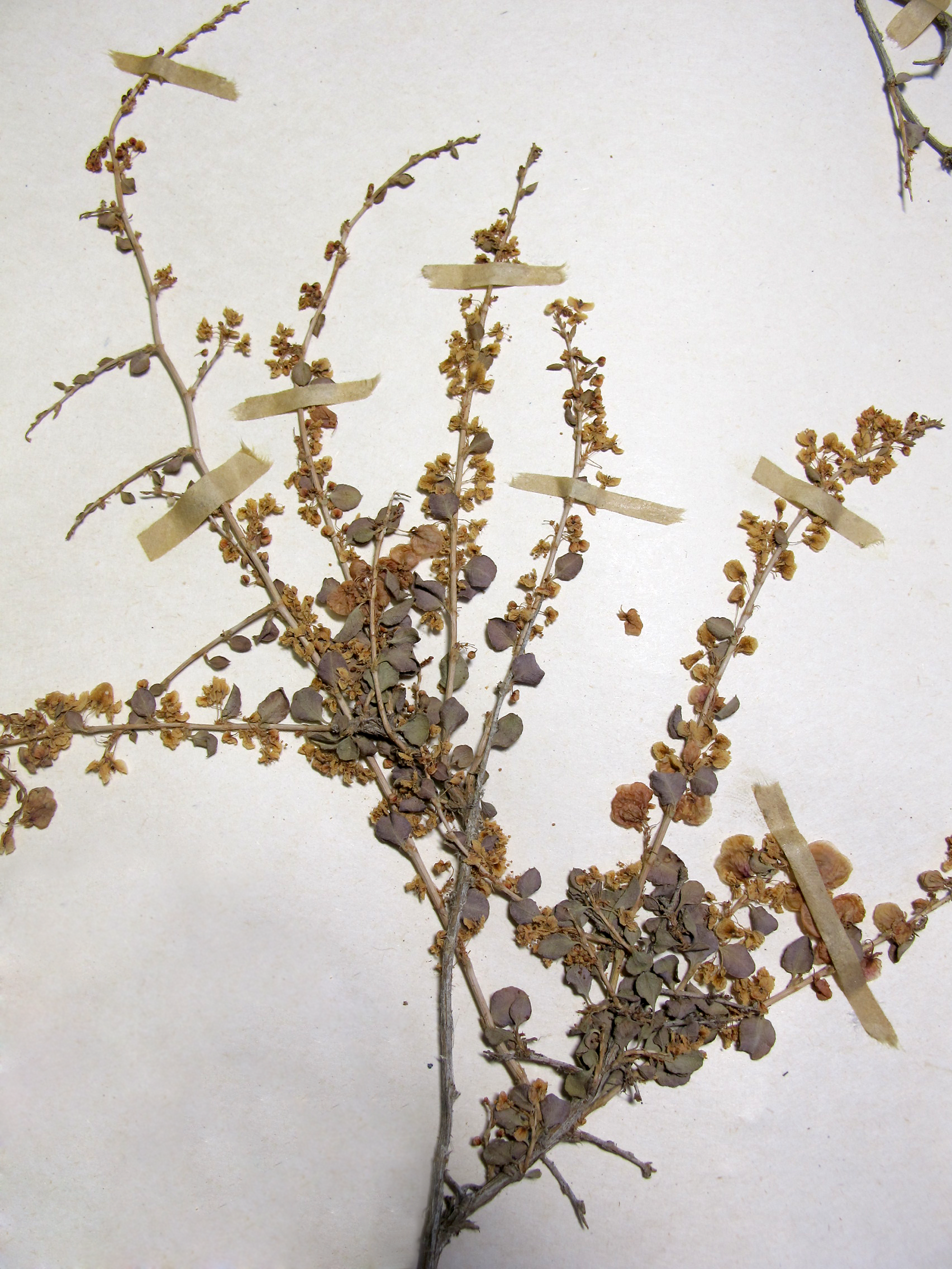

Supplement: Supplemental Information 21 — A shrub with elongated annual shoots and axillary short thyrses. Images: O. Yurtseva. [file peerj-04-1977-s021.png]
